# A Base‐Free Two‐Coordinate Oxoborane

**DOI:** 10.1002/anie.202419094

**Published:** 2024-11-26

**Authors:** Clement R. P. Millet, Dominic R. Willcox, Gary S. Nichol, Cate S. Anstöter, Michael J. Ingleson

**Affiliations:** ^1^ EaStCHEM School of Chemistry University of Edinburgh Edinburgh EH9 3FJ United Kingdom

**Keywords:** Boron, Boroxine, Oxoborane, Carbonyl compounds, multiple bonding

## Abstract

Oxoboranes (R‐BO) are transient species that rapidly trimerise to form boroxines. To date, the only method used to stabilise oxoboranes is to add a Lewis base, but this forms a three‐coordinate at boron oxoborane that has a different bonding/reactivity profile. Herein we report a base‐free, two‐coordinate oxoborane that is isolated as a Lewis adduct with AlCl_3_. This species, Mes*BO‐AlCl_3_ (Mes*=2,4,6‐^*t*^Bu‐C_6_H_2_), has a ν^11^ΒΟ stretching frequency of 1843 cm^−1^, indicating a strong BO bond. Computational analysis indicates this is due to a highly polarised BO bonding interaction combined with modest BO multiple bond character. While the polarisation of the BO bond on AlCl_3_ coordination enhances the Lewis acidity at boron it also reduces the basicity at oxygen and the latter is key to accessing a base‐free oxoborane. Finally, this oxoborane reacts with PhN_3_ in a unique way to form an unprecedented boron heterocycle.

## Graphical abstract

A base‐free, two coordinate oxoborane (R‐BO) has been isolated as a Lewis adduct with AlCl_3_ and shown to react in a unique way with PhN_3_. In contrast to the parent oxoborane, the oxoborane→AlCl_3_ adduct is stable towards oligomerisation and C−H cleavage of the Mes* group as AlCl_3_ coordination provides thermodynamic stabilization while also reducing the basicity of the oxygen unit thus reducing the biphilic character of the BO unit.

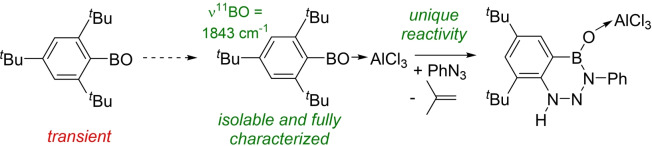

## Introduction

**Figure 1:**
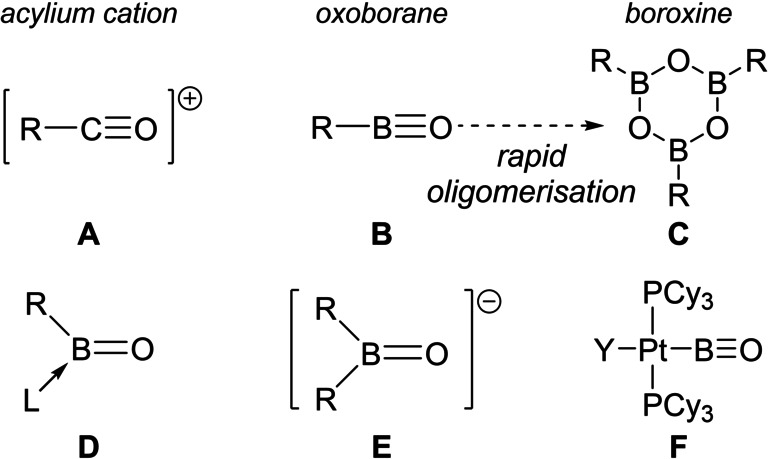
Select previously reported R‐CO or R‐BO species, for **F** Y=Br/PhS.

Carbonyl units are a key functional group in organic chemistry, present in ubiquitous classes of compounds including ketones (R_2_C=O) and acyl‐halides (R(X)C=O). The removal of halide from the latter yields acylium cations, [R−C≡O]^+^ (**A**, Figure 1, R=alkyl or aryl),^[1]^ which are important electrophiles widely used in Friedel–Crafts acylations.^[2]^ While a range of acylium salts have been isolated and characterised,[^1^, ^2^] the isoelectronic boron species, oxoboranes (**B** Figure 1),^[3]^ only have been observed in the gas phase or using low temperature matrix isolation techniques.[^4^, ^5^] Under more standard conditions oxoboranes oligomerise to form boroxines ((RBO)_3_ **C**).^[6]^ The disparity in the relative stability of **A** and **B** towards oligomerisation is due to the greater polarisation of the BO π bonds (relative to the CO π bonds)^[3]^ and the absence of a unit positive charge in oxoboranes, with this positive charge disfavouring the oligomerisation of acylium cations. To date, to access isolable monomeric oxoborane derivatives researchers used systems where boron is three‐coordinate, achieved by incorporating a neutral Lewis base, L (to form L(R)B=O, **D**),[^7^, ^8^, ^9^, ^10^, ^11^, ^12^, ^13^, ^14^, ^15^] or an anionic group (to form [R_2_B=O]^−^, **E**).[^16^, ^17^, ^18^, ^19^] However, **D** and **E** (Figure 1) are boron analogues of ketones not acylium cations, thus have a reduced BO bond order relative to that in **A** and **B**. While there is a seminal report on a two coordinate at boron metal‐oxoboryl complex (**F**, Figure 1),^[20]^ this is a boron analogue of a metal‐carbonyl complex. In this complex there is significant BO multiple bond character (bond index calculated to be 2.83), while two Pt 5d→2p B π donor interactions contribute to the stability of the two‐coordinate boron centre. In contrast, no isolable base‐free, two‐coordinate at B, organo‐oxoborane has been reported to date.

**Figure 2:**
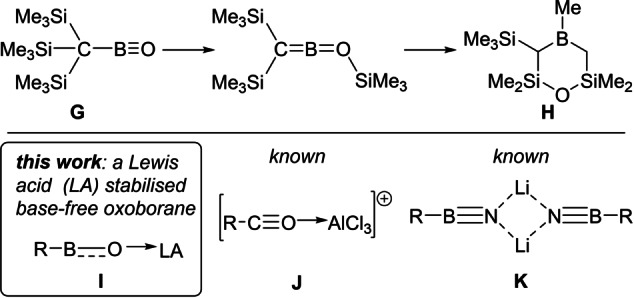
Top, decomposition of an organo‐oxoborane. Bottom, inset this work on forming a LA stabilised oxoborane and the related compounds **J** and **K**.

One method to stabilise multiple bonds in main group chemistry is to use bulky substituents (termed kinetic stabilisation). However, when applied to oxoboranes this still results in oligomerisation^[21]^ or intramolecular reactions (e.g., conversion of **G** to **H**, Figure 2 top).[^22^, ^23^] We hypothesised that the significant π bond polarisation of the BO unit in **G** led to sufficient basicity at oxygen to result in the observed reaction. Therefore, reducing the basicity at oxygen in oxoboranes by coordination of a Lewis acid (LA) could stabilise two coordinate oxoboranes and enable isolation of compounds of general formula **I** (Figure 2 inset). In addition, the formation of a O→LA dative bond provides additional thermodynamic stabilisation.^[24]^ Note, isoelectronic (to **I**) carbon analogues derived from addition of AlCl_3_ to acylium cations (e.g., **J**, Figure 2) have been reported, although they are superelectrophiles.^[25]^ Furthermore, another compound class related to **I**, bora‐imino‐lithium (**K**, Figure 2), have been reported recently.^[26]^ Notably, **K** contains significant dative N→Li interactions in addition to NB π bonding. While **J** and **K** have R−E=Y units isoelectronic to that in **I** they both have more effective π bonding than that expected for **I** (based on the relative electronegativities of B, C, N and O).

Thus, at the onset of this work it was not clear what the electronic structure of **I** would be (including the BO bond order) nor how reactive it would be. Herein, we report that by combining kinetic and thermodynamic stabilisation it is possible to isolate a base‐free, two coordinate oxoborane as an AlCl_3_ adduct.

## Results and Discussion

**Scheme 1:**
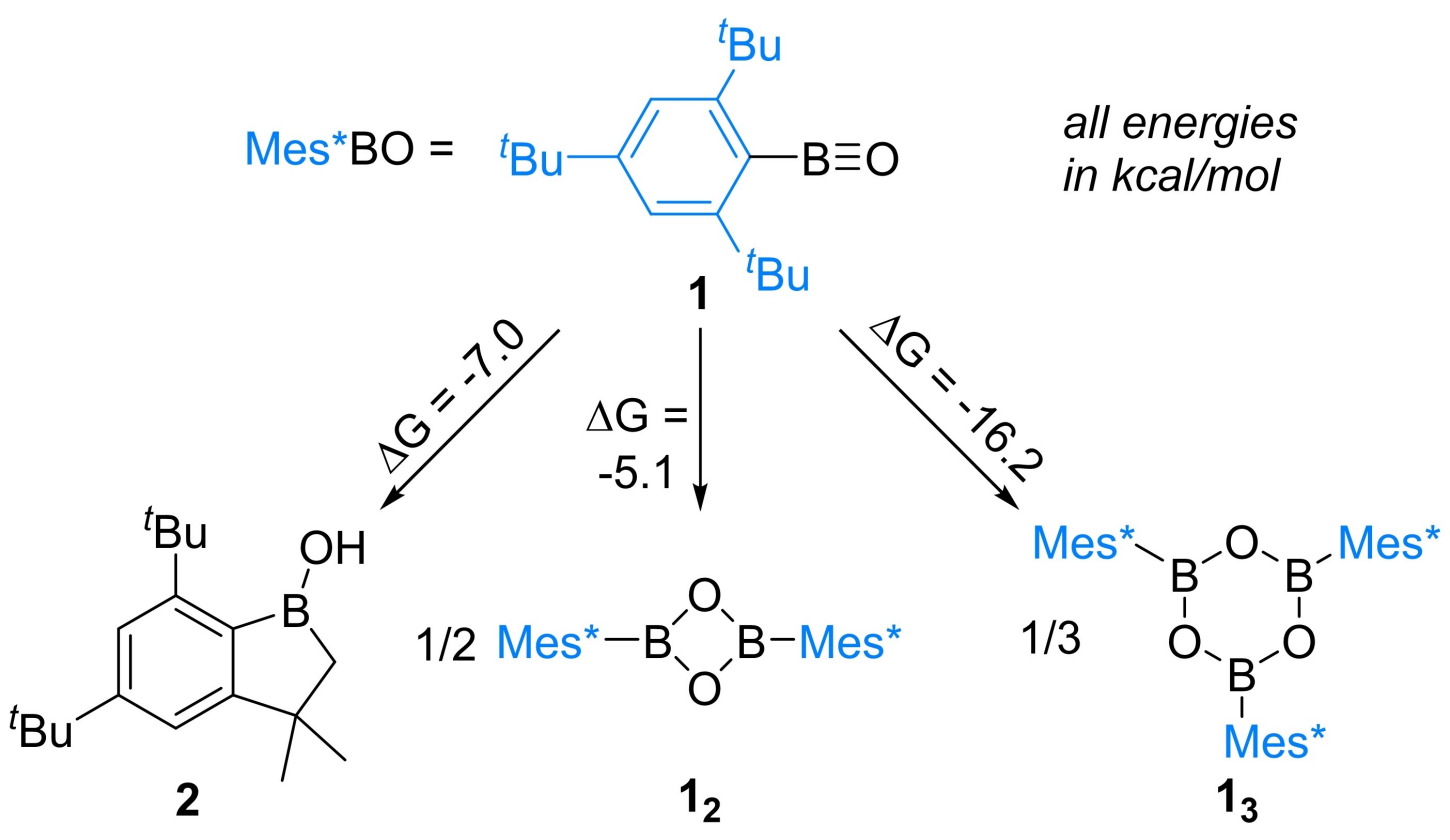
The previously reported conversion of **1** to **2** and **1_2_**, and the respective energies for the formation of **2** and two oligomers of **1**.

This study focused on targeting derivatives of Mes*BO, **1** (Mes*=2,4,6‐^*t*^Bu‐C_6_H_2_, Scheme 1). Two previous reports documented different outcomes from the attempted syntheses of **1**. Paetzold and Groteklaes reported that **1** is transient and reacts via cleavage of a proximal C−H bond to form **2**.^[23]^ In contrast, West and Pachaly reported that attempts to make **1** via a different approach led to formation of the dimer, **1_2_** (Scheme 1).^[27]^ Our calculations (all at the MN15 (SMD: C_6_H_6_)/Def2‐TZVPP//PBE0‐D3(BJ)(SMD: C_6_H_6_)/Def2‐SVP level) indicated that while formation of the dimer **1_2_** is favoured starting from two equivalents of monomer (by 5.1 kcal/mol), the formation of the C−H cleaved product **2** is more exergonic. Notably, formation of the boroxine from trimerisation of **1**, termed **1_3_**, is thermodynamically the most favoured outcome out of the three possibilities. These calculations indicated that if accessible **1_2_/1_3_** could act as a source of monomeric **1**. Given that the coordination of aluminium trichloride to boroxines was calculated to be thermoneutral (see Figure S44), we hypothesised that combining **1_2_**/**1_3_** with a Lewis acid could enable trapping of a transient monomeric oxoborane by adduct formation. Therefore, attempts were made to form **1_2_** or **1_3_**.

**Scheme 2:**
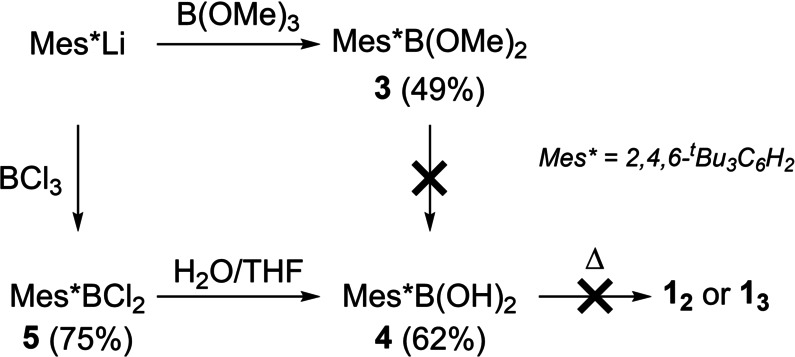
Attempted formation of **1_2_** or **1_3_** by the thermolysis of **4**.

The report from West and Pachaly stated that the in situ hydrolysis of Mes*B(OMe)_2_, **3** (Scheme 2), and subsequent heating (65 °C at 0.07 mbar) leads to **1_2_**.^[27]^ However, **3** does not undergo hydrolysis to the boronic acid Mes*B(OH)_2_, **4**, under a range of conditions (see section S3.1). Forcing conditions led only to slow protodeboronation of **3** to form Mes*H, with no **4** observed. Therefore, a route to access **4** was developed proceeding from Mes*BCl_2_, **5**. In contrast to **3**, compound **5** does undergo hydrolysis to afford the boronic acid **4**. However, subjecting **4** to a range of conditions did not lead to formation of **1_2_** or **1_3_**, with no dehydration occurring even at 180 °C and 0.01 mbar. Thus, it appears that **1_2_/1_3_** cannot be formed from **4**, in contrast to what was previously reported.^[27]^

**Figure 3:**
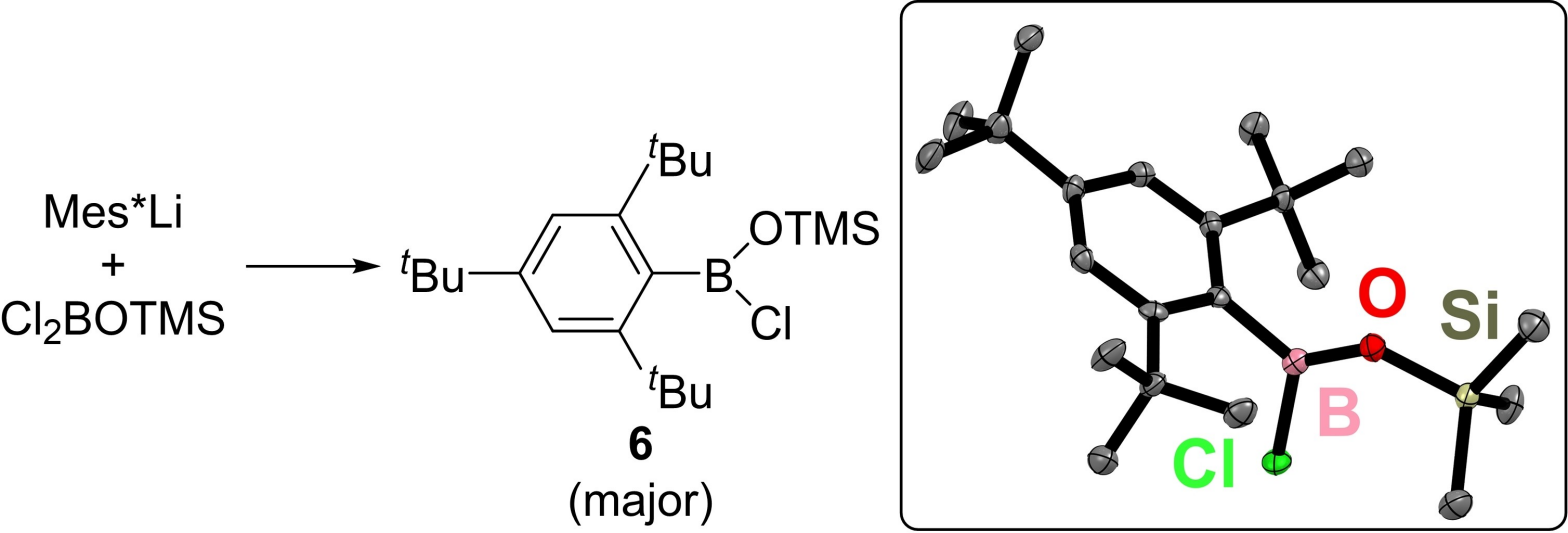
Left, the synthesis of **6**. Right, the solid‐state structure of **6**, ellipsoids at 50 % probability with all hydrogens omitted for clarity.

Therefore, an alternative procedure to access a Lewis acid adduct of **1** was developed. This built on the precedence of forming B=O containing Lewis adducts from a precursor predisposed to lose TMSX (TMS=Me_3_Si).^[15]^ Mes*BCl(OTMS), **6** was synthesised from the addition of Mes*Li to Cl_2_BOTMS (Figure 3). The identity of **6** was confirmed by single crystal X‐ray diffraction studies.^[28]^ The structure of **6** (inset Figure 3) is unremarkable, containing bond lengths in the expected range (e.g., B−O, B−C and Si−O=1.321(6), 1.595(6) and 1.691(3) Å, respectively). However, the isolation of pure **6** on scale proved problematic, with low quantities of impurities (such as Mes*BCl_2_ and MesH at ca. 5–10 %) present that frustrated all attempts at separation. Furthermore, other routes to **6** (e.g., from Mes*BCl_2_) were not successful, therefore **6** was carried forward containing low quantities of these impurities.

**Figure 4:**
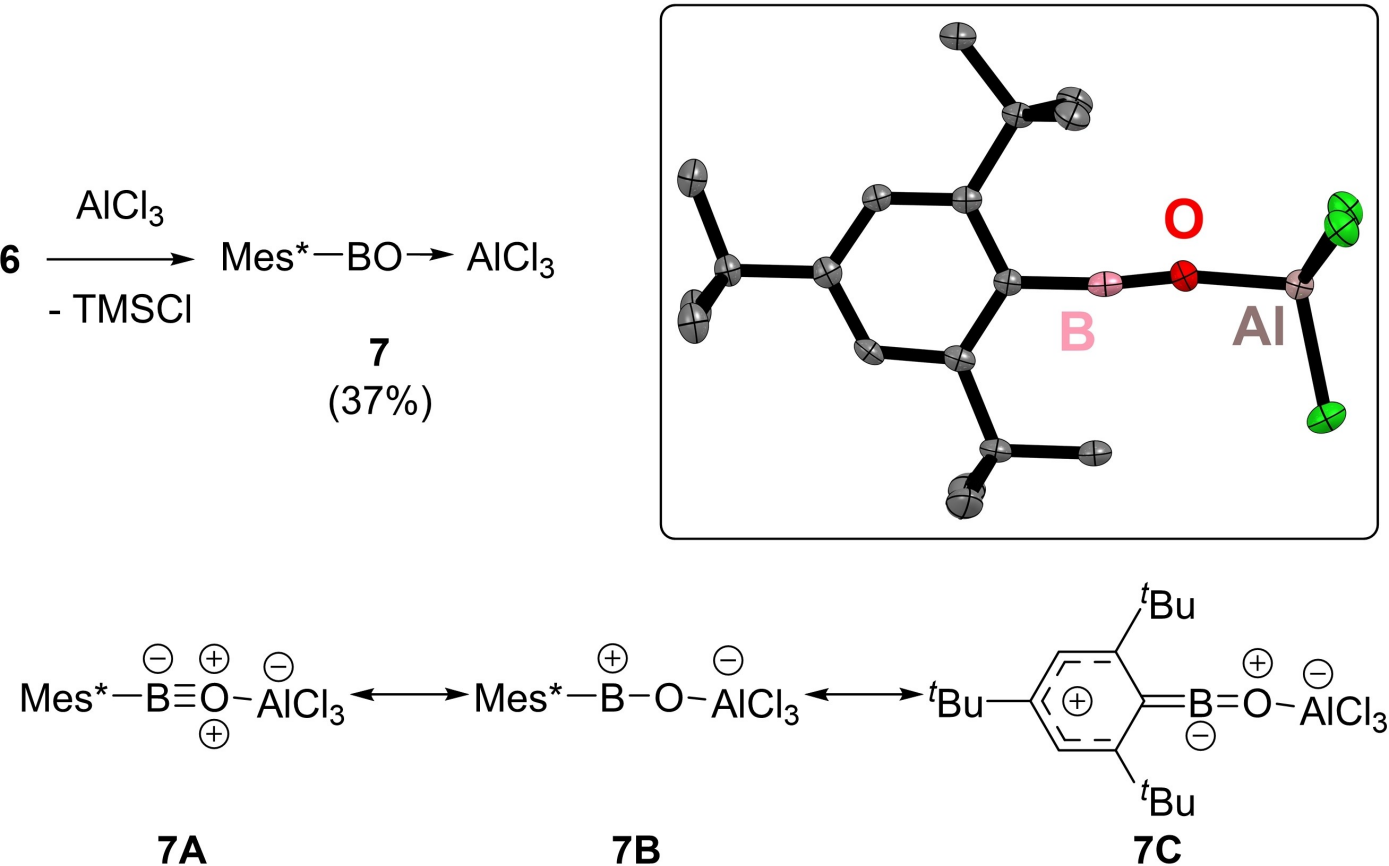
Top left, synthesis of **7** and inset the solid‐state structure of **7** with ellipsoids at 50 % probability and all hydrogens omitted for clarity. Bottom, select resonance forms for **7** with formal charges. Select bond lengths (Å) and angles for **7** (°): Al−O=1.787(2), B−O=1.232(4), B−C=1.505(5), Al−Cl=2.1047(13)/2.1182(8), Al−O−B=169.7(2) and O−B−C=173.0(3).

While compound **6** was stable to loss of TMSCl at room temperature, heating **6** in *o*‐dichlorobenzene led to the formation of multiple (>5) species (by ^11^B and ^29^Si NMR spectroscopy). Notably, none of these corresponded to **1_2_**, **1_3_**, or **2**. Next, Lewis acid reagents that can catalyse loss of TMSCl from **6** were explored. The addition of a small excess (1.2 equivalents) of AlCl_3_ to **6** led to the formation of one major new boron containing compound along with TMSCl (by ^29^Si NMR spectroscopy). This new boron containing compound could be isolated in 37 % yield. Analysis by single crystal X‐ray diffraction studies revealed it to be the base‐free, two‐coordinate at boron, oxoborane‐AlCl_3_ Lewis adduct Mes*BO→AlCl_3_, **7** (Figure 4). The structure of **7** contains a two‐coordinate boron centre with the closest intermolecular interactions being to two chlorides from adjacent molecules of **7**, at 3.294 Å. While these two contacts are within the combined van der Waals radii of boron and chlorine (1.92 and 1.75 Å, respectively),^[29]^ they are considerably longer than the combined covalent radii (0.84 and 1.02 Å, respectively).^[30]^

While multiple resonance structures can be proposed for compound **7** (Figure 4 shows just three of these), the solid‐state metrics for **7** are consistent with a structure where the BO and BC units have some multiple bond character. This includes an almost linear C−B−O unit (C−B−O=173.0(3)°), indicating an sp hybridised boron centre, and a very short BO distance of 1.232(4) Å which is comparable to that in the Pt‐BO complex **F** (BO distance=1.210(3) Å for Y=SPh)^[20]^ and close to that determined for gaseous H‐BO (1.2004(3) Å).^[31]^ Notably, the BO distance in **7** is considerably shorter than that in three‐coordinate at boron oxoborane→AlCl_3_ adducts where BO distances are ca. 1.31 Å.[^7^, ^8^, ^9^, ^10^] Compound **7** also has a longer Al−O bond (in **7**, Al−O=1.787(2) Å) relative to that in three‐coordinate at boron oxoborane→AlCl_3_ adducts (where Al−O≈ 1.71 Å).[^7^, ^8^, ^9^, ^10^] This indicates that **7** has a stronger BO bond and a weaker O−Al interaction relative to that in three‐coordinate at boron oxoborane→AlCl_3_ adducts. Several resonance structures for **7** have C=B double bond character (e.g. **7 C**, Figure 4), with related resonance structures previously proposed for aryl acylium cations, [Ar−C≡O]^+^.[^1^, ^2^] While the B−C bond in **7** is shorter (at 1.505(5) Å) than that in other two‐coordinate at boron Mes*B containing compounds, e.g. **K** (Figure 2, BC=1.553(4) Å),^[26]^ Mes*BC≡N−N C≡B‐Mes* (BC=1.528(3) Å)^[32]^ and Mes*BC≡N^*t*^Bu (BC=1.519(6) Å),^[33]^ it is longer than that found in the two‐coordinate at boron [Mes_2_B]^+^ cation (where BC=1.459(3) Å, Mes=mesityl).^[34]^ Therefore while there is some BC multiple bond character in **7** it is less than that between ^Mes^CB in [Mes_2_B]^+^ presumably due to the presence of some BO π bonding in **7**.

Given that **7** is stable in chlorobenzene and benzene we assessed its speciation in solution. DOSY NMR spectroscopy was consistent with **7** being a monomer in solution, while the solution ^11^B chemical shift for **7** (27.6 ppm) is similar to that reported for related two coordinate boron compounds, specifically the Pt‐BO complex **F** (δ_11B_=17)^[20]^ and the boron cation [(R_2_N)BO−SiMe_3_]^+^ (δ_11B_=22, R_2_N=2,2,6,6‐tetramethylpiperidide).^[35]^ Furthermore, the δ_11B_ chemical shift for **7** is comparable to that predicted for monomeric **7** by DFT calculations (δ_11B_ 31.7). The ν^11^ΒΟ stretching frequency measured in the solid‐state (1830 cm^−1^) was comparable to that observed in benzene (1843 cm^−1^) also consistent with a monomeric solution formulation. The observed ν^11^BO for **7** is significantly greater than that reported for three coordinate at boron oxoboranes (ν^11^BO ca. 1600 cm^−1^)[^7^, ^8^, ^9^, ^10^] and is comparable to that reported for **F** (ν^11^BO=1797 cm^−1^).^[20]^ However, The ν^11^BO for **7** is lower than that reported at 10 K for Ph−BC≡O (ν^11^BO 1975 cm^−1^).^[5]^ This indicates that the binding of a Lewis acid to oxygen in organo‐oxoboranes lowers the BO bond order (see below). This was supported by DFT calculations revealing a reduction in the ν^11^BO of 83 cm^−1^ on binding of AlCl_3_ to Mes*BO, **1** (1995 cm^−1^ calculated for **1** and 1912 cm^−1^ calculated for **7**). Note, an analogous reduction has been reported for metal carbonyl complexes on binding aluminium Lewis acids.^[36]^

**Figure 5:**
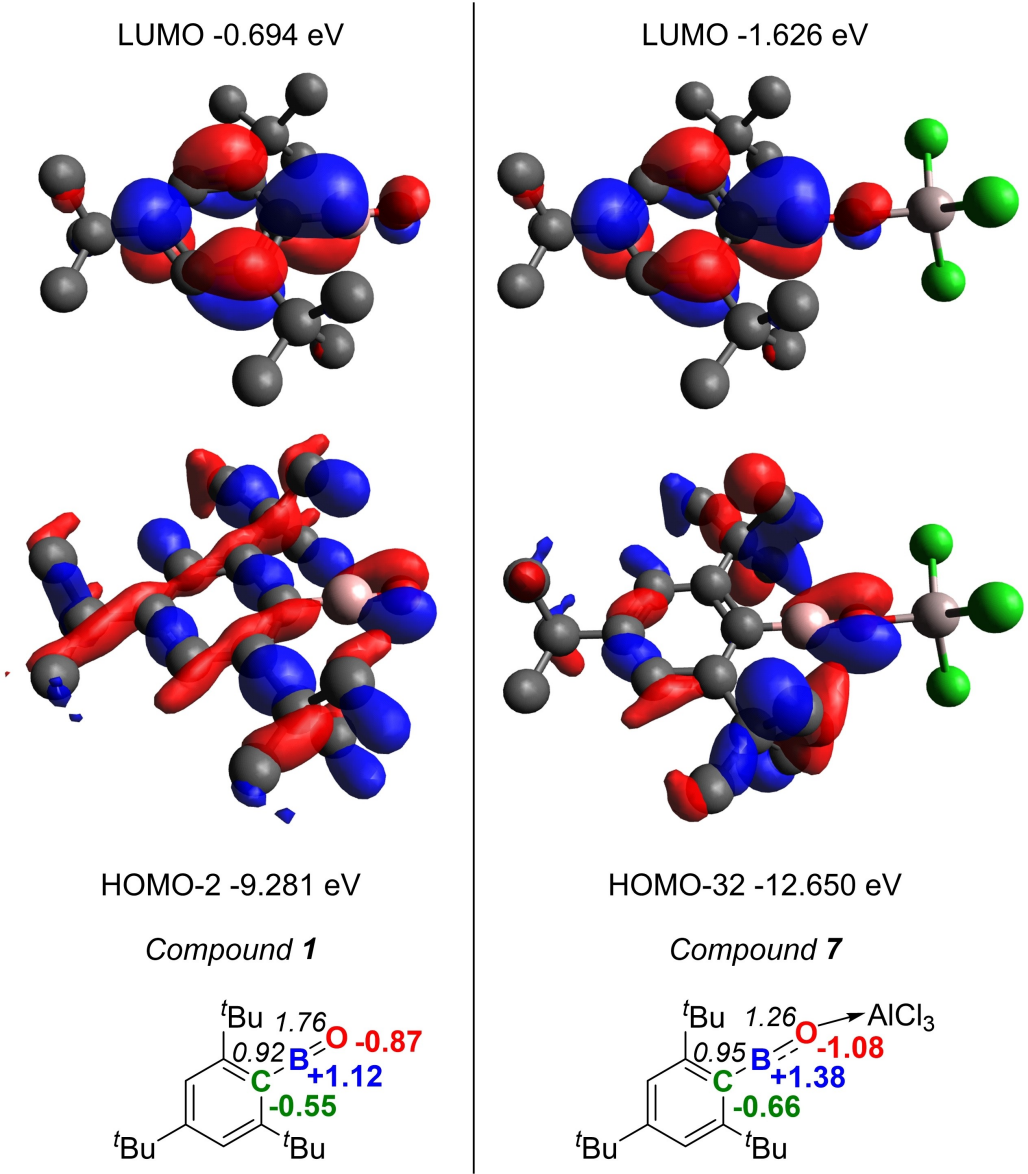
Top, LUMO for **1** (left) and **7** (right). Middle HOMO with BO π bonding character for **1** (left) and **7** (right). Isosurface=0.04. Hydrogens omitted for clarity. Bottom, NPA charges (in colour) and WBI for CB and BO (in black italics).

To gain more insight into the electronic structure and bonding of **7**, DFT calculations coupled with natural bond orbital (NBO) analyses were performed. Note, the optimised structure of **7** compares well with that in the solid‐state. While the LUMO (Figure 5 top right) and LUMO +2 of **7** have significant BO π* character, the orbitals that contain BO π bonding character are much lower in energy and are polarised towards oxygen. The highest energy orbital containing any significant BO π bonding character is HOMO‐32 for **7** (Figure 5, middle right). For Mes*BO (**1**), while the LUMO is similar in character to that of **7**, the orbitals with BO π bonding character for **1** are much higher in energy (e.g., HOMO‐2, Figure 5 left middle). The fact that the MOs for **7** with BO π bonding character are deep in energy is reminiscent to that calculated for the BF unit in the terminal boron‐monofluoride iron complex, Fe(BF)(CO)_2_(CNAr)_2_.^[37]^ In this complex the BF‐π bonding orbitals are also highly polarised to F and are very deep in energy.

The NPA charges for **7** also indicate significant polarisation in the CBO unit (Mes*C_ipso_=−0.66, B=+1.38 and O=−1.08). While the combined charge on the AlCl_3_ unit is slightly negative (at −0.133) consistent with an O→Al dative bond. Comparison to the NPA charges for Mes*BO, **1**, is informative (Figure 5 bottom left) and reveals that **7** has the more polarised BO unit. This is consistent with compound **1** having a shorter BO bond (1.213 Å) than **7** and is also consistent with the Wiberg bond indices (WBI), for BO in **7** the WBI is 1.26, while for BO in **1** the WBI is 1.76. This significant reduction in BO multiple bond character in **7** relative to that in **1** is not compensated by an increase in CB π bonding, with the WBI for CB in **7** (0.95) being comparable to that for CB in **1** (0.92). Combined these observations are consistent with a highly polarised structure containing only a modest degree of BO π bonding (i.e. the electronic structure lies closer to **7B** than to **7 A** (Figure 4) due to the significantly polarised BO π bonds). This is again reminiscent of the bonding within the BF unit in the terminal Fe‐(BF) complex, in this complex the WBI for BF is only 0.87 despite the BF bond being extremely short (1.2769(29) Å).^[37]^ In this report, the short BF bond was attributed to a highly polarised BF interaction with minimal contribution from π bonding. As Lewis acid coordination to the oxoborane localises electron density at oxygen (relative to that in **1**), this makes the BO unit in **7** similar in character to a BF unit. Lewis acid binding to O also leads to a more electron deficient boron centre consistent with the LUMO energies of **7** and **1** being −1.63 eV and −0.69 eV, respectively. This difference in LUMO energies coupled with the greater positive charge localised at boron in **7** will mean compound **7** is significantly more Lewis acidic than compound **1**.

The successful isolation of **7** raised multiple questions, including: (i) why does **7** exist as a stable monomer when Lewis acid free oxoboranes undergo decomposition by internal reaction (e.g. **G** forming **H**) or oligomerise to form boroxines? (ii) Why is **7** stable in benzene when related carbon electrophiles of general formula **A** and **J** (Figure 1 and 2) readily effect the acylation of arenes (including benzene and toluene).[^1^, ^2^]

**Scheme 3:**
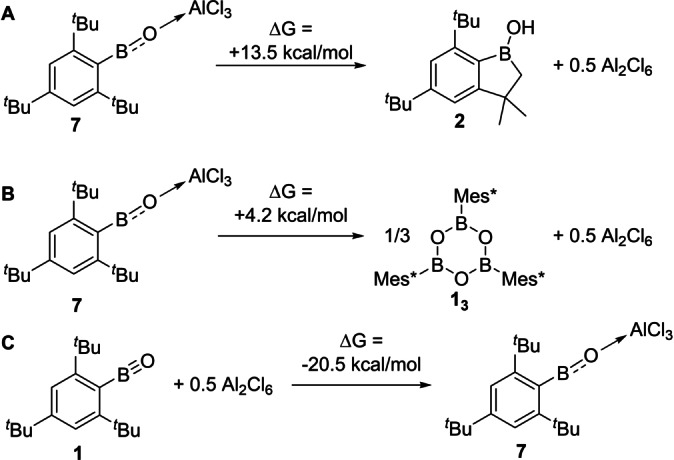
A–C. Free energy change calculated for select reactions.

Considering (i), calculations confirmed that C−H cleavage to form **2** from **7** is endergonic (Scheme 3A) in contrast to the outcome starting from **1** (Scheme 1).^[38]^ Given the deeper LUMO of **7** relative to the LUMO in **1**, this difference is attributed to AlCl_3_ coordination attenuating the basicity at O. This leads to a much lower energy HOMO with BO π bonding character for **7** (−12.65 eV) relative to that in **1** (−9.28 eV). Thus, Lewis acid coordination at O reduces the biphilic character of the oxoborane BO unit. As biphilicity is key for enabling C−H bond cleavage by main group species,^[39]^ this is presumably why oxoborane **1** rapidly converts to **2** whereas Lewis adduct **7** is stable towards a related C−H cleavage event.

The observation that **7** does not oligomerise is also reproduced by the calculations (Scheme 3B), and again is in contrast to that calculated for **1** (Scheme 1). This is consistent with the aforementioned thermodynamic stabilisation provided by RBO→AlCl_3_ adduct formation This was supported further by the reaction between Mes*BO and 0.5 equiv. Al_2_Cl_6_ being considerably exergonic (Scheme 4C). Thermodynamic stabilisation alone is not sufficient to prevent oligomerisation, as trimerisation of the phenyl analogue of **7**, PhBO→AlCl_3_, to form the boroxine (PhBO)_3_ and 1.5 equivalents of Al_2_Cl_6_ is calculated to be exergonic (see Figure S44). Therefore, the stability of **7** towards oligomerisation is due to a combination of kinetic and thermodynamic stabilisation.

**Figure 6:**
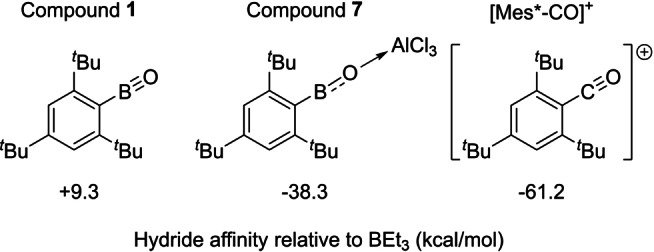
Calculated hydride affinity (ΔG) relative to BEt_3_.

Moving to question (ii), the Lewis acidity of Mes*BO→AlCl_3_ (**7**), Mes*BO (**1**) and the carbocation [Mes*CO]^+^ towards hydride was determined. This was selected as the hydride affinity is a parameter that has been previously reported to correlate well with S_E_Ar reactivity.^[40]^ Consistent with studies on other oxoboranes,^[41]^ **1** is a weak Lewis acid towards hydride, having a Lewis acidity towards hydride less than that of BEt_3_. The binding of AlCl_3_ to **1** to form **7** dramatically increases the Lewis acidity towards hydride (by 47.6 kcal/mol, Figure 6). Compound **7** actually has a Lewis acidity towards hydride comparable to that of B(C_6_F_5_)_3_ and [CatB(NR_3_)]^+^ borenium cations (Cat=catecholato).^[40]^ Note, catecholato‐borenium cations also do not borylate benzene, consistent with the observation that **7** is stable in benzene.^[42]^ The related carbocation [Mes*CO]^+^ is considerably more Lewis acidic towards hydride than **7**, despite the fact that it does not contain a coordinated AlCl_3_. Further computational analysis on [Mes*CO]^+^ revealed significantly higher CO and CC WBIs (2.192 and 1.327, respectively) than for the analogous bonds in **7**. This indicates stronger EO and EC^Mes^* π bonding is present in [Mes*CO]^+^ relative to that in **7** (E=B or C). However, the LUMO in [Mes*CO]^+^ (which has significant CO π* character) is still much lower in energy (at −4.20 eV) than the LUMO in **7** (−1.63 eV). This deeper LUMO in [Mes*CO]^+^ is consistent with the high reactivity of acylium cations in S_E_Ar. It also demonstrates that a unit positive charge has a larger effect on frontier orbital energies than the change in EO π bonding (for E=B relative to E=C).

**Figure 7:**
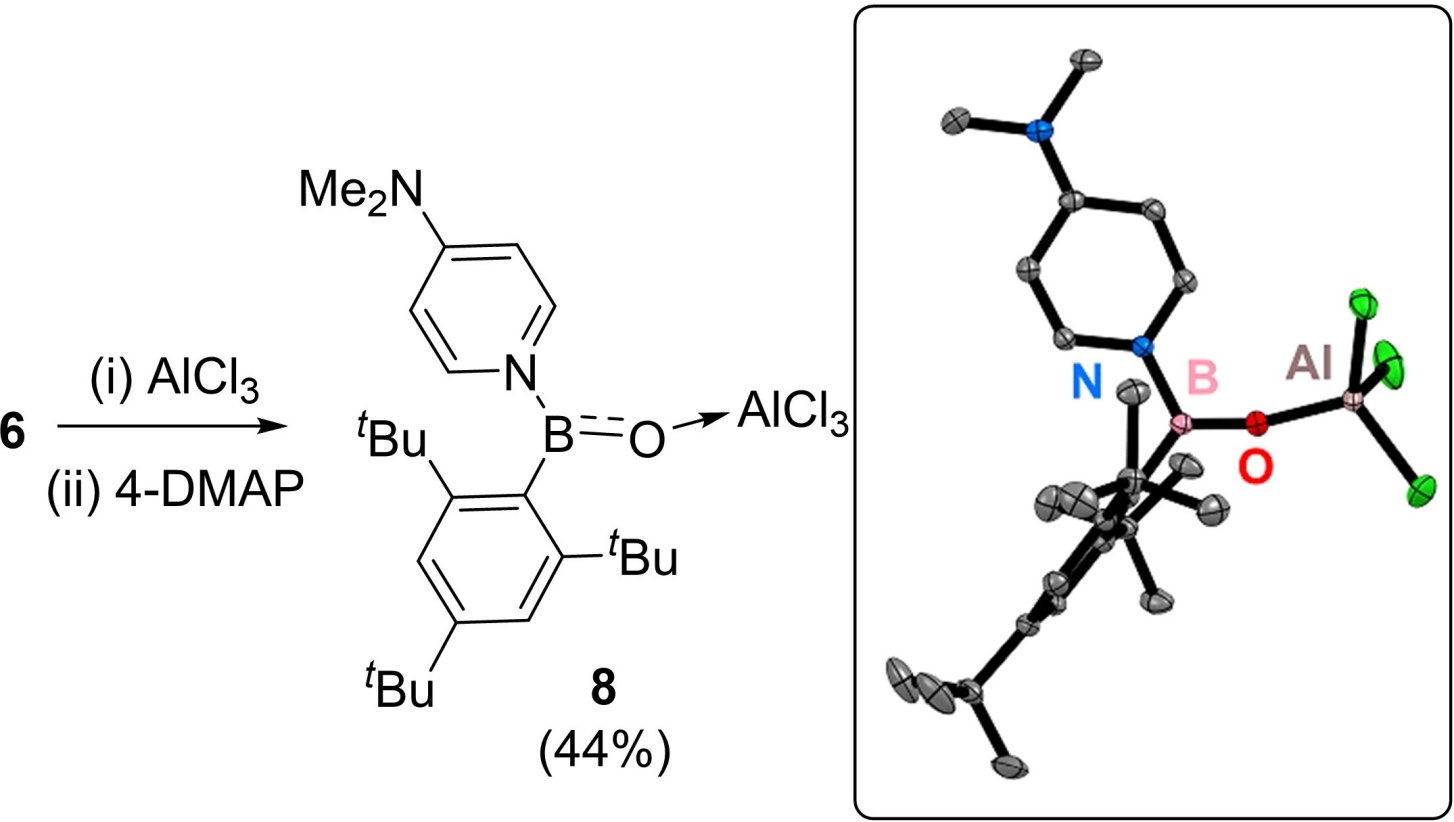
Left, the synthesis of compound **8**. Right the solid‐state structure of **8** ellipsoids at 50 % probability and hydrogens omitted for clarity. Select bond lengths (Å) and angles (°): Al−O=1.7168(10), B−O=1.3152(17), B−C=1.5822(18), B−N=1.5338(17), Al−Cl=2.1213(6)/2.1427(5)/2.1258(6), B−O−Al=165.68(10), O−B−C=124.91(12), O−B−N=117.23(11).

The above considers the Lewis acidic properties of **7** from a thermodynamic perspective. It is also conceivable that there is a kinetic barrier to react at the boron centre in **7** given the size of the Mes* substituent. Therefore, we investigated if **7** can function as a Lewis acid. The addition of one equivalent of 4‐DMAP to **7** (made in situ) led to one major new product displaying a downfield shifted broad ^11^B resonance (at 32 ppm). This product could be isolated in 44 % yield on crystallisation from chlorobenzene/pentane. Analysis by single crystal X‐ray diffraction revealed it to be the 4‐DMAP adduct **8** (Figure 7). In **8**, 4‐DMAP has bound to boron while the O→AlCl_3_ dative bond persists. The boron centre in **8** is effectively trigonal planar (angles at B Σ=359.9°) with a short B‐N_DMAP_ bond (1.5338(17) Å) and a contracted Me_2_N−C distance=1.3358(18) Å) consistent with some quinoidal character for the 4‐DMAP→B unit. Comparison of the metrics for **7** and **8** is informative and revealed that **8** has elongated BO (1.3152(17) Å) and BC (1.5822(18) Å) bonds, and a contracted AlO bond (1.7168(10) Å). For **8** these distances are comparable to other three coordinate oxoborane‐AlCl_3_ adducts,[^7^, ^8^, ^9^, ^10^] and indicate a weaker BO interaction in **8** relative to that in **7** (consistent with the lower v^11^BO measured for **8**=1639 cm^−1^). This leads to a more Lewis basic oxygen centre in **8** that binds to AlCl_3_ more strongly.

**Figure 8:**
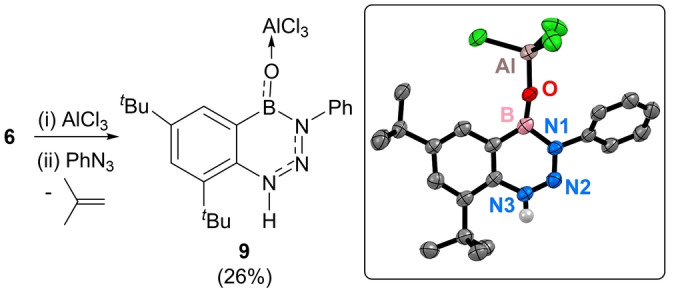
Left, the synthesis of **9**, inset right, the solid‐state structure of **9** ellipsoids at 50 % probability and all carbon bound hydrogens omitted for clarity. Select bond lengths (Å) and angles (°): Al−O=1.716(3), B−O=1.307(5), N1−N2=1.302(4), N2−N3=1.301(4), B−C=1.521(6), B−N=1.512(6), B−O−Al=170.9(3), C−N−N=128.3(7), N−N−N=118.2(3), N−N−B=124.5(3), N−B−C=111.8(4), N−B−O=118.5(4), O−B−C=129.7(4).

With boron centered Lewis acidity confirmed for compound **7** its reactivity towards PhN_3_ was explored next. PhN_3_ was selected as it is a low steric bulk Lewis base that displays rich reactivity with multiple bond containing boranes.^[43]^ Of particular relevance is the observation that boranes that contain *bona‐fide* triple bonds, specifically iminoboranes (such as Mes−N≡B−Mes), undergo cycloaddition with organoazides to afford tetraazaboroles.[^44^, ^45^] Thus, a BON_3_ five membered heterocycle would be expected if **7** reacted with PhN_3_ analogously to how iminoboranes react. However, the addition of one equivalent of PhN_3_ to **7** (made in situ) at room temperature in benzene does not form a BON_3_ heterocycle. Instead, it led to the formation of one major new boron containing species, compound **9** (Figure 8)_,_ along with isobutylene as a by‐product. This product could be isolated in 26 % yield. Analysis of **9** in CD_2_Cl_2_ revealed a broad N‐*H* resonance at 12.6 ppm along with a δ_11B_=24.8 and a δ_27Al_=89.3. Consistent with the observation of isobutylene as a by‐product the aliphatic region for **9** contained only two ^*t*^Bu resonances. Unambiguous identification of **9** was provided by single crystal X‐ray diffraction analysis (inset Figure 8). Analysis of the metrics of **9** revealed an essentially planar bicyclic structure containing two effectively identical NN bond lengths (1.301(4) and 1.302(4) Å) fully consistent with a triazene→borane Lewis adduct.^[46]^ Thus, **9** can be viewed as an internally Lewis base stabilized oxoborane→AlCl_3_ adduct. In **9** the BO (1.307(5) Å) and AlO (1.716(3) Å) distances are fully consistent with Lewis acid/base stabilized oxoboranes and are comparable to that found in **8**.

The solid‐state structure of **9** revealed that a significant rearrangement has occurred, as alongside loss of one ^*t*^Bu group nitrogen is bonded *ortho* to a ^*t*^Bu group and not boron (as is the case in **6** and **7**). A feasible mechanism for the formation of **9** that is supported by calculations (see Figure S43) proceeds by coordination of PhN_3_ to boron through the N_α_ position, followed by migration of the Mes* group from boron to the terminal nitrogen. This migration involves low barrier transition states (the highest being at +12.5 kcal/mol). A related boron to N_terminal_ aryl migration has been previously reported by Braunschweig and co‐workers post binding of PhN_3_ through N_α_ to a Lewis acidic diboron(4) compound.^[47]^ In the case of **7**+PhN_3_, a related migration generates an electrophilic boron centre that then effects intramolecular electrophilic borylation (with ^*t*^Bu^+^ the leaving group),^[48]^ this ultimately forms **9** and isobutylene (by deprotonation of ^*t*^Bu^+^ by the conjugate base of **9**) in a highly exergonic process. The dramatically different reactivity of PhN_3_ with **7** compared to PhN_3_ with iminoboranes (where a BN_4_ five‐membered heterocycle is formed from a reaction involving the B≡N unit)^[44]^ further highlights the distinct electronic structure of the BO unit in oxoborane **7** compared to the BN unit in iminoboranes. Finally, as the cyclic C_2_BN_3_ moiety in **9** represents a novel boron containing planar heterocycle a simplified model of **9** was probed using the ipsocentric approach (see Figure S45).[^49^, ^50^] This revealed that aromaticity is localised to the all‐carbon Clar's sextet (as indicated by the diatropic circulation) with a non‐aromatic BN_3_C_2_ cycle.

## Conclusions

By combining kinetic and thermodynamic stabilisation the first base‐free, two coordinate oxoborane, Mes*BO→AlCl_3_ (**7**), has been isolated under standard conditions. AlCl_3_ coordination to oxygen not only stabilises the monomeric oxoborane by preventing intramolecular C−H cleavage and oligomerisation, but it also results in a more polarised BO unit and thus there is only modest BO multiple bond character. This makes the bonding in the BO unit of **7** more reminiscent to that in a BF unit than that in an iminoborane. The two‐coordinate boron centre in **7** is much more Lewis acidic than the parent oxoborane (Mes*BO), and is comparable to B(C_6_F_5_)_3_ in hydride affinity. Thus, it reacts with low steric bulk Lewis bases, including PhN_3_. This results in an unprecedented boron heterocycle, further conforming the uniqueness of this base‐free oxoborane. Finally, we believe that Lewis acid coordination is an underexplored approach for stabilising otherwise transient oxoboranes compared to the much more common method of adding a Lewis base.

## Conflict of Interests

The authors declare no conflict of interest.

1

## Supporting information

As a service to our authors and readers, this journal provides supporting information supplied by the authors. Such materials are peer reviewed and may be re‐organized for online delivery, but are not copy‐edited or typeset. Technical support issues arising from supporting information (other than missing files) should be addressed to the authors.

Supporting Information

## Data Availability

The data that support the findings of this study are available in the supplementary material of this article.
