# Supplementary material for: A Base‐Free Two‐Coordinate Oxoborane
Source: Angew Chem Int Ed Engl. 2024 Nov 26;64(7):e202419094. doi: 10.1002/anie.202419094 (PMC11811692; doi:10.1002/anie.202419094)
Supplement: Supplementary file 1 — Supporting Information [file ANIE-64-e202419094-s001.pdf]

Angewandte Chemie
Eine Zeitschrift der Gesellschaft Deutscher Chemiker

Supporting Information

A Base-Free Two-Coordinate Oxoborane

*C. R. P. Millet, D. R. Willcox, G. S. Nichol, C. S. Anstöter, M. J. Ingleson**

A Base-Free Two-Coordinate Oxoborane

Clement R. P. Millet, Dominic R. Willcox, Gary S. Nichol, Cate S. Anstöter, Michael J. Ingleson*

School of Chemistry, University of Edinburgh, Edinburgh, EH9 3FJ.

Contents

1 General considerations S5

2 Compounds synthesis S6

2.1 Preparation of Mes*Br S6

2.2 Preparation of Mes*Li S6

2.3 Preparation of Cl_2BOTMS S7

2.4 Preparation of Mes*B(OMe)$_2$ S7

2.5 Preparation of Mes*BCl_2 .... S8

2.6 Preparation of Mes*B(OH)$_2$ S9

2.7 Preparation of 6, Mes*BCl(OTMS) S10

2.8 Preparation of 7, Mes*BO-$AlCl_3$ S11

2.9 Preparation of 8, Mes*(DMAP)BO-$AlCl_3$ S12

2.10 Preparation of 9 .... S14

2.11 NMR spectra of isolated compounds S16

2.11.1 NMR spectra of Cl_2BOTMS S16

2.11.2 NMR spectra of MesBCl_2 .... S17*

*2.11.3 NMR spectra of Mes*B(OH)$_2$ S18*

*2.11.4 NMR spectra of 6, Mes*BCl(OTMS) S19*

*2.11.5 NMR spectra of 7, Mes*BO-$AlCl_3$ S20*

2.11.6 NMR spectra of 8, Mes(DMAP)BO-$AlCl_3$ S22*

2.11.7 NMR spectra of 9 .... S24

2.12 IR and FTIR spectra S26

*2.12.1 IR and FTIR spectra of 7, Mes*BO-$AlCl_3$ S26*

2.12.2 IR and FTIR spectra of 8, Mes(DMAP)BO-$AlCl_3$ S27*

3 Preliminary studies S28

3.1 Attempts of synthesis of 1_2, [Mes*BO]$_2$, from Mes*B(OMe)$_2$ S28

*3.1.1 Attempt of synthesis of 1_2, [Mes*BO]$_2$, from reported route S28*

*3.1.2 Attempts of hydrolysis of Mes*B(OMe)$_2$ S28*

3.2 Dehydration attempts of Mes*B(OH)$_2$ S30

3.3 Attempt of thermal elimination of TMSCl from 6, Mes*BCl(OTMS) S32

4 Complementary studies S33

4.1 DOSY experiment of Mes*BO-$AlCl_3$, 7 .... S33

4.2 *In situ* NMR of generation of 9, observation of isobutylene S35

4.3 Equilibrium between 9 and an isomer, termed 9B S36

4.3.1 VT experiments S36

4.3.2 Equilibration in different solvents ... *S37*

4.3.3 Proposed structure and NMR data of 9B ... *S39*

5 Crystallographic data ... S41

5.1 Crystal structure of Mes*BCl(OTMS) 6 ... S41

5.2 Crystal structure of Mes*BO-$AlCl_3$ 7 ... S42

5.3 Crystal structure of Mes*(DMAP)BO-$AlCl_3$ 8 ... S43

5.4 Crystal structure of 9 ... S44

6 Computational details ... S45

6.1 Formation of 9 Free Energy Profile ... S46

6.2 Cartesian Coordinates ... S48

6.2.1 Compound 1 at PBE0-D3(BJ)/Def2-SVP(SMD: benzene) ... *S48*

6.2.2 Compound 1_2 ... *S50*

6.2.3 Compound 1_3 ... *S53*

6.2.4 Compound 2 ... *S57*

6.2.5 Compound 7 at PBE0-D3(BJ)/Def2-SVP(SMD: benzene) ... *S59*

6.2.6 Al_2Cl_6 ... *S61*

6.2.7 $[Mes^*CO]^+$ *at PBE0-D3(BJ)/Def2-SVP(SMD: benzene)* ... *S62*

6.2.8 $Et_2O{\cdot}BF_3$ ... *S64*

6.2.9 PhN_3 ... *S65*

6.2.10 TS1 ... *S66*

6.2.11 IM1 ... *S68*

6.2.12 TS2 ... *S70*

6.2.13 IM2 ... *S72*

6.2.14 TS3 ... *S74*

6.2.15 IM3 ... *S76*

6.2.16 Compound 9 ... *S78*

6.2.17 Isobutylene ... *S80*

6.2.18 9B-1 ... *S81*

6.2.19 9B-2 ... *S83*

6.2.20 9B-3 ... *S85*

6.2.21 $PhBO{\cdot}AlCl_3$ ... *S87*

6.2.22 $[PhBO]_3$ ... *S88*

6.2.23 $[PhBO]_3{\cdot}AlCl_3$ ... *S90*

6.2.24 BEt_3 ... *S92*

6.2.25 $[HBEt_3]^-$ ... *S93*

6.2.26 Compound 1 at M06-2X/6-311G(d,p)(IEFPCM: dichloromethane) ... *S94*

6.2.27 Compound $[1\text{-}H]^-$ ... *S96*

6.2.28 Compound 7 at M06-2X/6-311G(d,p)(IEFPCM: dichloromethane) *S98*
6.2.29 Compound [7-H]⁻ *S100*
*6.2.30 [Mes*CO]⁺ at M06-2X/6-311G(d,p)(IEFPCM: dichloromethane)* *S102*
*6.2.31 Mes*C(O)H* *S104*
6.3 Aromaticity study of 9 .... S106
6.3.1 Computational methods *S106*
6.3.2 Results & Discussion *S106*
6.3.3 Cartesian coordinates of the optimised structure *S107*
7 References S108

1 General considerations

All reactions were performed under inert conditions using standard Schlenk techniques or in an *MBraun Unilab* glovebox (<0.1 ppm H_2O / O_2).

Unless otherwise stated, solvents were degassed with nitrogen, dried over activated aluminium oxide (Solvent Purification System: Inert PureSolv MD5 SPS) and stored over 3 Å molecular sieves in ampoules equipped with J. Young's valves. Benzene and chlorobenzene were dried over calcium hydride, distilled and stored over 3 Å molecular sieves. Deuterated solvents ($CDCl_3$, C_6D_6, CD_2Cl_2 (99.6% D, Sigma Aldrich)) were dried and stored over 3 Å molecular sieves. All chemicals were, unless stated otherwise, purchased from commercial sources and used as received.

NMR spectra (^{1}H, ^{1}H{^{11}B}, ^{11}B, ^{11}B{^{1}H}, ^{13}C{^{1}H}, ^{27}Al and ^{29}Si) were recorded on *Bruker Avance III 400 MHz, Bruker Avance III 500 MHz, Bruker Avance III 600 MHz* or *Bruker PRO 500 MHz* spectrometers. Chemical shifts (δ) are quoted in parts per million (ppm), coupling constants (*J*) are given in hertz (Hz) to the nearest 0.5 Hz, and as positive values regardless of their real individual signs. ^{1}H and ^{13}C shifts are referenced to the appropriate residual solvent peak while ^{11}B shifts are referenced relative to external $BF_3{\cdot}Et_2O$, ^{27}Al shifts are referenced relative to external $Al(NO_3)_3$ and ^{29}Si shifts are referenced relative to external Me_4Si. Abbreviations used are s (singlet), d (doublet), t (triplet), q (quartet), sep (septet), m (multiplet), br (broad). Background signals in ^{11}B NMR spectra arise to a significant degree from glass components of the probes used in the spectrometers. Unless otherwise stated, all NMR spectra were recorded at 20 °C.

Mass spectrometry was performed by the *Scottish Instrumentation and Resource Centre for Advanced Mass Spectrometry* (SIRCAMS) at the University of Edinburgh using electron impact (EI) or electrospray ionisation (ESI) techniques.

IR and FTIR spectra were recorded on *Shimadzu IRSpirit QATR-S machine*.

2 Compounds synthesis

2.1 Preparation of Mes*Br

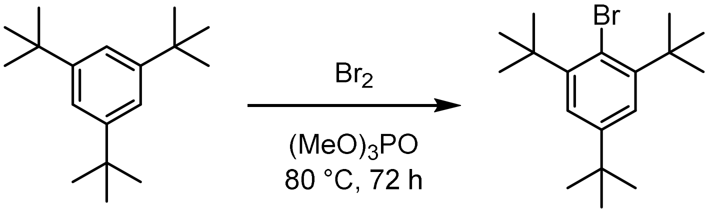

Mes*Br was synthesized according to a literature procedure.[S1]

Mes* (40.00 g, 162.32 mmol, 1.00 equiv.) was dissolved in $(MeO)_3PO$ (400 mL) and cooled to 0 °C in a round bottom flask. Br_2 (16.60 mL, 324.64 mmol, 2.00 equiv.) was added at 0 °C. The flask was equipped with a condenser and refluxed at 80 °C for 72 hours. The reaction mixture was cooled down to 0 °C and kept at this temperature for 4 hours. The crystalline solid that precipitated out of the solution was isolated by filtration and washed with cold ethanol (150 mL). Drying *in vacuo* afforded pure Mes*Br as colourless crystals in 75% yield (39.77 g, 122.25 mmol).

NMR data were in accordance with literature.[S1, S2]

^{1}H NMR (600 MHz, $CDCl_3$, 300 K) δ 7.43 (s, ^{meta}C-*H̲*, 2H), 1.60 (s, ^{ortho}C-C(C*H̲*$_3$)$_3$, 18H), 1.33 (s, ^{para}C-C(C*H̲*$_3$)$_3$, 9H).

^{13}C {^{1}H} NMR (151 MHz, $CDCl_3$, 300 K) δ 148.7 (s, ^{ipso}*C̲*-Br), 148.6 (s, ^{ortho}*C̲*- C(CH$_3$)$_3$), 123.8 (s, ^{meta}*C̲*-H), 121.8 (s, ^{para}*C̲*-C(CH$_3$)$_3$, 38.5 (s, ^{ortho}C- *C̲*(CH$_3$)$_3$), 35.1 (s, ^{para}C- C(*C̲*H$_3$)$_3$), 31.1 (s, ^{ortho}C- C(*C̲*H$_3$)$_3$).

2.2 Preparation of Mes*Li

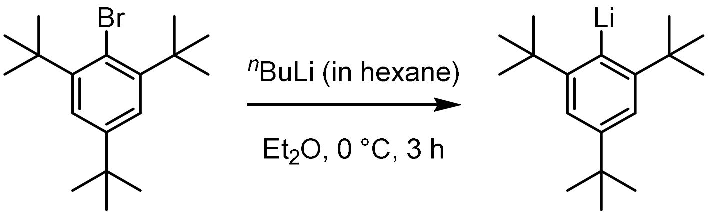

Mes*Li was synthesized according to a literature procedure.[S3]

^{n}BuLi (6.45 mL, C = 2.5 M in hexane, 16.14 mmol, 1.05 equiv.) was slowly added to a solution of Mes*Br (5 g, 15.37 mmol, 1 equiv.) in Et_2O (25 mL) at 0 °C over a period of 15 minutes. The solution was stirred for 3 hours at 0 °C. Volatiles were removed *in vacuo* and the residue was washed with pentane (2 x 10 mL). Drying *in vacuo* afforded Mes*Li as a white solid in 82% yield (3.20 g, 12.68 mmol).

NMR data were in accordance with literature.[S3]

^{1}**H NMR** (400 MHz, THF-h_8, 300 K) δ 6.93 (s, ^{meta}C-*H*, 2H), 1.26 (s, ^{ortho}C-C(C*H*$_3$)$_3$, 18H), 1.21 (s, ^{para}C-C(C*H*$_3$)$_3$, 9H).

^{13}**C {^{1}H} NMR** (126 MHz, THF-h_8, 300 K) δ 176.0 (s, ^{ipso}*C*-Li), 158.9 (s, ^{ortho}*C*- C(CH$_3$)$_3$), 142.0 (s, ^{para}*C*-C(CH$_3$)$_3$, 114.7 (s, ^{meta}*C*-H), 38.0 (s, ^{ortho}C- *C*(CH$_3$)$_3$), 33.9 (s, ^{para}C- *C*(CH$_3$)$_3$), 33.0 (s, ^{ortho}C-C(*C*H$_3$)$_3$), 31.5 (s, ^{para}C- C(*C*H$_3$)$_3$).

2.3 Preparation of Cl_2BOTMS

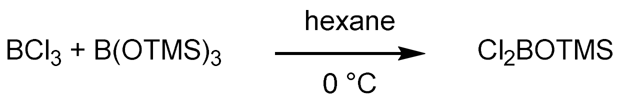

Cl_2BOTMS was synthesized according to a literature procedure.[S4]

B(OTMS)$_3$ (0.86 mL, 2.56 mmol, 1 equiv.) was added dropwise to a 1M solution of BCl_3 in hexane (5.00 mL, C = 1M, 5 mmol, 1.95 equiv.) at 0 °C. The solution was stirred for 2 hours at 0 °C. Cl_2BOTMS was obtained cleanly and was used from this stock solution. The stock solution was stored at –20 °C.

^{11}**B NMR** (160 MHz, hexane, 300 K) δ 28.6* (s, Cl$_2$*B*OTMS).

^{29}**Si NMR** (99 MHz, hexane, 300 K) δ 21.6* (s, *Si*(CH$_3$)$_3$).

* Despite being reported in the literature,[S4] no NMR data were available for this compound.

2.4 Preparation of Mes*B(OMe)$_2$

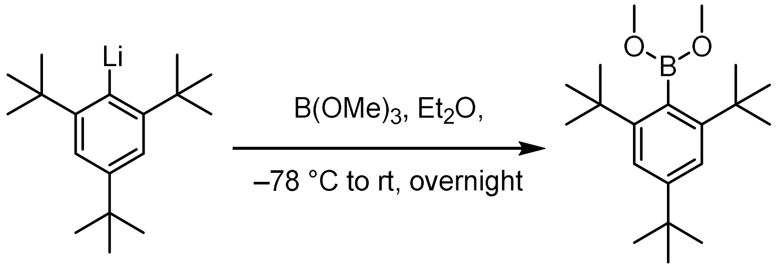

Mes*B(OMe)$_2$ was synthesized through a modified literature procedure.[S5]

B(OMe)$_3$ (0.46 mL, 4.16 mmol, 2.10 equiv.) was added dropwise to a solution of Mes*Li (0.50 g, 1.98 mmol, 1.00 equiv.) in Et$_2$O (6 mL) at –78 °C in a Schlenk flask. The solution was kept at –78 °C for 4 hours before being allowed to warm up to room temperature and stirred overnight. Volatiles were removed *in vacuo* and the residue was extracted with hot hexane (10 mL). Product was dried *in vacuo* and impurities removed by sublimation (100 °C, 3-4 x 10^{-2} mbar). Collection

of the residue afforded Mes*B(OMe)$_2$ as a colourless crystalline solid in 49% yield (0.31 g, 0.96 mmol).

NMR analysis were in accordance with literature.[S5]

^{1}H NMR (500 MHz, $CDCl_3$, 300 K) δ 7.58 (s, ^{meta}C-$\underline{H}$, 2H), 3.38 (s, OC$\underline{H}_3$, 6H), 1.51 (s, ^{ortho}C-C(C$\underline{H}_3$)$_3$, 18H), 1.36 (s, ^{para}C-C(C$\underline{H}_3$)$_3$, 9H).

^{11}B NMR (160 MHz, $CDCl_3$, 300 K) δ 32.5 (s, $\underline{B}$(OCH$_3$)$_2$).

^{13}C {^{1}H} NMR (151 MHz, $CDCl_3$, 300 K) δ 152.7 (s, $^{ortho}\underline{C}$- C(CH$_3$)$_3$), 149.6 (s, $^{para}\underline{C}$-C(CH$_3$)$_3$, 127.0* (s, $^{ipso}\underline{C}$-B(OCH$_3$)$_2$), 121.9 (s, $^{meta}\underline{C}$-H), 51.8 (s, O$\underline{C}$H$_3$), 38.0 (s, ^{ortho}C- $\underline{C}$(CH$_3$)$_3$), 35.0 (s, ^{para}C- $\underline{C}$(CH$_3$)$_3$), 32.3 (s, ^{ortho}C- C($\underline{C}$H$_3$)$_3$), 31.6 (s, ^{para}C- C($\underline{C}$H$_3$)$_3$).

* ^{ipso}*C*–B signal was observed by ^{1}H-^{13}C HMBC NMR experiment.

2.5 Preparation of Mes*BCl$_2$

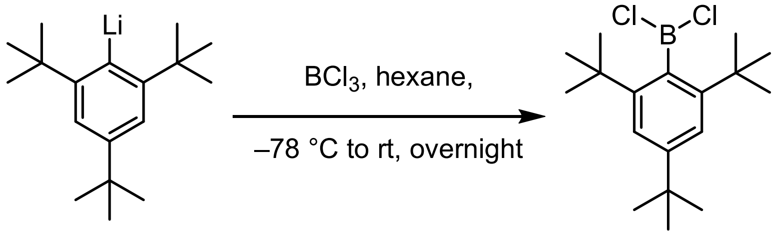

BCl_3 (5.00 mL, C = 1 M in hexane, 4.96 mmol, 2.50 equiv.) was added dropwise to a solution of Mes*Li (0.50 g, 1.98 mmol, 1.00 equiv.) in hexane (20 mL) at –78 °C. The solution was allowed to warm up to room temperature and stirred overnight. The solution was filtered and the solid residue extracted with pentane (3 x 2.5 mL). Volatiles were removed *in vacuo* affording a white solid as crude product. Impurities were removed by sublimation under heating (60 °C, 3-5 x 10^{-2} mbar, 1 hour). The residue was collected affording Mes*BCl$_2$ as a white solid in 54% yield (0.35 g, 1.06 mmol).

^{1}H NMR (400 MHz, C_6D_6, 300 K) δ 7.50 (s, ^{meta}C-$\underline{H}$, 2H), 1.48 (s, ^{ortho}C-C(C$\underline{H}_3$)$_3$, 18H), 1.27 (s, ^{para}C-C(C$\underline{H}_3$)$_3$, 9H).

^{11}B NMR (128 MHz, C_6D_6, 300 K) δ 61.0 (s, $\underline{B}$Cl$_2$).

^{13}C {^{1}H} NMR (126 MHz, C_6D_6, 300 K) δ 152.6 (s, $^{ortho}\underline{C}$- C(CH$_3$)$_3$), 151.6 (s, $^{para}\underline{C}$-C(CH$_3$)$_3$, 131.6* (s, $\underline{C}$-BCl$_2$), 122.4 (s, $^{meta}\underline{C}$-H), 38.2 (s, ^{ortho}C- $\underline{C}$(CH$_3$)$_3$), 35.0 (s, ^{para}C- $\underline{C}$(CH$_3$)$_3$), 34.0 (s, ^{ortho}C- C($\underline{C}$H$_3$)$_3$), 31.3 (s, ^{para}C- C($\underline{C}$H$_3$)$_3$).

* ^{ipso}*C*–B signal was observed by ^{1}H-^{13}C HMBC NMR experiment.

Mass Spectrum: HRMS (EI+) m/z calculated for $C_{18}H_{29}BCl_2$: 326.17339; Found: 326.17238.

2.6 Preparation of Mes*B(OH)$_2$

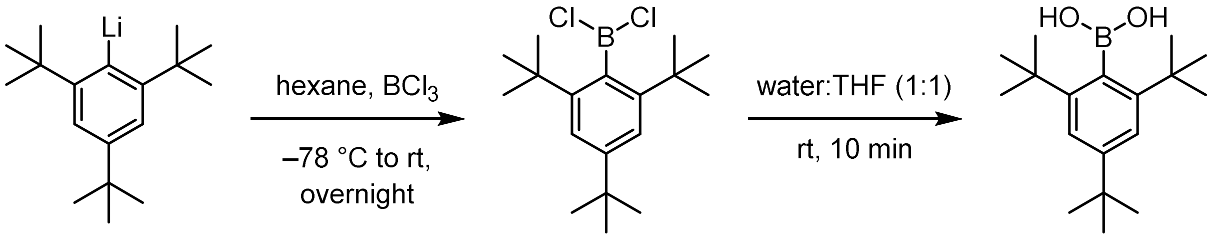

Step 1: BCl_3 in hexane (9.9 mL, C = 1M in hexane, 9.91 mmol, 2.50 equiv.) was added slowly over 20 minutes to a solution of Mes*Li (1.00 g, 3.96 mmol, 1.00 equiv.) in hexane (40 mL) at –78 °C, in a J. Young's ampoule. The solution was allowed to warm up to room temperature and stirred overnight. The solution was filtered and the residue was further extracted with pentane (3 x 5 mL). The solution was dried *in vacuo* affording a white solid. ^{1}H NMR analysis showed Mes*BCl_2 product with 92% purity with traces of Mes* as impurity (1.30 g of crude product, no yield was calculated). The product was used without any further purification.

Step 2: Water (25 mL) and THF (25 mL) were added successively to the crude Mes*BCl_2 (1.30 g) in a Schlenk tube. The solution was stirred vigorously for 10 min. The product was extracted with Et_2O (50 mL). The organic phase was separated, dried with Na_2SO_4, filtered and dried *in vacuo*. The resulting solid was washed with pentane (35 mL) affording the pure product Mes*B(OH)$_2$ as a white solid in 56% yield over the 2 steps (0.65 g, 2.23 mmol).

^{1}H NMR (500 MHz, $CDCl_3$, 300 K) δ 7.42 (s, ^{meta}C-*H*, 2H), 4.56 (s, BO*H*, 2H), 1.48 (s, ^{ortho}C-C(C*H*$_3$)$_3$, 18H), 1.33 (s, ^{para}C-C(C*H*$_3$)$_3$, 9H).

^{11}B NMR (160 MHz, $CDCl_3$, 300 K) δ 33.6 (s br, *B*OH).

^{13}C {^{1}H} NMR (126 MHz, $CDCl_3$, 300 K) δ 153.3 (s, ^{ortho}*C*-C(CH$_3$)$_3$), 150.10 (s, ^{para}*C*-C(CH$_3$)$_3$), 127.7* (^{ipso}*C*-B), 121.4 (s, ^{meta}*C*-H), 37.6 (s, ^{ortho}C-*C*(CH$_3$)$_3$), 35.1 (s, ^{para}C-*C*(CH$_3$)$_3$), 33.1 (s, ^{ortho}C-C(*C*H$_3$)$_3$), 31.5 (s, ^{para}C-C(*C*H$_3$)$_3$).

* ^{ipso}*C*–B signal was observed by ^{1}H-^{13}C HMBC NMR experiment.

Mass Spectrum: HRMS (EI+) m/z calculated for $C_{13}H_{31}BO_2$: 290.24116; Found: 290.24190.

2.7 **Preparation of 6, Mes*BCl(OTMS)**

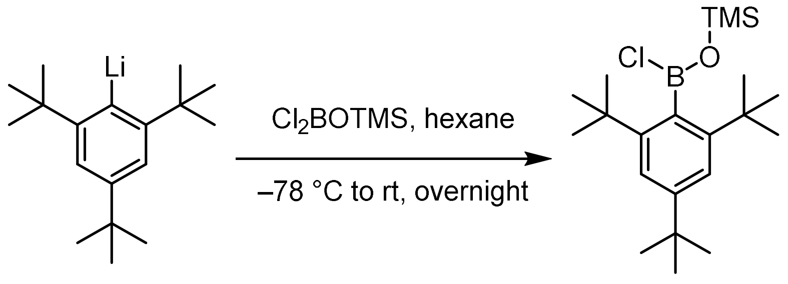

Cl_2BOTMS (3.25 mL, C = 1.29 M in hexane, 4.16 mmol, 1.05 equiv.) was added dropwise to a solution of Mes*Li (0.50 g, 1.98 mmol, 1.00 equiv.) in hexane (20 mL) at –78 °C. The solution was allowed to warm up to room temperature and stirred overnight. The solution was filtered and the residue extracted with pentane (1 x 5 mL). Volatiles were removed *in vacuo* and further heating under vacuum (70 °C, 2-3 x 10^{-2} mbar, 1 hour) gave a colourless oily residue which was predominantly compound **6** in ca. 48% yield (0.72 g, 1.90 mmol).*

* Mes*BCl(OTMS) could not be fully purified. Mes*BCl(OTMS) is a thick oil that solidified overtime, however its extreme solubility even in apolar solvents (e.g. pentane, TMS-O-TMS) precluded purification through fractional crystallization. Distillation, sublimation or extractions were unsuccessful. Mes*BCl(OTMS) therefore was used as obtained from this procedure with ~90% purity by ^{1}H NMR integration.

^{1}H NMR (500 MHz, C_6D_6, 300 K) δ 7.53 (s, ^{meta}C-*H*, 2H), 1.55 (s, ^{ortho}C-C(C*H*$_3$)$_3$, 18H), 1.33 (s, ^{para}C-C(C*H*$_3$)$_3$, 9H), 0.31 (s, Si(C*H*$_3$)$_3$, 9H).

^{11}B NMR (160 MHz, C_6D_6, 300 K) δ 39.5 (s br, *B*Cl(OTMS)).

^{13}C {^{1}H} NMR (126 MHz, C_6D_6, 300 K) δ 152.1 (s, ^{ortho}*C*-C(CH$_3$)$_3$), 150.1 (s, ^{para}*C*-C(CH$_3$)$_3$), 131.4* (^{ipso}*C*-B), 121.7 (s, ^{meta}*C*-H), 38.0 (s, ^{ortho}C-*C*(CH$_3$)$_3$), 35.0 (s, ^{para}C-*C*(CH$_3$)$_3$), 33.5 (s, ^{ortho}C-C(*C*H$_3$)$_3$), 31.5 (s, ^{para}C-C(*C*H$_3$)$_3$), 0.60 (s, Si(*C*H$_3$)$_3$).

^{29}Si NMR (99 MHz, C_6D_6, 300 K) δ 18.1 (s, *Si*(CH$_3$)$_3$).

* ^{ipso}*C*–B signal was observed by ^{1}H-^{13}C HMBC NMR experiment.

Mass Spectrum: HRMS (EI+) m/z calculated for $C_{21}H_{38}BClOSi$: 380.24680; Found: 380.24527.

2.8 **Preparation of 7, Mes*BO-AlCl$_3$**

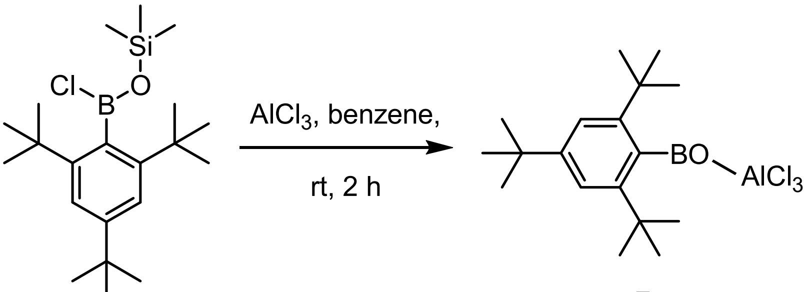

7

Mes*B(Cl)OTms (0.30 g, 0.79 mmol, 1.00 equiv.) in benzene (10 mL) was added over 5 minutes to a solution of AlCl$_3$ (0.13 g, 0.98 mmol, 1.20 equiv.) in benzene (20 mL) at room temperature in a J. Young's ampoule. The solution was stirred at room temperature for 2 hours. The solution was filtered and volatiles were removed *in vacuo*. The brown residue was washed with pentane (10 mL). Impurities were sublimed from the crude product under vacuum at high temperature (115 °C, 1-5 x 10^{-2} mbar, 1 hour) affording **7** as a pale brown solid in 37% yield (0.12 g, 0.30 mmol).

^{1}H NMR (500 MHz, C_6D_6, 300 K) δ 7.23 (s, ^{meta}C*H*, 2H), 1.20 (s, ^{ortho}C-C(C*H$_3$*)$_3$, 18H), 1.05 (s, ^{para}C-C(C*H$_3$*)$_3$, 9H).

^{11}B NMR (160 MHz, C_6D_6, 300 K) δ 28.0 (br s).

^{11}B {^{1}H} NMR (160 MHz, C_6D_6, 300 K) δ 27.5 (br s).

^{13}C {^{1}H} NMR (126 MHz, C_6D_6, 300 K) δ 166.9 (s, ^{ortho}*C*-C(CH$_3$)$_3$), 161.8 (s, ^{para}*C*-C(CH$_3$)$_3$), 121.5 (s, ^{meta}*C*H), 97.4* (^{ipso}*C*-B), 36.8 (s, ^{ortho}C-*C*(CH$_3$)$_3$), 36.0 (s, ^{para}C-*C*(CH$_3$)$_3$), 32.6 (s, ^{para}C-C(*C*H$_3$)$_3$), 30.6 (s, ^{ortho}C-C(*C*H$_3$)$_3$).

^{27}Al NMR (130 MHz, C_6D_6, 300 K) δ 95.2 (br s).

* ^{ipso}*C*–B signal was observed by ^{1}H-^{13}C HMBC NMR experiment.

Mass Spectrum: HRMS (EI+) m/z calculated for $C_{18}H_{29}BOAlCl_3$: 404.11869; Found: 404.11949.

FTIR: (vmax (neat)/cm^{-1}) 1830 (v^{11}BO).

IR: (vmax (benzene)/cm^{-1}) 1843 (v^{11}BO).

2.9 **Preparation of 8, Mes*(DMAP)BO-AlCl$_3$**

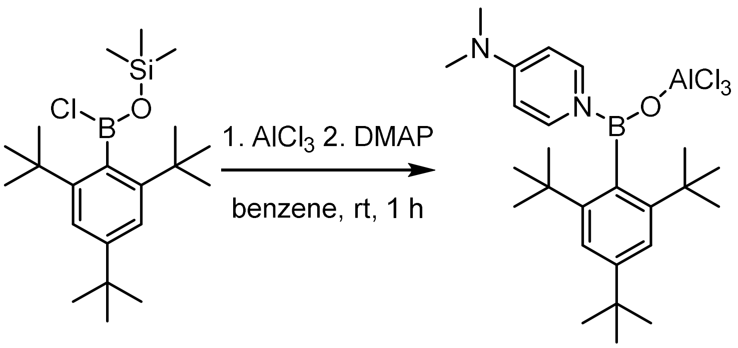

8

Mes*BCl(OTMS) (0.30 g, 0.79 mmol, 1.00 equiv.) and AlCl$_3$ (0.13 g, 0.98 mmol, 1.20 equiv.) were dissolved in benzene (10 mL) at room temperature in a J. Young's ampoule and stirred for 0.5 hour at room temperature. 4-DMAP (0.1 g, 0.79 mmol, 1.00 equiv.) was added to the solution in one portion as a solid. The resulting solution was stirred for 0.5 hour at room temperature before being filtered. Volatiles were removed *in vacuo* giving a pale yellow waxy solid, which was redissolved in PhCl (1 mL) and layered with pentane (2 mL). Colourless crystals grew overtime and were isolated by filtration after 3 days at room temperature. Drying *in vacuo* afforded compound **8** as colourless crystalline solid in 44% yield (0.18 g, 0.34 mmol).*

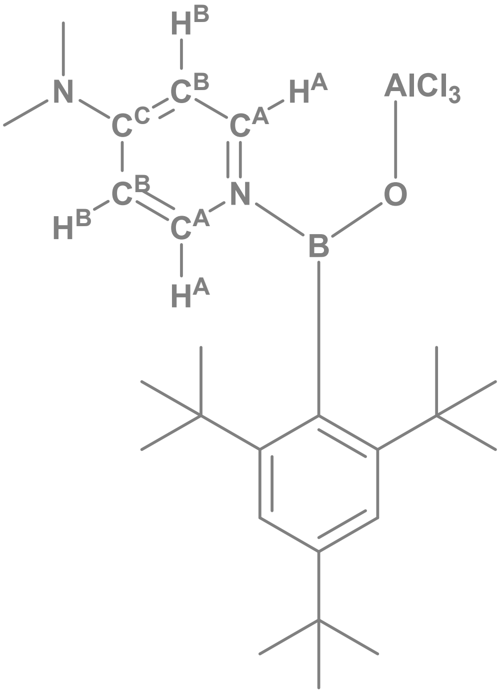

Figure S1: Scheme of compound **8**.

^{1}H NMR (500 MHz, C_6D_6, 300 K) δ 9.08 (br d, $^{3}J_{HH}$ = 7.6 Hz, $\underline{H}^{A}$, 1H,), 7.66 (s, ^{meta}C$\underline{H}$, 2H), 7.06 (dd, $^{3}J_{HH}$ = 7.6 Hz, $^{4}J_{HH}$ = 1.3 Hz, $\underline{H}^{A}$, 1H), 5.72 (dd, $^{3}J_{HH}$ = 7.7 Hz, $^{4}J_{HH}$ = 2.9 Hz, $\underline{H}^{B}$, 1H), 4.89 (dd, $^{3}J_{HH}$ = 7.6 Hz, $^{4}J_{HH}$ = 2.9 Hz, $\underline{H}^{B}$, 1H), 2.19 (br s, N(C$\underline{H}_3$), 3H), 2.01(br s, N(C$\underline{H}_3$), 3H), 1.51 (s, ^{ortho}C-C(C$\underline{H}_3$)$_3$, 18H), 1.38 (s, ^{para}C-C(C$\underline{H}_3$)$_3$, 9H).

^{11}B NMR (160 MHz, C_6D_6, 300 K) δ 32.6 (br s).

^{11}B {^{1}H} NMR (160 MHz, C_6D_6, 300 K) δ 31.9 (br s).

^{13}C {^{1}H} NMR (126 MHz, C_6D_6, 300 K) δ 157.2 (s, $\underline{C}^{C}$), 154.1 (s, $^{ortho}\underline{C}$-C(CH$_3$)$_3$), 151.1 (s, $^{para}\underline{C}$-C(CH$_3$)$_3$), 144.2 (s, $\underline{C}^{A}$), 141.2 (s, $\underline{C}^{A}$), 126.0** ($^{ipso}\underline{C}$-B), 122.4 (s, $^{meta}\underline{C}$H), 107.6 (s, $\underline{C}^{B}$), 105.8 (s, $\underline{C}^{B}$), 39.6 (s, N($\underline{C}$H$_3$)), 39.2 (s, N($\underline{C}$H$_3$)), 38.4 (s, ^{ortho}C-$\underline{C}$(CH$_3$)$_3$), 35.2 (s, ^{para}C-$\underline{C}$(CH$_3$)$_3$), 33.6 (s, ^{ortho}C-C($\underline{C}$H$_3$)$_3$), 31.5 (s, ^{para}C-C($\underline{C}$H$_3$)$_3$).

^{27}Al NMR (130 MHz, C_6D_6, 300 K) δ 90.5 (br s).***

* Residual PhCl was observed by ^{1}H-NMR and ^{13}C-NMR.

** ^{ipso}C-B signal was observed by ^{1}H-^{13}C HMBC NMR experiment.

*** Compound **8** showed slow overtime decomposition. Alongside the trace of unknown decomposition species observed in the ^{1}H NMR, signal from $AlCl_4^-$ was observed in the ^{27}Al NMR.

Mass Spectrum: HRMS (EI+) m/z calculated for $C_{25}H_{39}N_2BOAlCl_3$: 526.20419; Found: 526.20627.

FTIR: (vmax (neat)/cm^{-1}) 1639 (v^{11}BO).

IR: (vmax (benzene)/cm^{-1}) 1641 (v^{11}BO).

2.10 Preparation of 9

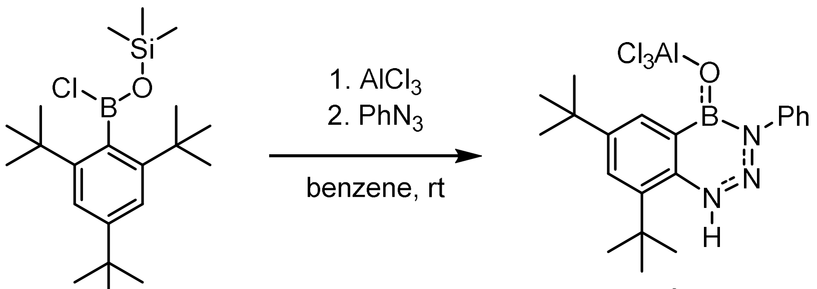

Mes*B(Cl)OTMS, **6** (0.18 g, 0.48 mmol, 1.00 equiv.) and $AlCl_3$ (0.08 g, 0.58 mmol, 1.20 equiv.) were dissolved in benzene (10 mL) in a J. Young's ampoule at room temperature. After stirring for 30 minutes, PhN_3 (53 µL, 0.48 mmol, 1.00 equiv.) was added dropwise at room temperature. The solution was stirred for an additional 30 minutes before being filtered. Volatiles were removed from the filtrate *in vacuo* giving an oily residue. The residue was washed with pentane (5 mL). Redissolving the residue in benzene (3 mL) afforded a biphasic mixture, the top layer was removed. The bottom layer was dissolved in PhCl (0.7 mL), the solution was stored in the freezer for 2 hours, allowing precipitation of a pale-yellow solid. The pale-yellow solid was isolated by filtration and dried *in vacuo*. NMR analysis revealed a mixture of nearly 1:1 of PhCl:**9**. Dissolving the solid in DCM and drying *in vacuo* allowed removal of PhCl, affording **9** as a pale-yellow solid in 26% yield (0.06 g, 0.13 mmol).

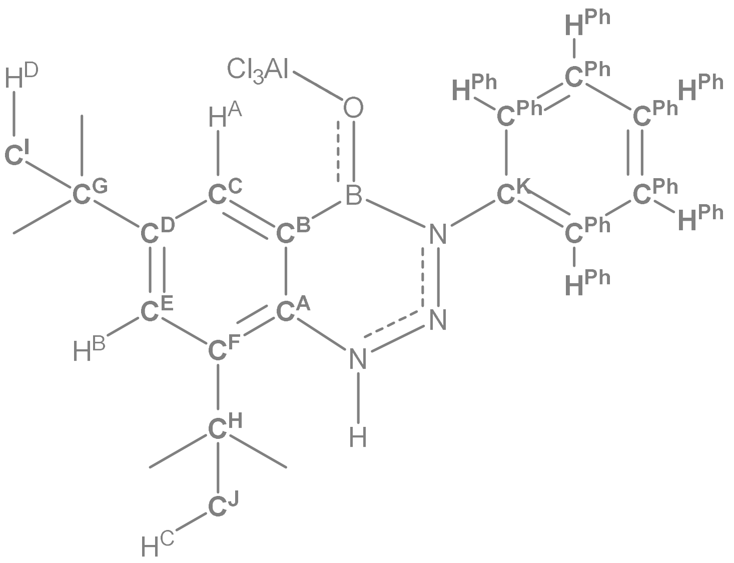

Figure S2: Scheme of compound **9**.

^{1}H NMR (500 MHz, CD_2Cl_2, 300 K) δ 12.54 (s, br N*H*, 1H), 8.65 (d, $^{4}J_{HH}$ = 2.0 Hz, $\underline{H}^{A}$, 1H), 8.04 (d, $^{4}J_{HH}$ = 2.1 Hz, $\underline{H}^{B}$, 1H), 7.75-7.70 (m, $\underline{H}^{Ph}$, 2H), 7.65-7.57 (m, $\underline{H}^{Ph}$, 3H), 1.70 (s, $\underline{H}^{C}$, 9H), 1.46 (s, $\underline{H}^{D}$, 9H).

^{11}B NMR (160 MHz, CD_2Cl_2, 300 K) δ 24.6 (s br, C-*B*(O)N).

^{13}C {^{1}H} NMR (126 MHz, CD_2Cl_2, 300 K) δ 157.5 (s, $\underline{C}^{D}$), 140.8 (s, $\underline{C}^{K}$), 139.0 (s, $\underline{C}^{F}$), 138.4 (s, $\underline{C}^{A}$), 131.2 (s, $\underline{C}^{E}$), 130.6 (s, $\underline{C}^{Ph}$), 130.0 (s, $\underline{C}^{C}$), 129.9 (s, $\underline{C}^{Ph}$), 126.1 (br s, $\underline{C}^{B}$),* 125.4 (s, $\underline{C}^{Ph}$).

^{27}Al NMR (130 MHz, CD_2Cl_2, 300 K) δ 89.3 (br s).**

* $\underline{C}^{B}$–B signal was observed by ^{1}H-^{13}C HMBC NMR experiment.

** Minor residual signal from $AlCl_4^-$ was observed by ^{27}Al NMR coming from trace decomposition.

*** Alongside the signals from **9**, signals from a minor isomer **9B** were observed by ^{1}H and ^{13}C NMR spectroscopy and will be discussed in detail later (see Part 4.3).

Despite repeated attempts compound **9** proved unsuitable for analysis by mass spectrometry using EI and ESI. However, during the ESI attempts a peak corresponding to a product from partial hydrolysis was found, specifically replacement of the $AlCl_3$ unit with H^+: $C_{20}H_{26}AlBCl_3N_3O$ – $AlCl_3$ + H^+ → $C_{20}H_{27}BN_3O$.

Mass Spectrum: HRMS (ESI+) m/z calculated for calculated $C_{20}H_{27}BN_3O$: 336.2242; Found: 336.2245.

2.11 **NMR spectra of isolated compounds**

2.11.1 NMR spectra of Cl_2BOTMS

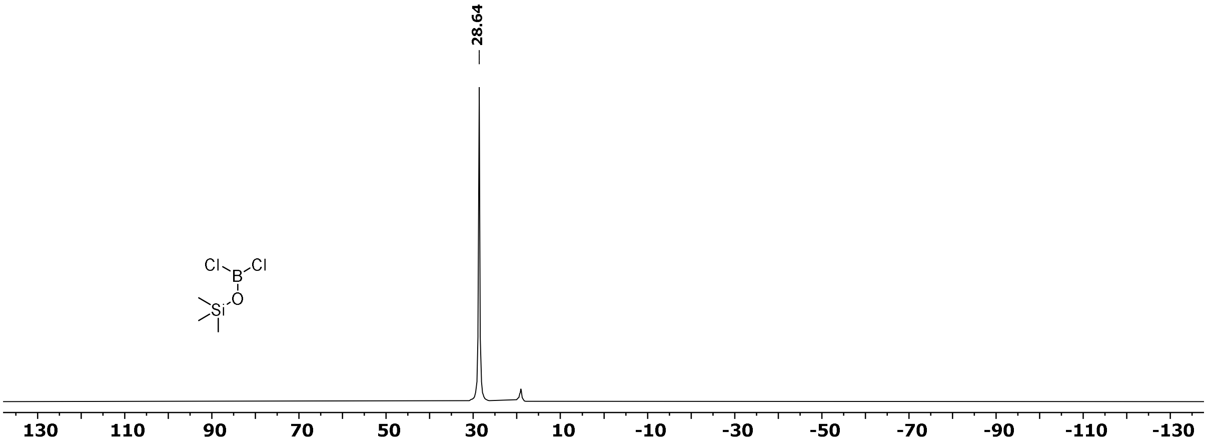

Figure S3: ^{11}B NMR spectrum (160 MHz, hexane, 300 K) of Cl_2BOTMS. Residual $BOTMS_3$ is observed at 19.1 ppm.

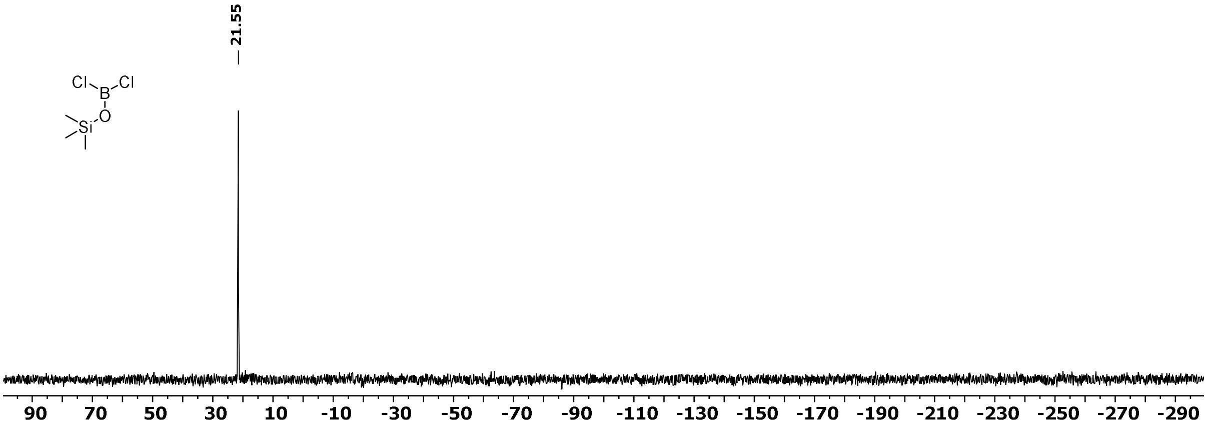

Figure S4: ^{29}Si NMR spectrum (99 MHz, hexane, 300 K) of Cl_2BOTMS.

*2.11.2 NMR spectra of Mes*BCl₂*

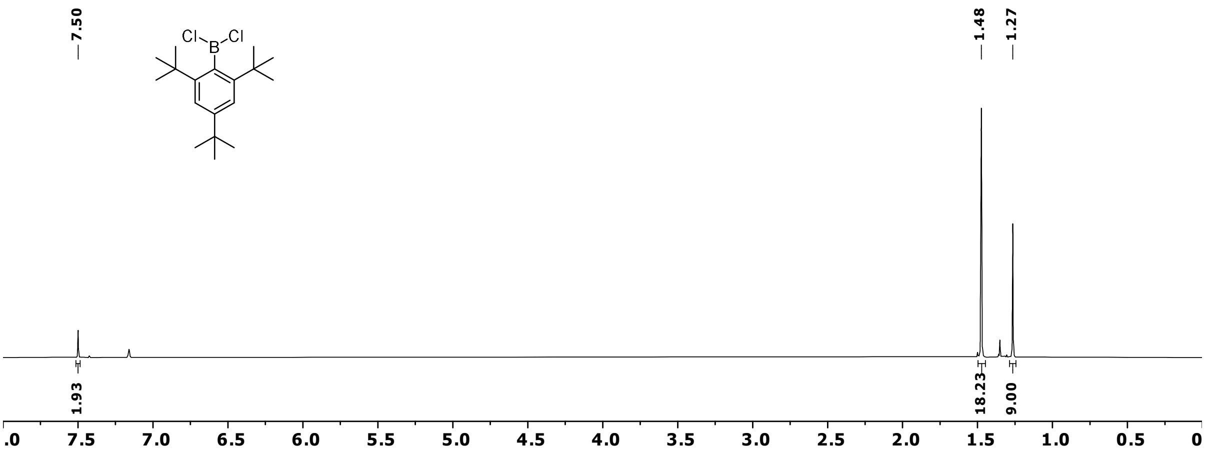

Figure S5: ^{1}H NMR spectrum (400 MHz, C_6D_6, 300 K) of Mes*BCl_2.

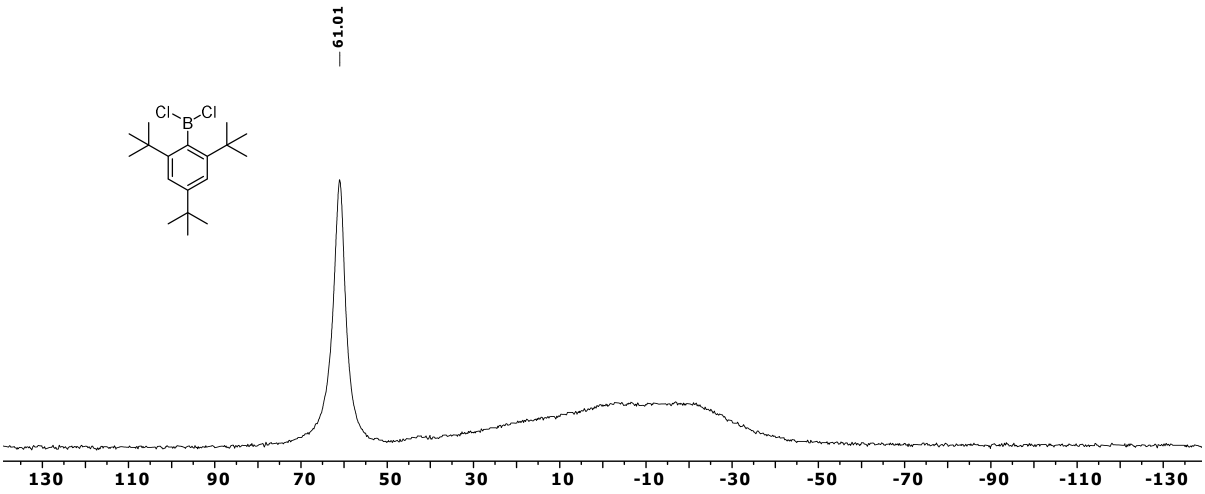

Figure S6: ^{11}B NMR spectrum (128 MHz, C_6D_6, 300 K) of Mes*BCl_2.

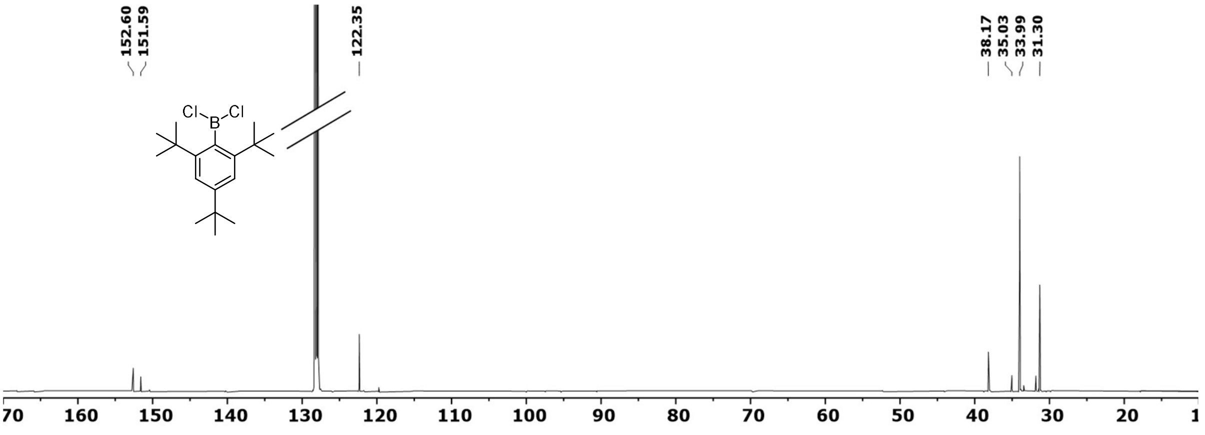

Figure S7: ^{13}C {^{1}H} NMR spectrum (126 MHz, C_6D_6, 300 K) of Mes*BCl_2.

*2.11.3 NMR spectra of Mes*B(OH)₂*

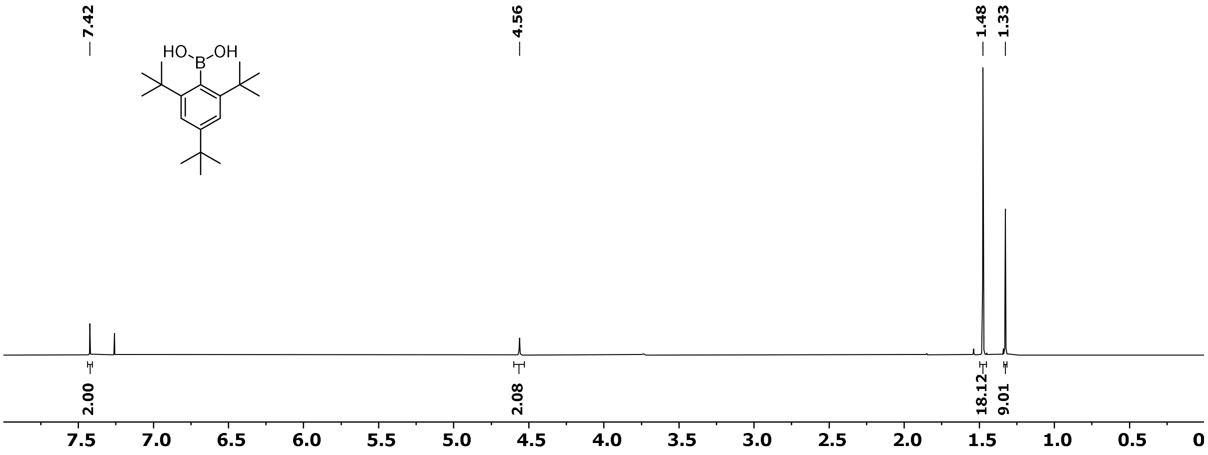

Figure S8: ^{1}H NMR spectrum (500 MHz, $CDCl_3$, 300 K) of Mes*B(OH)$_2$.

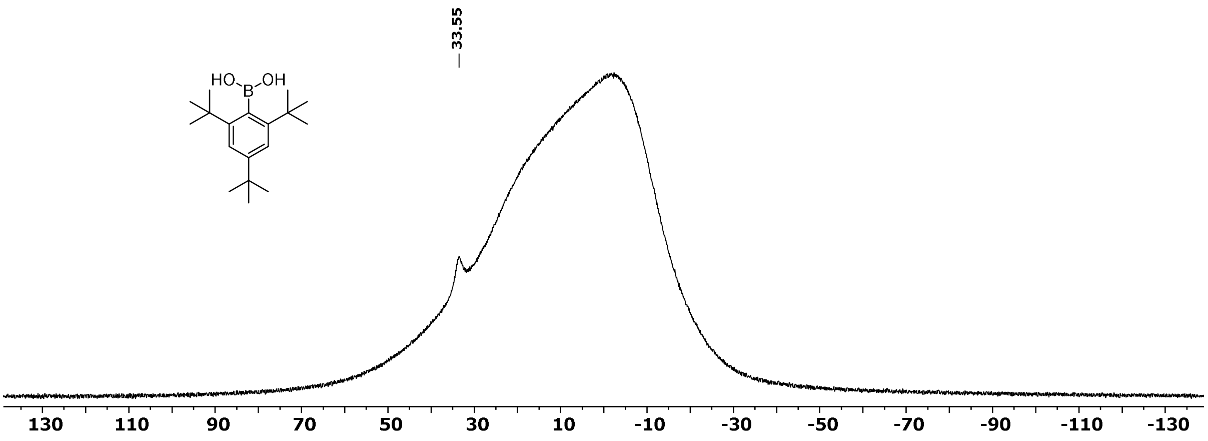

Figure S9: ^{11}B NMR spectrum (160 MHz, $CDCl_3$, 300 K) of Mes*B(OH)$_2$.

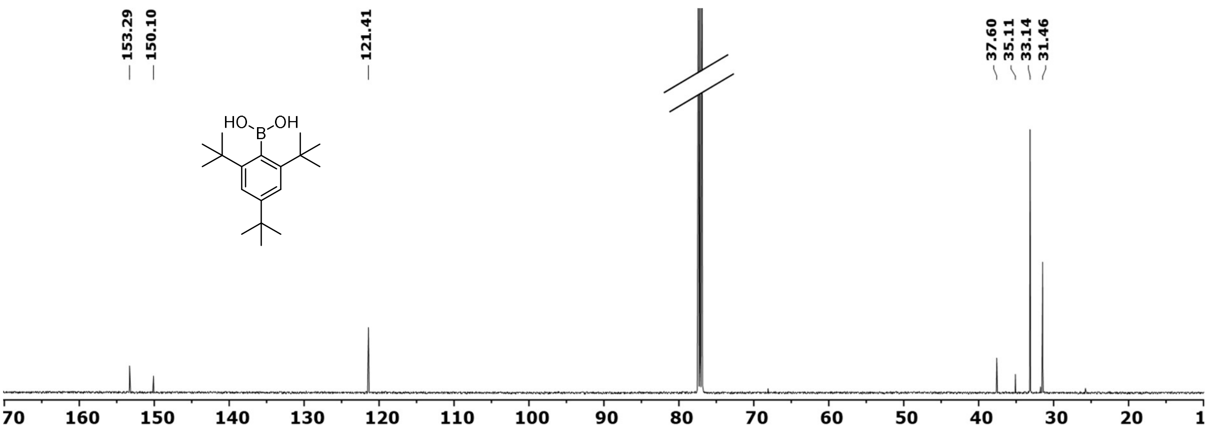

Figure S10: ^{13}C {^{1}H} NMR spectrum (126 MHz, $CDCl_3$, 300 K) of Mes*B(OH)$_2$.

*2.11.4 NMR spectra of 6, Mes*BCl(OTMS)*

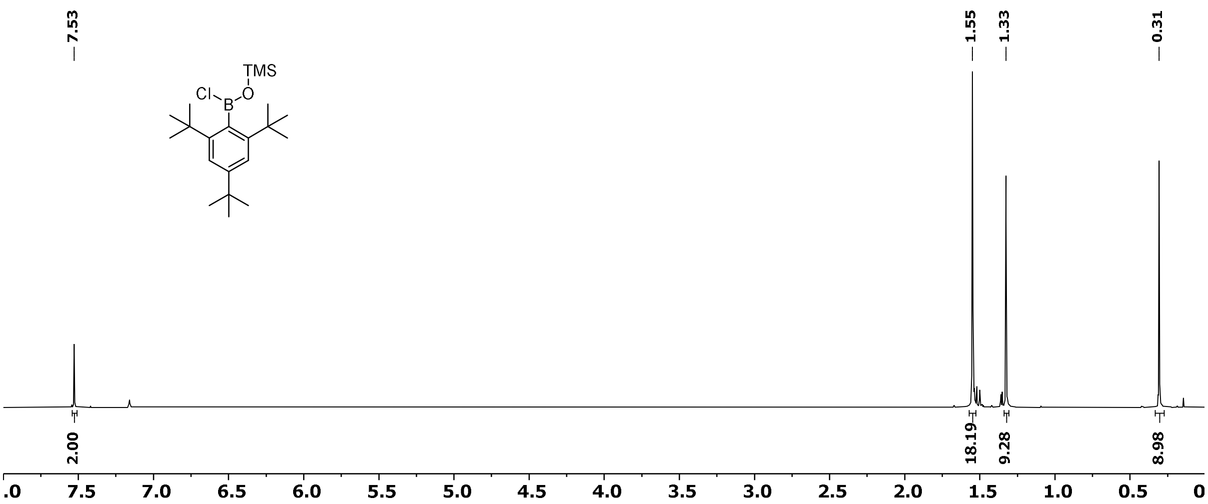

Figure S11: ^{1}H NMR spectrum (500 MHz, C_6D_6, 300 K) of **6**.

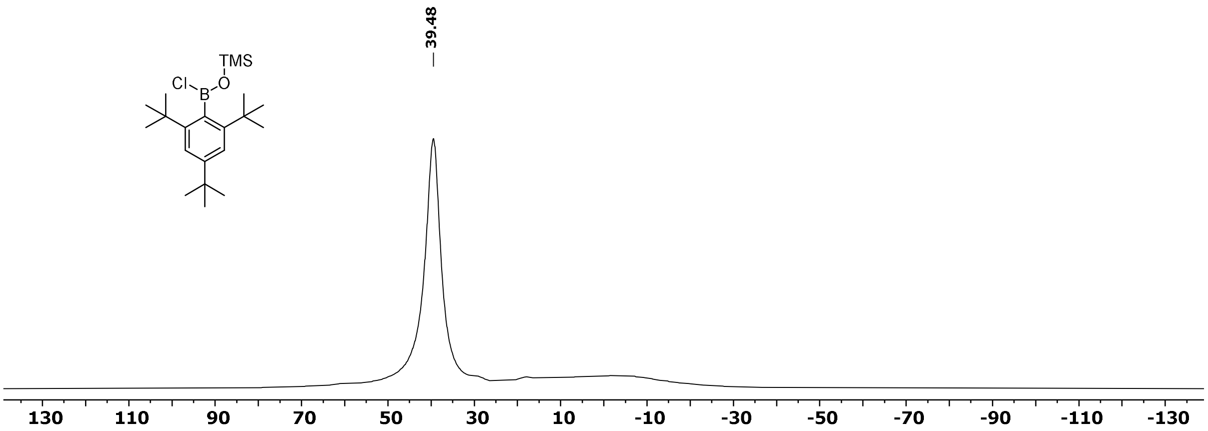

Figure S12: ^{11}B NMR spectrum (160 MHz, C_6D_6, 300 K) of **6**.

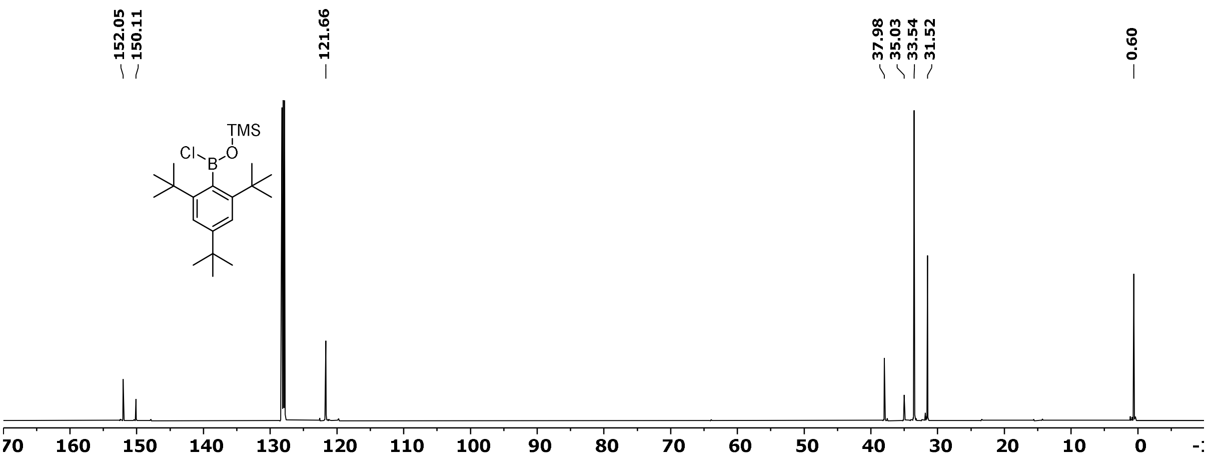

Figure S13: ^{13}C {^{1}H} NMR spectrum (126 MHz, C_6D_6, 300 K) of **6**.

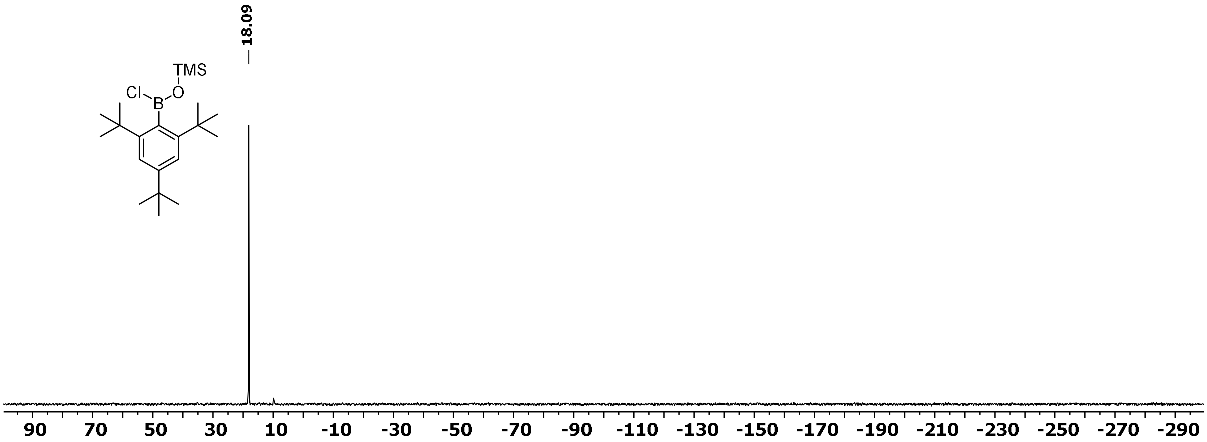

Figure S14: ^{29}Si NMR spectrum (99 MHz, C_6D_6, 300 K) of **6**.

*2.11.5 NMR spectra of 7, Mes*BO-AlCl$_3$*

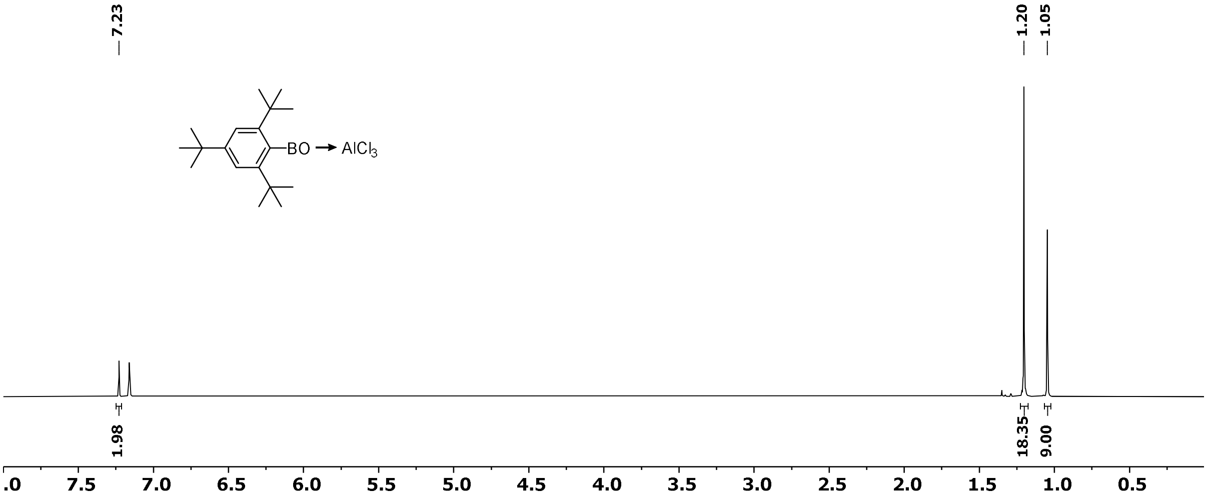

Figure S15: ^{1}H NMR spectrum (500 MHz, C_6D_6, 300 K) of **7**.

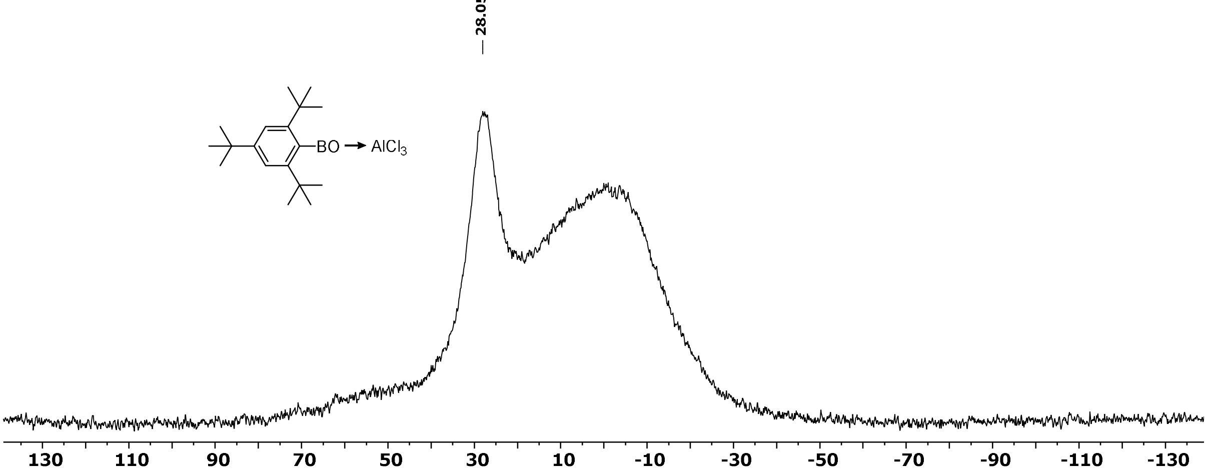

Figure S16: ^{11}B NMR spectrum (160 MHz, C_6D_6, 300 K) of **7**.

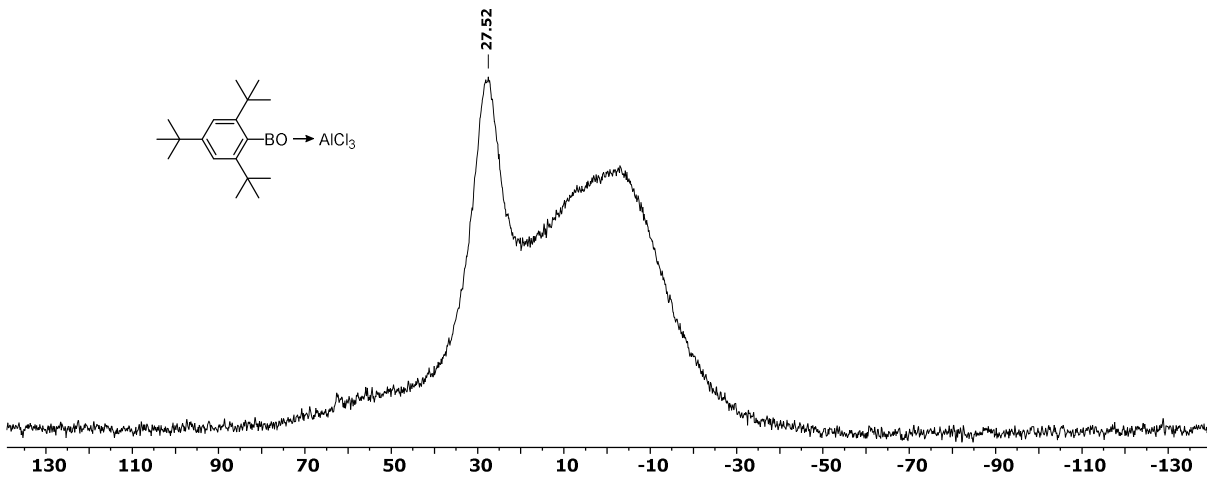

Figure S17: $^{11}B\{^{1}H\}$ NMR spectrum (160 MHz, C_6D_6, 300 K) of **7**.

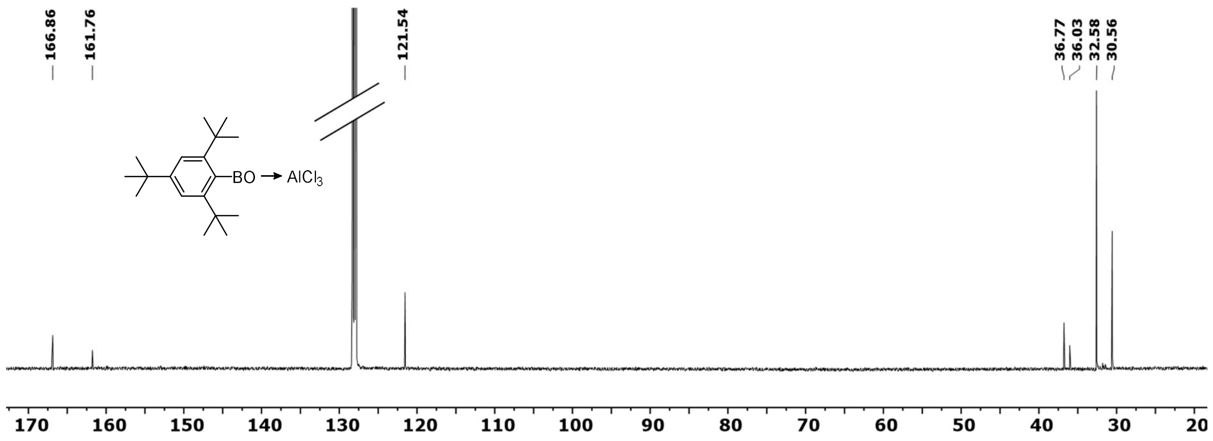

Figure S18: $^{13}C\{^{1}H\}$ NMR spectrum (126 MHz, C_6D_6, 300 K) of **7**.

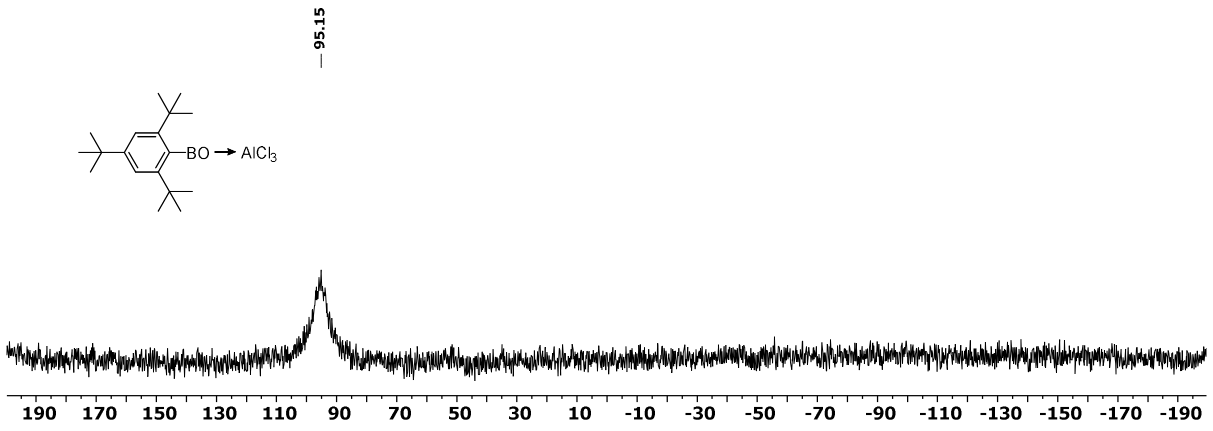

Figure S19: ^{27}Al NMR spectrum (130 MHz, C_6D_6, 300 K) of **7**.

2.11.6 NMR spectra of 8, Mes(DMAP)BO-AlCl$_3$*

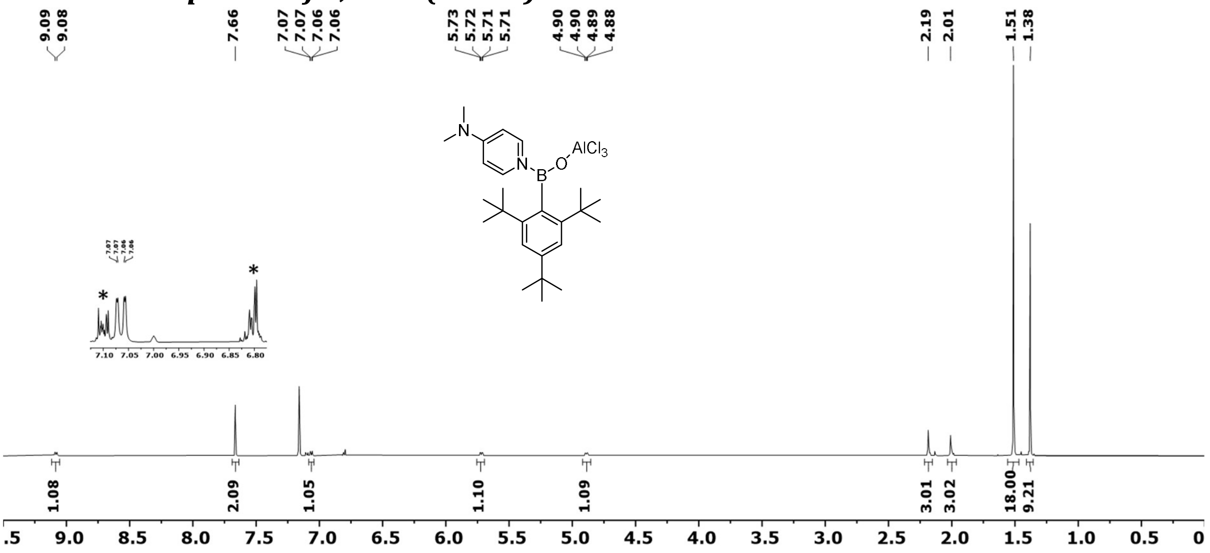

Figure S20: ^{1}H NMR spectrum (500 MHz, C_6D_6, 300 K) of **8**. * Signals from PhCl were observed by ^{1}H NMR alongside **8**.

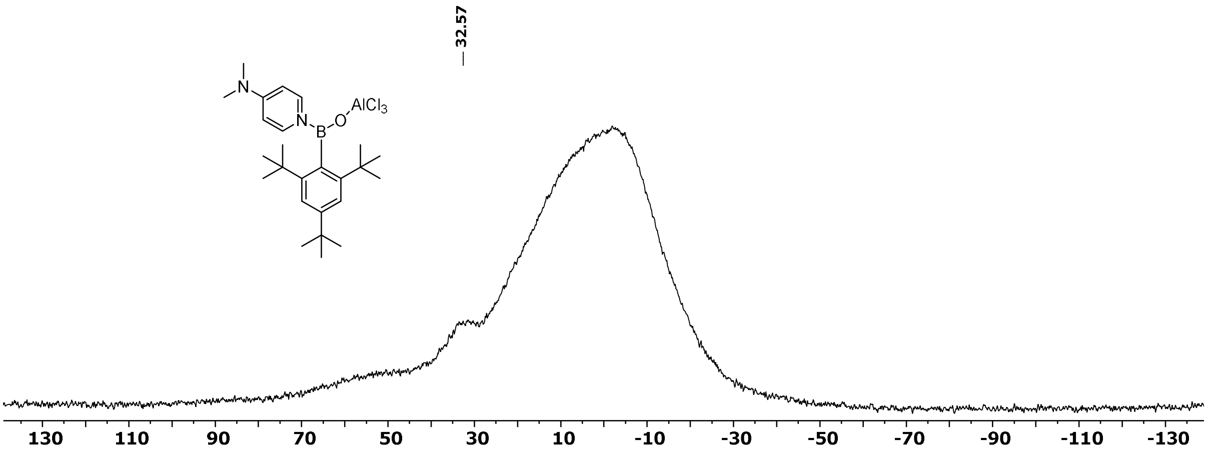

Figure S21: ^{11}B NMR spectrum (160 MHz, C_6D_6, 300 K) of **8**.

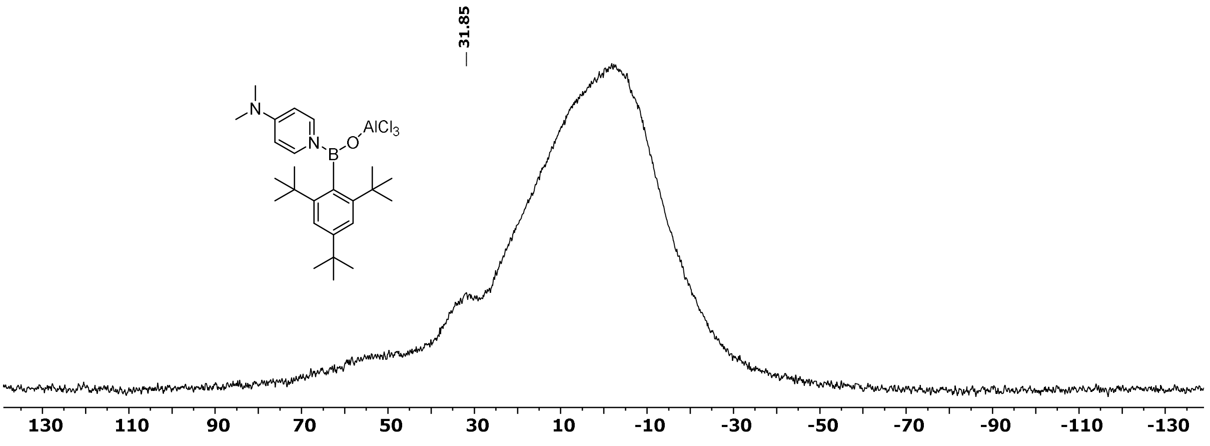

Figure S22: ^{11}B {^{1}H} NMR spectrum (160 MHz, C_6D_6, 300 K) of **8**.

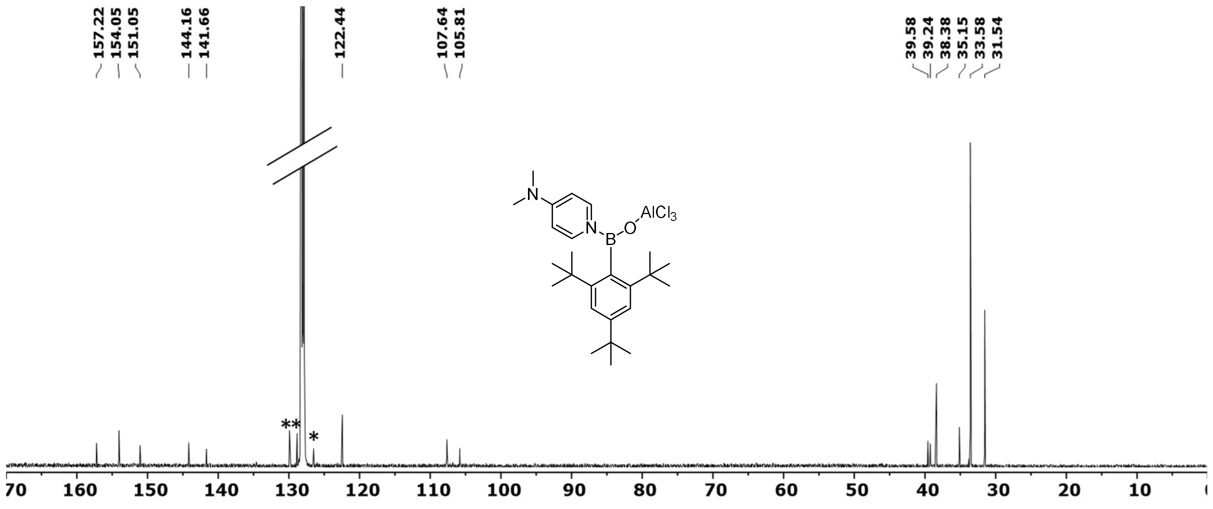

Figure S23: $^{13}C\ \{^{1}H\}$ NMR spectrum (126 MHz, C_6D_6, 300 K) of **8**. * Signals from PhCl were observed by ^{13}C NMR alongside the product.

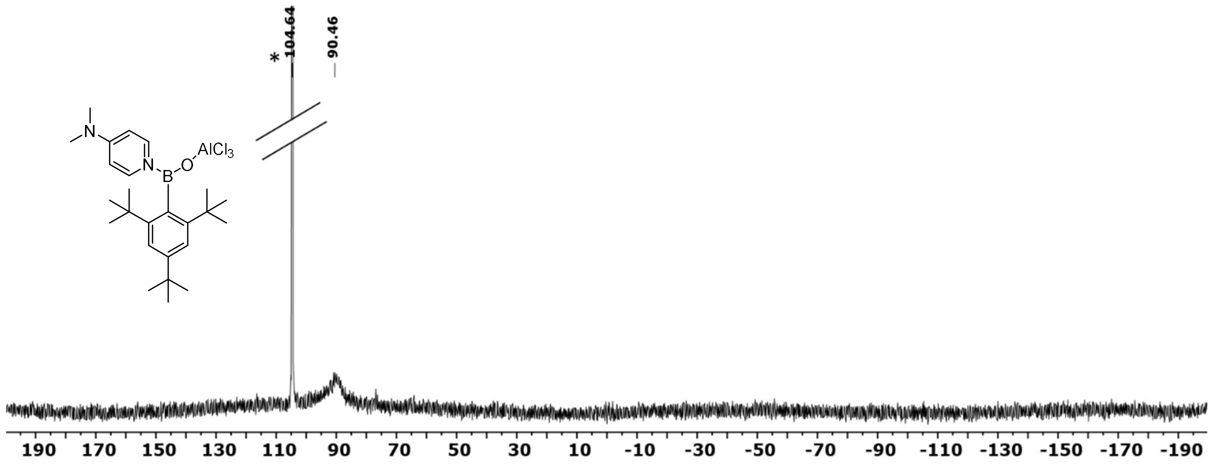

Figure S24: ^{27}Al NMR spectrum (130 MHz, C_6D_6, 300 K) of **8**. * Signal from $AlCl_4^-$ was observed from decomposition of **8** overtime in solution.

2.11.7 NMR spectra of 9

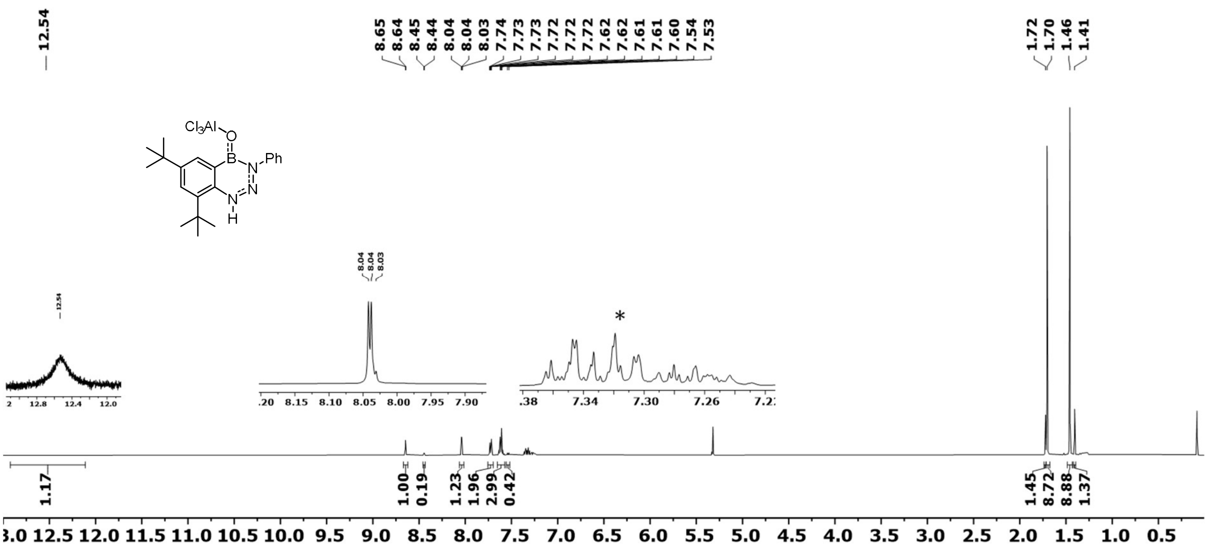

Figure S25: ^{1}H NMR spectrum (500 MHz, CD_2Cl_2, 300 K) of **9**. * Residual signals from PhCl overlapped were observed. Signals from a minor isomer **9B** were seen alongside **9** (ratio 1:0.19, see Part 4.3).

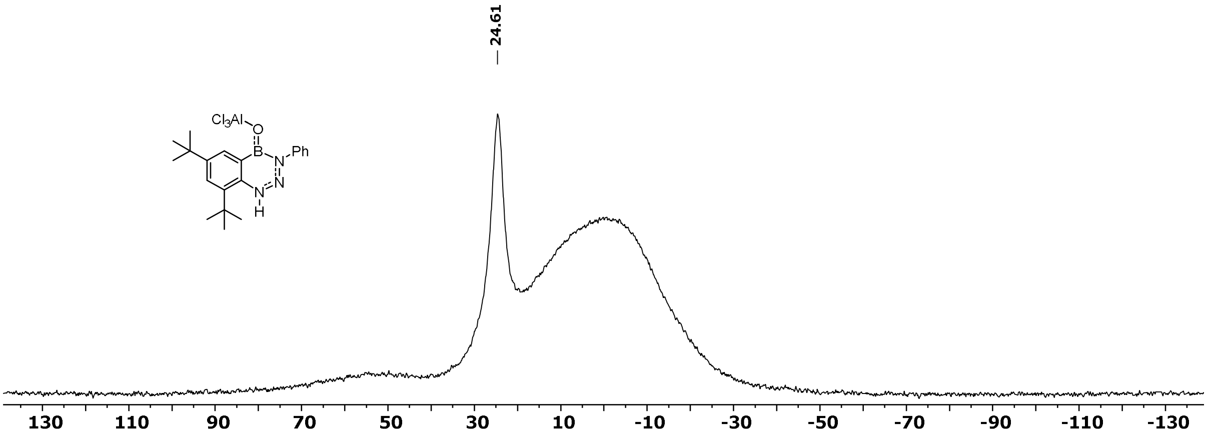

Figure S26: ^{11}B NMR spectrum (160 MHz, CD_2Cl_2, 300 K) of **9**.

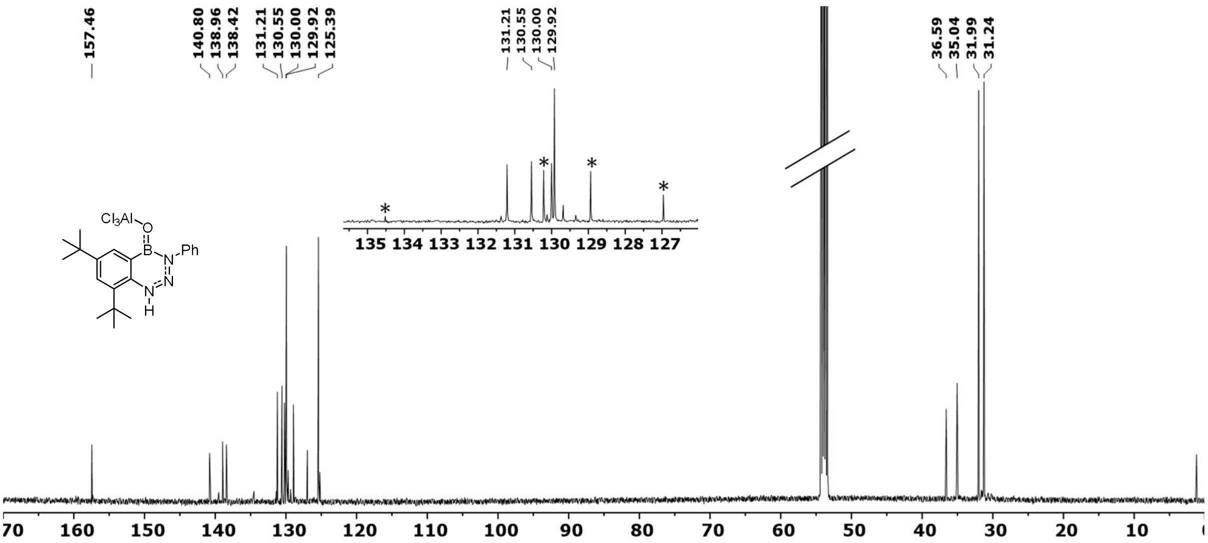

Figure S27: ^{13}C {^{1}H} NMR spectrum (126 MHz, CD_2Cl_2, 300 K) of **9**. * Residual signals from PhCl were observed alongside **9**. Signals from a minor isomer **9B** were seen alongside **9** (see Part 4.3).

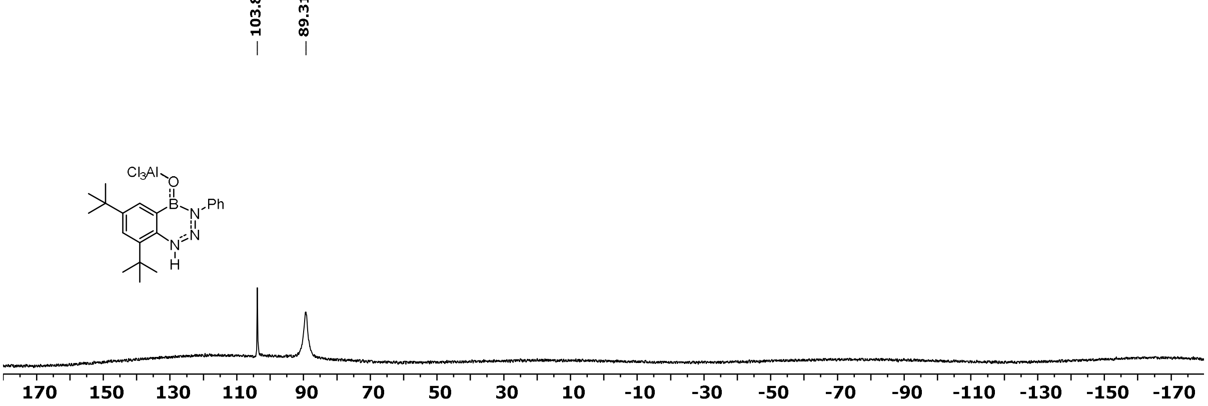

Figure S28: ^{27}Al NMR spectrum (130 MHz, CD_2Cl_2, 300 K) of **9**. Signal of $AlCl_4^-$ (δ 103.8) was observed alongside the product from decomposition of the product **9**.

2.12 **IR and FTIR spectra**

2.12.1 IR and FTIR spectra of 7, Mes*BO-AlCl$_3$

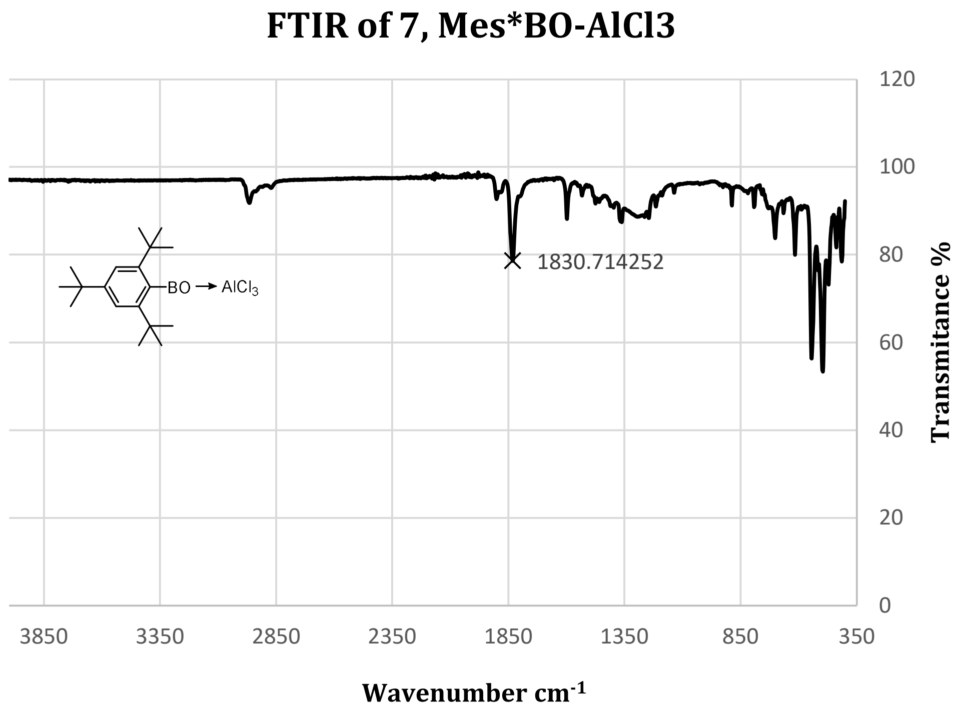

Figure S29: FTIR spectrum of **7**.

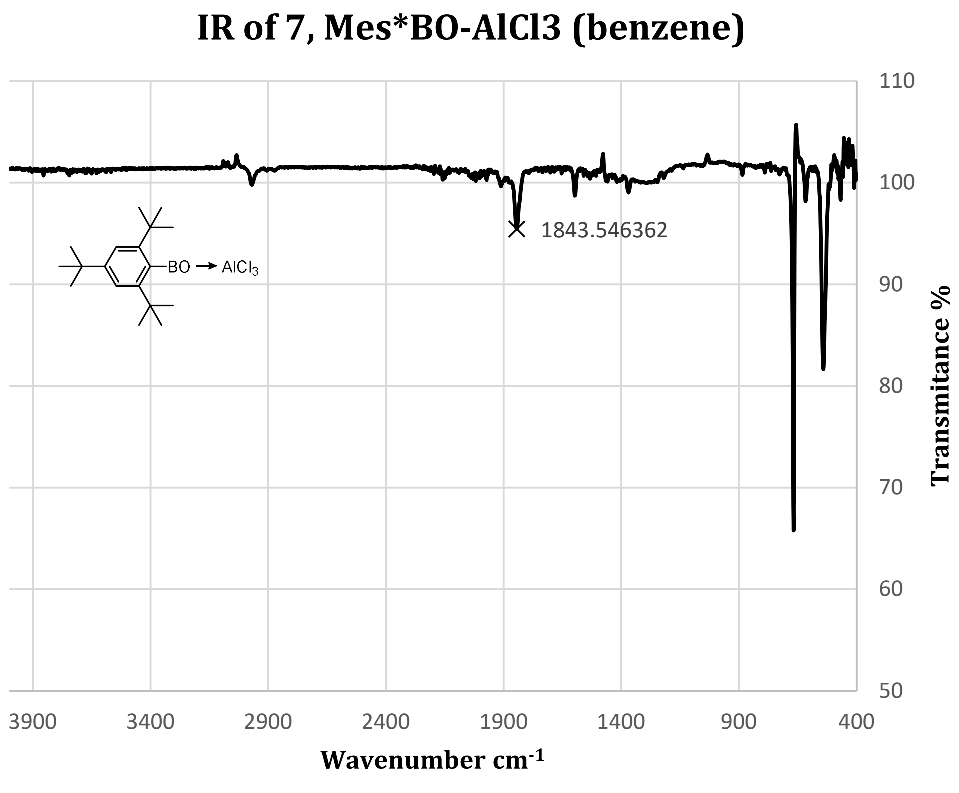

Figure S30: IR spectrum of **7** in benzene.

2.12.2 IR and FTIR spectra of 8, Mes(DMAP)BO-AlCl$_3$*

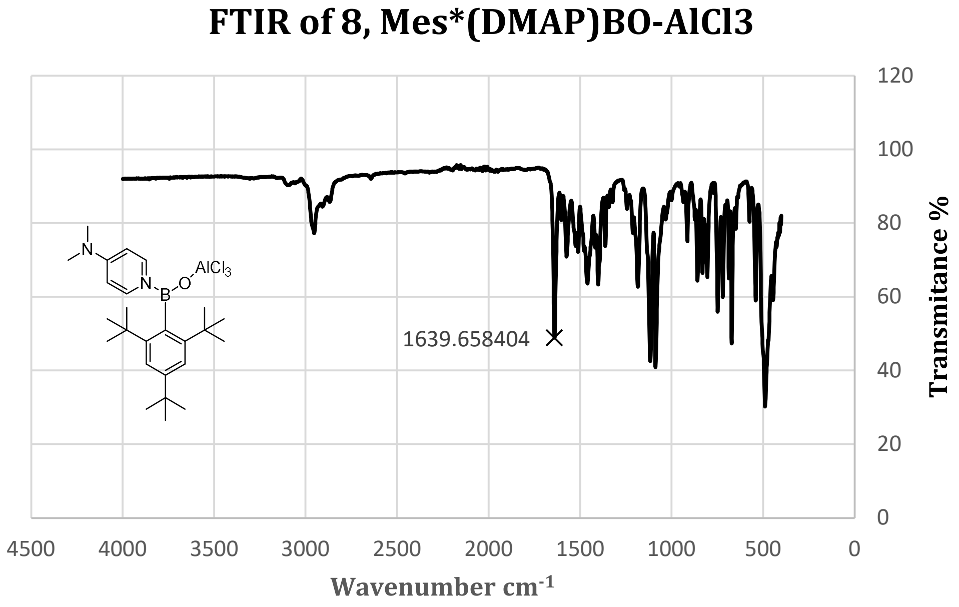

Figure S31: FTIR spectrum of **8**.

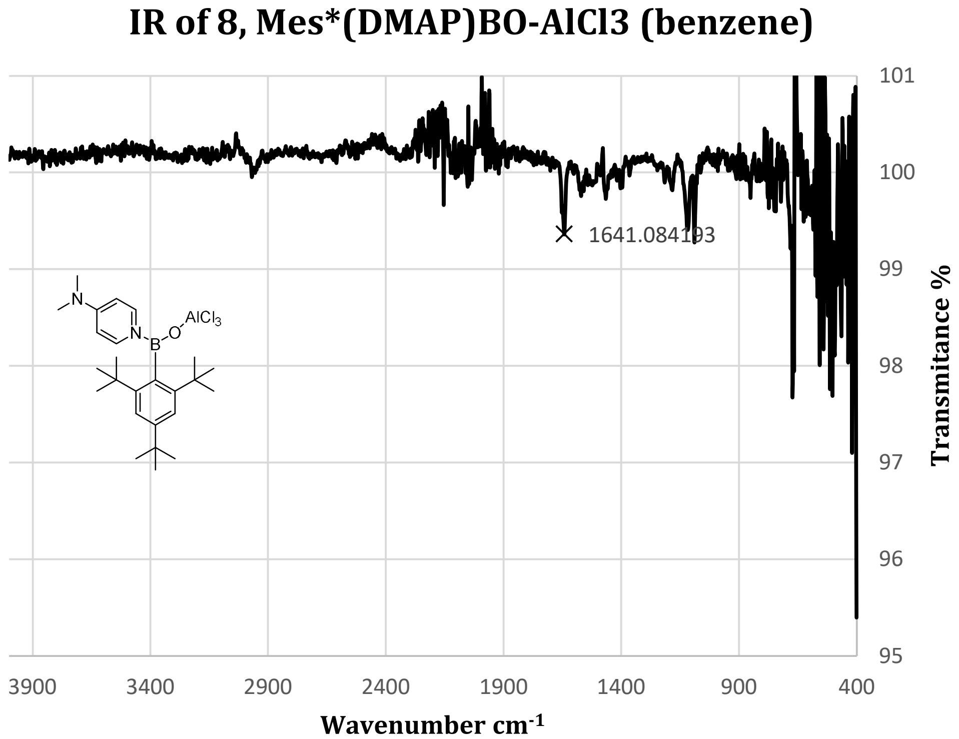

Figure S32: IR spectrum of **8** in benzene. The low solubility of **8** in benzene resulted in weak intensity of the signals from **8**.

3 Preliminary studies

3.1 Attempts of synthesis of 1_2, $[Mes^*BO]_2$, from $Mes^*B(OMe)_2$

3.1.1 Attempt of synthesis of 1_2, $[Mes^*BO]_2$, from reported route

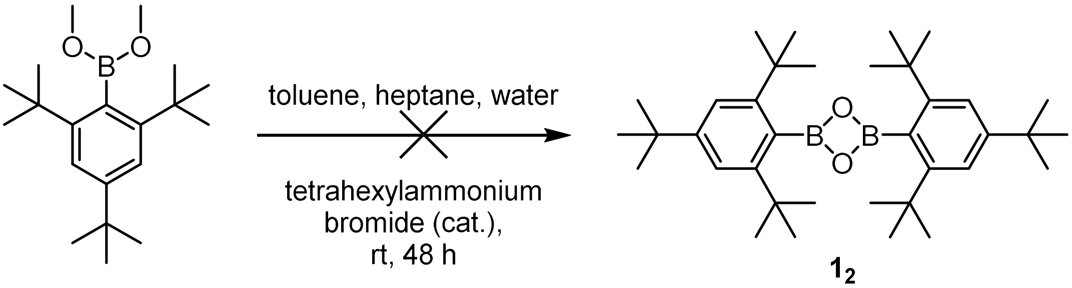

This preparation was based on a literature procedure.[S6]

$Mes^*B(OMe)_2$ (0.27 g, 0.85 mmol, 1 equiv.) and tetrahexylammonium bromide (cat., 1 mg, 0.003 mmol) were dissolved in a mixture of toluene (2.6 mL), heptane (2.6 mL) and water (5.2 mL) and stirred at room temperature for 48 hours. The organic phase was separated from the aqueous one. Volatiles were removed under vacuum. The solid residue was sublimed (65 °C, 3 x 10^{-2} mbar). In contrast to the reported procedure, no oil sublimed during the process despite a higher vacuum being used. ^{1}H and ^{11}B NMR analysis showed only unreacted $Mes^*B(OMe)_2$.

3.1.2 Attempts of hydrolysis of $Mes^*B(OMe)_2$

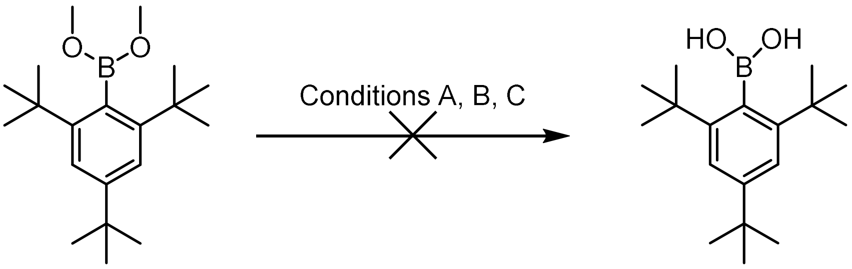

<u>Conditions A:</u>

This preparation was based on a modified literature procedure.[S7]

Aqueous HCl (1M, 1.3 mL) was added to a solution of $Mes^*B(OMe)_2$ (0.27 g, 0.85 mmol, 1 equiv.) in THF (5 mL) cooled at 0 °C. The solution was stirred for 3 hours. Water (5 mL) was added, the aqueous phase was extracted with hexane (3 x 5 mL). The combined organic phases were washed with a saturated solution of $NaHCO_3$ (5 mL) and brine (5 mL), dried with Na_2SO_4. Volatiles were removed *in vacuo*. The solid residue was extracted with hexane (20 mL). Removal of the hexane *in vacuo* afforded a white solid. ^{1}H and ^{11}B NMR analysis showed only unreacted $Mes^*B(OMe)_2$.

Conditions B:

This preparation was based on a modified literature procedure.[S8]

Aqueous HCl (1M, 5 mL) was added to a solution of Mes*B(OMe)$_2$ (0.27 g, 0.85 mmol, 1 equiv.) in THF (5 mL) at room temperature. The solution was stirred at 75 °C for 2 hours. The solution was allowed to cool down to room temperature. Water (5 mL) was added, the aqueous phase was extracted with hexane (3 x 5 mL). The combined organic phases were washed with saturated solution of $NaHCO_3$ (5 mL) and brine (5 mL), dried with Na_2SO_4. Volatiles were removed *in vacuo*. The solid residue was extracted with hexane (20 mL). Removal of the hexane *in vacuo* afforded a white solid. ^{1}H and ^{11}B NMR analysis showed only unreacted Mes*B(OMe)$_2$.

Conditions C:

Concentrated aqueous HCl (37% wt, 5 mL) was added to a solution of Mes*B(OMe)$_2$ (0.27 g, 0.85 mmol, 1 equiv.) in THF (5 mL) at room temperature. The solution was stirred at room temperature overnight. Water (5 mL) was added, the aqueous phase was extracted with hexane (3 x 5 mL). The combined organic phases were washed with saturated solutions of $NaHCO_3$ (5 mL) and brine (5 mL), dried with Na_2SO_4. Volatiles were removed *in vacuo*. The solid residue was extracted with hexane (20 mL). Removal of the hexane *in vacuo* afforded a white solid. ^{1}H and ^{11}B NMR analysis showed a mixture of unreacted Mes*B(OMe)$_2$:Mes* (1:0.5) alongside ring opened THF (See Figure S26 and S27).

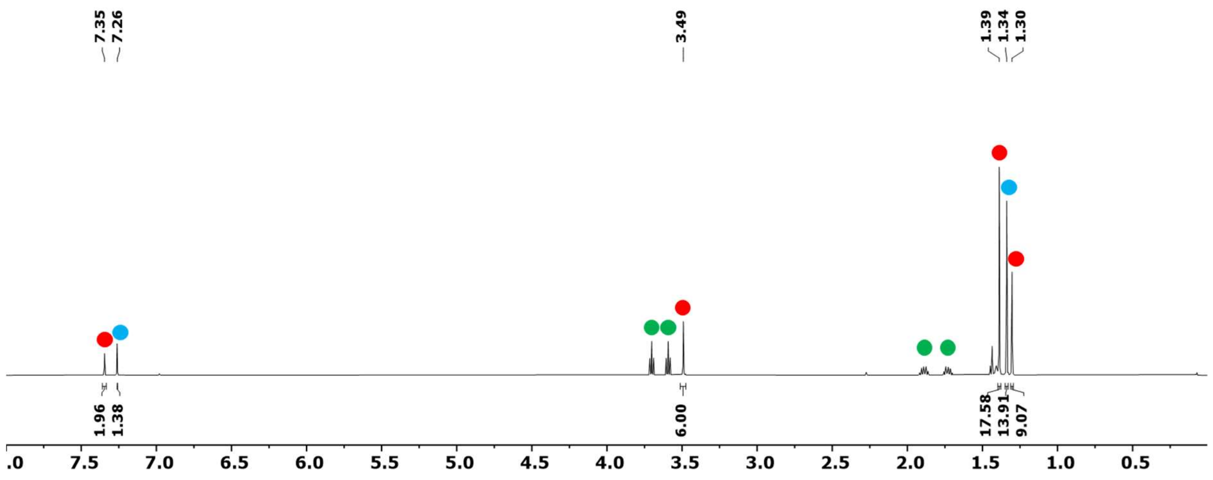

Figure S33: ^{1}H NMR spectrum (500 MHz, $CDCl_3$, 300 K) of the hydrolysis attempt of Mes*B(OMe)$_2$ using conditions C. The spectrum showed unreacted Mes*B(OMe)$_2$ (labelled in red), Mes* (labelled in blue, the aromatic C-H come very close to the solvent peak, partially overlapping with it) and ring opened THF (labelled in green). No evidence of hydrolysis product Mes*B(OH)$_2$ was observed. Mes*B(OMe)$_2$ proved to be very robust to protodeborylation even under very forcing conditions.

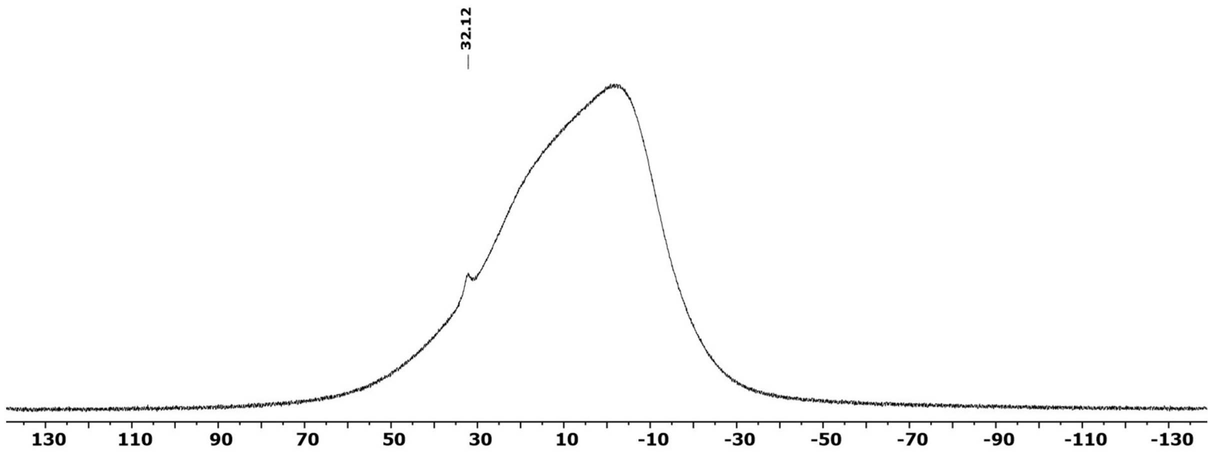

Figure S34: ^{11}B NMR spectrum (160 MHz, $CDCl_3$, 300 K) of the hydrolysis attempt of Mes*B(OMe)$_2$ using conditions C. The spectrum showed unreacted Mes*B(OMe)$_2$.

3.2 **Dehydration attempts of Mes*B(OH)$_2$**

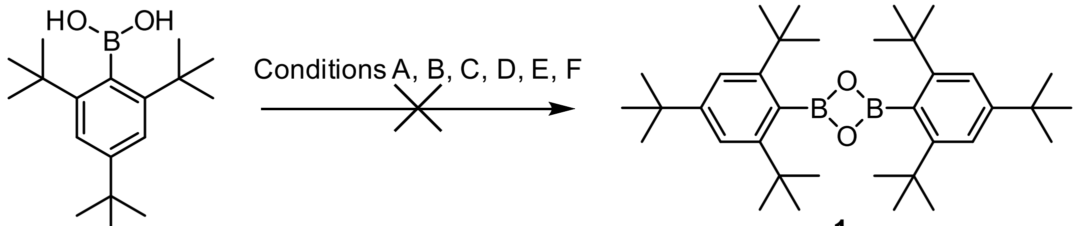

Conditions A:

Mes*B(OH)$_2$ (5 mg, 0.02 mmol) was loaded in a J. Youngs Ampoule and heated open to Argon atmosphere at various temperatures.

- 110 °C, 6h: No sublimation occurred, ^{1}H and ^{11}B NMR analysis only showed unreacted Mes*B(OH)$_2$.
- 180 °C, overnight: The compound decomposed. Only traces of Mes* were observed by ^{1}H NMR, ^{11}B NMR showed no signals.

Conditions B:

Mes*B(OH)$_2$ (0.1 g, 0.35 mmol or 5 mg, 0.02 mmol) was loaded in a J. Youngs Ampoule and heated under vacuum at various temperatures.

- 65 °C, 2 hours, 2-3 x 10^{-2} mbar: No sublimation occurred, ^{1}H and ^{11}B NMR analysis only showed unreacted Mes*B(OH)$_2$.
- 140 °C, overnight, 2-3 x 10^{-2} mbar: White solid sublimed, ^{1}H and ^{11}B NMR analysis only showed unreacted Mes*B(OH)$_2$.
- 180 °C, 20 minutes, 2-3 x 10^{-2} mbar: White solid sublimed quickly, ^{1}H and ^{11}B NMR analysis only showed unreacted Mes*B(OH)$_2$.

Conditions C:

Mes*B(OH)$_2$ (0.10 g, 0.35 mmol, 1 equiv.) and Al_2O_3 (0.01 g, 10% by weight) were loaded in a J. Youngs Ampoule and heated under vacuum (2-3 x 10^{-2} mbar) at 120 °C. A white solid sublimed, ^{1}H and ^{11}B NMR analysis only showed unreacted Mes*B(OH)$_2$.

Conditions D:

This procedure was based on a modified literature procedure.[S9]

Mes*B(OH)$_2$ (0.10 g, 0.35 mmol) was dissolved in toluene (10 mL) and refluxed overnight using a dean stark apparatus, opened to Argon atmosphere. The solution was left to cool down to room temperature and volatiles were removed *in vacuo*. ^{1}H and ^{11}B NMR NMR analysis showed unreacted Mes*B(OH)$_2$.

Conditions E:

Mes*B(OH)$_2$ (0.10 g, 0.35 mmol, 1 equiv.) and Al_2O_3 (0.01 g, 10% by weight) were dissolved in toluene (10 mL) and refluxed overnight using a dean stark apparatus, opened to Argon atmosphere. The solution was left to cool down to room temperature and volatiles were removed *in vacuo*. ^{1}H and ^{11}B NMR NMR analysis showed unreacted Mes*B(OH)$_2$.

Conditions F:

Mes*B(OH)$_2$ (0.10 g, 0.35 mmol, 1 equiv.) and P_2O_5 (0.05 g, 0.38 mmol, 1.1 equiv.) were dissolved in 1,2-dichlorobenzene (10 mL) and refluxed overnight using a dean stark apparatus, opened to Argon atmosphere. The solution was left to cool down to room temperature and volatiles were removed *in vacuo*. ^{1}H and ^{11}B NMR analysis showed decomposition to an intractable mixture of products.

3.3 Attempt of thermal elimination of TMSCl from 6, Mes*BCl(OTMS)

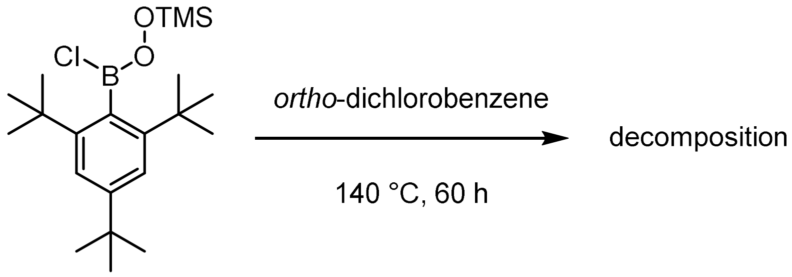

Compound **6**, Mes*BCl(OTMS) (10 mg, 0.03 mmol, 1 equiv.) was dissolved in *ortho*-dichlorobenzene (0.5 mL) in a J. Youngs NMR tube and heated at 140 °C for 60 hours. ^{11}B and ^{29}Si NMR spectroscopy showed decomposition to an intractable mixture of products. No evidence of $\mathbf{1}_2$ or **7** were observed.

4 Complementary studies

4.1 DOSY experiment of Mes*BO-$AlCl_3$, 7

The normalization of the diffusion coefficients was carried out following the method published by Stalke *et al.*[S10]

Adamantane was used as internal standard.

Mes*BO-$AlCl_3$ **7** was considered to be diffuse like dissipated spheres and ellipsoids (DSE).

Mes*BCl(OTMS), **6** (23 mg, 0.06 mmol, 1.00 equiv.) and $AlCl_3$ (10 mg, 0.07 mmol, 1.20 mmol) were combined in C_6D_6 (1 mL) in a J. Young's NMR tube, generating Mes*BO-$AlCl_3$, **7** (maximum theoretical yield: 0.06 mmol, 60 mM). Adamantane (10 mg, 0.07 mmol, 70 mM) was added after 1 hour. The chosen concentrations were within the range of the valid concentrations (< 120 mM).[S10] The normalization of the diffusion coefficient was calculated using the following equation.

$$log\, D_{\text{substrate,norm}} = log\, D_{\text{ref,fix}} - log\, D_{\text{ref}} + log\, D_{\text{substrate}}$$

The value $log\, D_{\text{ref,fix}}$ = –8.8803 was found in the literature from Stalke *et al.*[S11]

The values of $log\, D_{\text{ref}}$ = –8.8818 and $log\, D_{\text{substrate}}$ = –9.1341 were measured experimentally by DOSY NMR experiment.

$$log\, D_{\text{substrate,norm}} = -8.8803 + 8.8819 - 9.1339$$

$$log\, D_{\text{substrate,norm}} = -9.1323$$

$$D_{\text{substrate,norm}} = 7.3740 \times 10^{-10}$$

The molecular weight was calculated using the power law developed by Williard *et al.*[S12]

$$D = K \times MW^{\alpha}$$

As considered diffusing like DSE, the value of $log\, K$ = –7.47 and α = –0.622 were found from the literature.[S11]

$$D_{\text{substrate,norm}} = 7.3740 \times 10^{-10} = 3.3884 \times 10^{-8} \times MW^{-0.622}$$

$$MW = 470.4441$$

The experimental molecular weight determination of Mes*BO-$AlCl_3$ **7**, MW = 470.44 g.mol^{-1} was found within the range of the theoretical value (405.59 g.mol^{-1}) with 16% error.

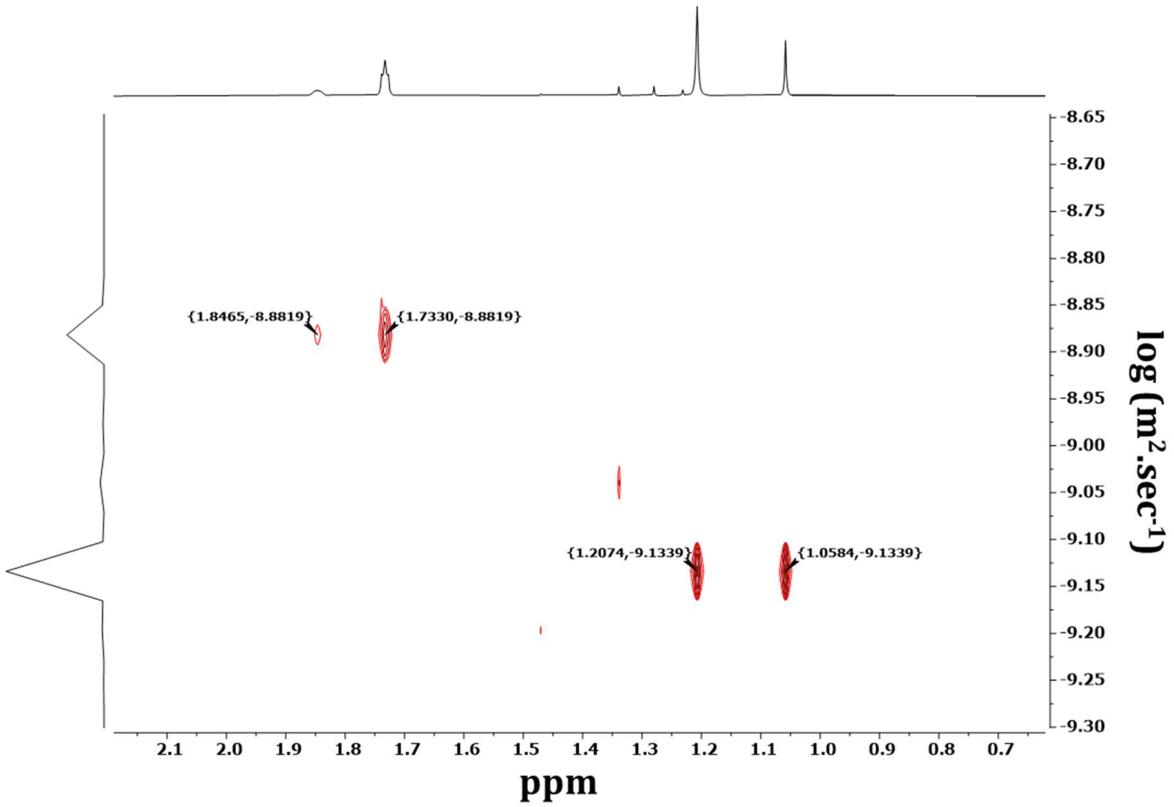

Figure S35: DOSY spectrum of the Mes*BO-$AlCl_3$ **7** with internal standard adamantane.

4.2 *In situ* NMR of generation of 9, observation of isobutylene

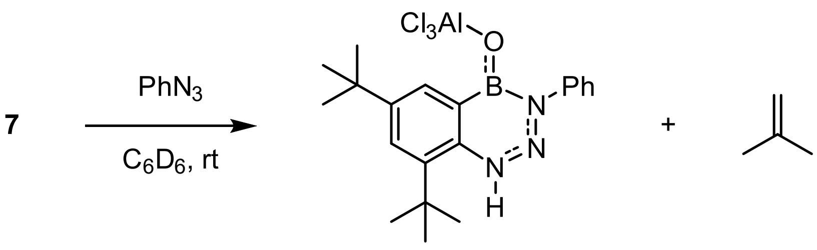

9

PhN_3 (3 µL, 0.03 mmol, 1.00 equiv.) was added to a solution of Mes*BO-$AlCl_3$ **7** (10 mg, 0.03 mmol, 1.00 equiv.) in C_6D_6 (0.5 mL) in a J. Young's NMR tube at room temperature. The reaction was monitored by NMR spectroscopy (Figure S36).

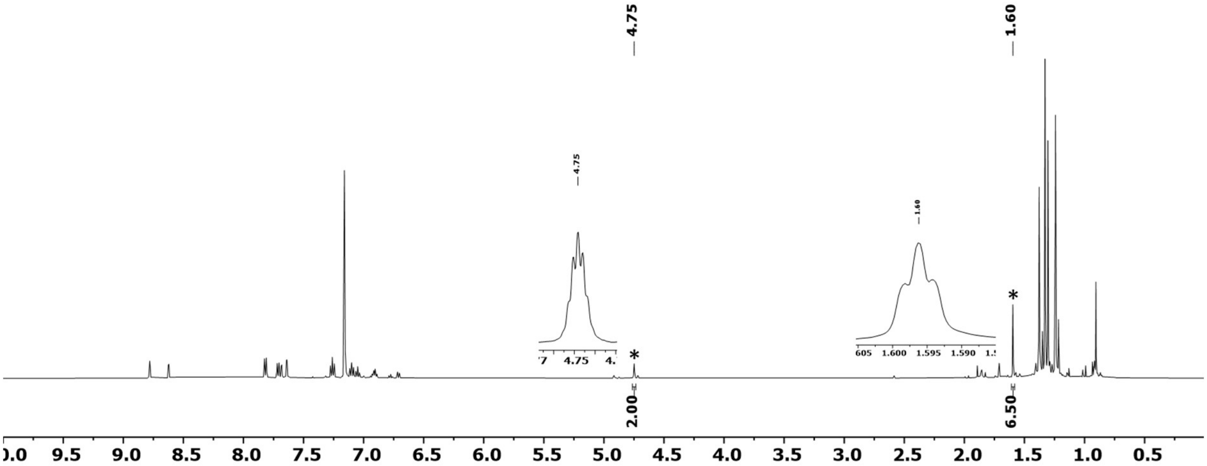

Figure S36: ^{1}H NMR spectrum (500 MHz, C_6D_6, 300 K) of the reaction between **7** and PhN_3. Alongside the formation of product **9**, generation of isobutylene is observed by ^{1}H NMR spectroscopy (labelled *) with the signals at δ 4.75 (sept, $^4J_{HH}$ = 1.2 Hz, $\underline{H}_2C=C(CH_3)_2$, 2H) and at δ 6.50 (t, $^4J_{HH}$ = 1.1 Hz, $H_2C=C(C\underline{H}_3)_2$, 6H). Signals were in accordance with the literature data.[S13]

4.3 **Equilibrium between 9 and an isomer, termed 9B**

NMR analysis of compound **9** also revealed the presence of a minor unknown species, **9B**. **9B** featured a similar pattern in the ^{1}H NMR spectrum suggesting a possible equilibrium between **9** and an isomer. To assess this the equilibrium was studied through temperature and solvent variation.

4.3.1 VT experiments

Compound **9** (10 mg, 0.02 mmol) was dissolved in CD_2Cl_2 (0.4 mL) in a J. Young's NMR tube. VT NMR experiments were run at 5 and 30 °C. No change in ratio was observed between **9** and the unknown species, remaining at 1:0.1, **9**:**9B** (Figure S37).

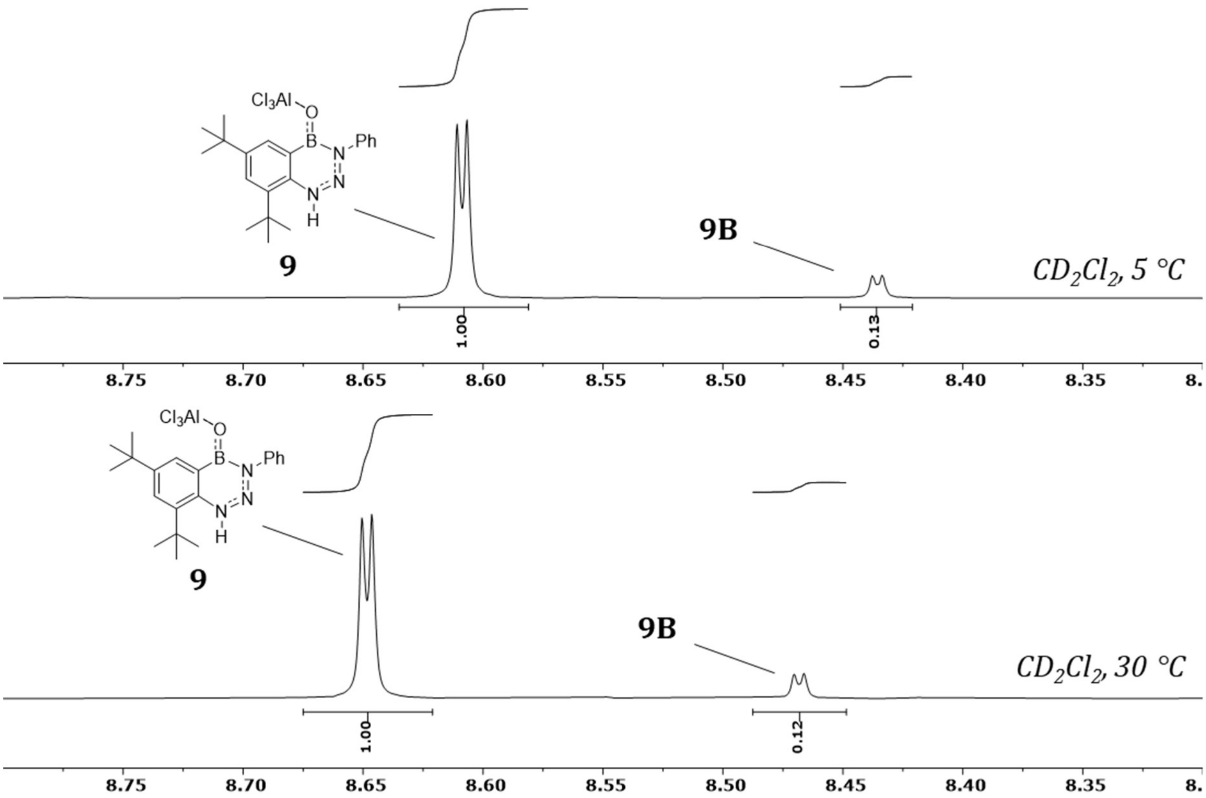

Figure S37: Top, ^{1}H NMR spectrum (500 MHz, CD_2Cl_2, 278 K) of **9** at 5 °C. Bottom, ^{1}H NMR spectrum (500 MHz, CD_2Cl_2, 303 K) of **9** at 30 °C. The spectra are focused on the aromatic region, where distinguishable peaks of **9** and **9B** compound could easily be integrated for comparison. Note that upon heating, a slight shift is observed, but no significant ratio change is seen by ^{1}H NMR.

4.3.2 Equilibration in different solvents

Compound **9** (10 mg, 0.02 mmol) was dissolved in CD_2Cl_2 (0.4 mL). After NMR analysis, the solvent was removed *in vacuo* and the residue was redissolved in C_6D_6 (2 mL, note that more solvent had to be used to obtain a homogenous sample due to the lower solubility of **9** in C_6D_6). After NMR analysis, the solvent was removed *in vacuo* and the residue was redissolved in CD_2Cl_2.

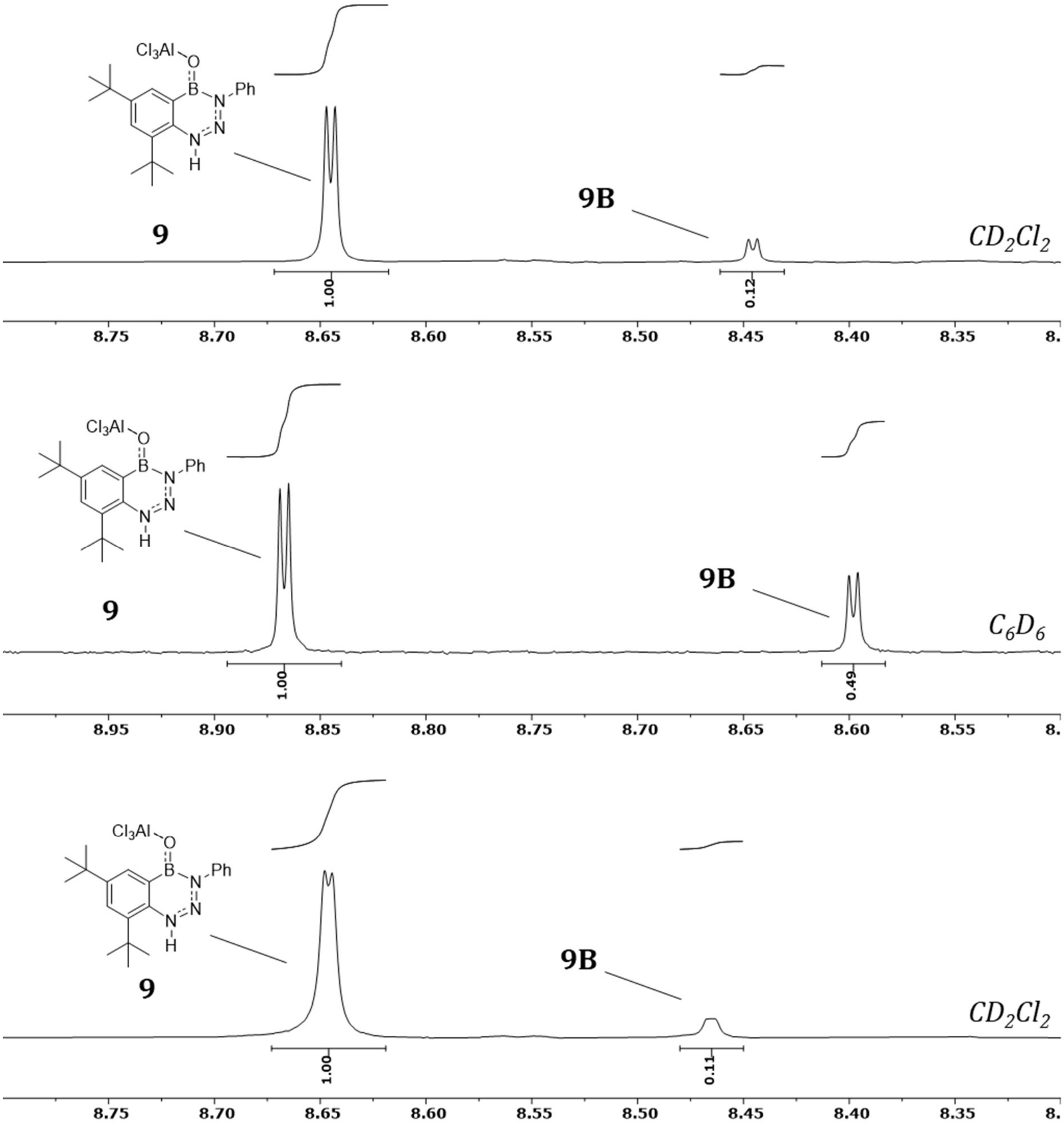

Figure S38: Top, ^{1}H NMR spectrum (500 MHz, CD_2Cl_2, 300 K) of **9** in CD_2Cl_2. Middle, ^{1}H NMR spectrum (500 MHz, C_6D_6, 300 K) of **9** in C_6D_6, after CD_2Cl_2 removal. Bottom, ^{1}H NMR spectrum (500 MHz, CD_2Cl_2, 300 K) of **9** in CD_2Cl_2, after C_6D_6 removal.

In the ¹H NMR, the ratio **9**:**9B** changed when the solvent was switched from CD_2Cl_2 to C_6D_6, going from 1:0.1 to 1:0.5 (Figure S38, top and middle). Going from C_6D_6 back to CD_2Cl_2 resulted in the system reaching the 1:0.1 ratio observed originally (Figure S38, middle and bottom), confirming the dynamic equilibrium between compound **9** and **9B**.

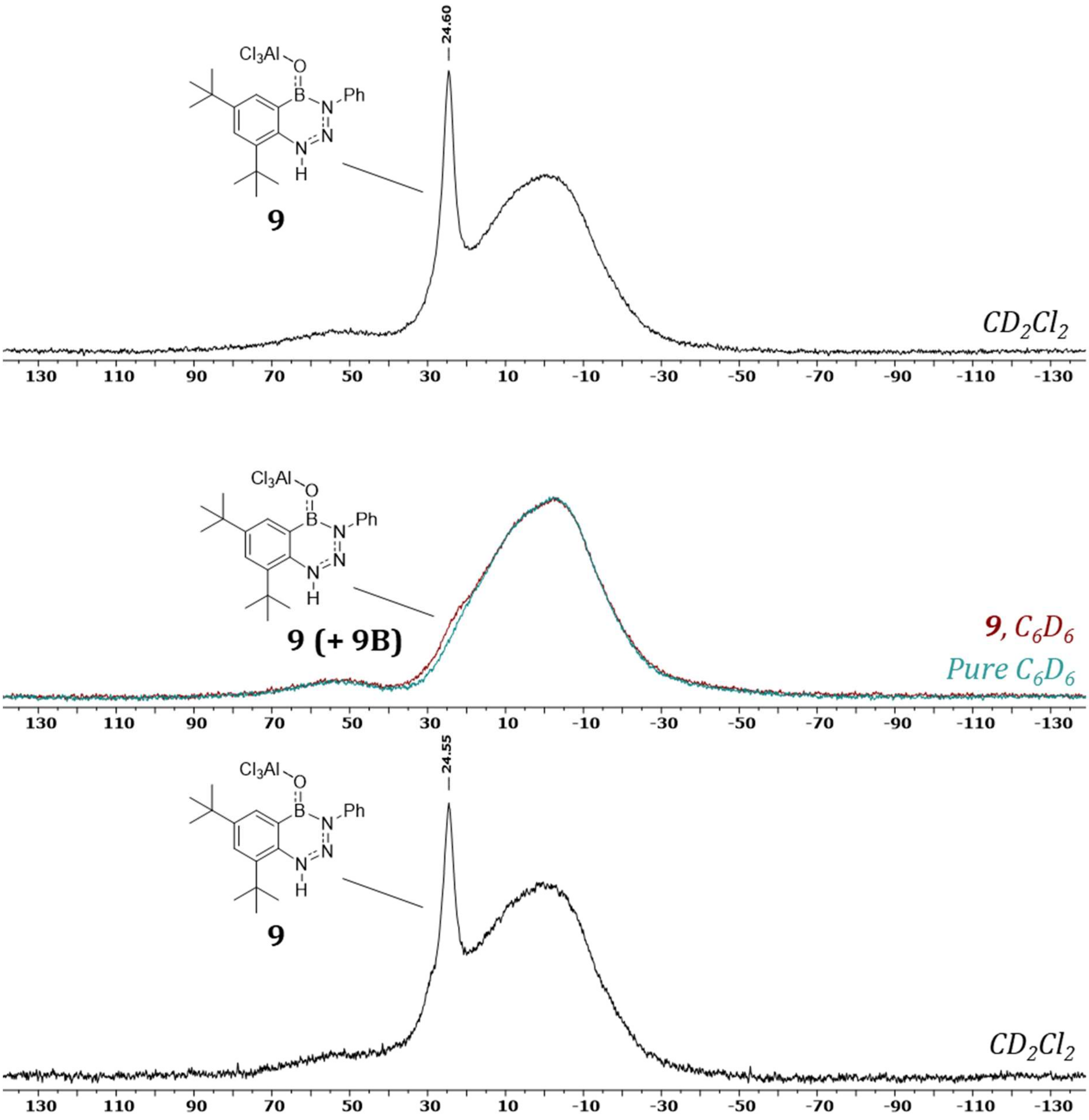

Figure S39: Top, ^{11}B NMR spectrum (160 MHz, CD_2Cl_2, 300 K) of **9** in CD_2Cl_2. Middle, ^{11}B NMR spectrum (160 MHz, C_6D_6, 300 K) of **9** in C_6D_6 after CD_2Cl_2 removal (red). Due to the very broad and hardly visible signal of **9** in C_6D_6, a ^{11}B NMR spectrum of pure C_6D_6 was recorded (teal) and overlapped for clarity. Bottom, ^{11}B NMR spectrum (160 MHz, CD_2Cl_2, 300 K) of **9** in CD_2Cl_2 after C_6D_6 removal.

In the ^{11}B NMR, the signal goes from a very distinct singlet at δ 24.6 to a very broad signal between δ 30 and 15 when the solvent was changed from CD_2Cl_2 to C_6D_6 (Figure S39, top and middle).

Going from C_6D_6 back to CD_2Cl_2 allowed to observed the signal at δ 24.6 from **9** again (Figure S39, middle and bottom).

The equilibrium between **9** and **9B** changing drastically when solvent was changed from CD_2Cl_2 to C_6D_6 consistent with an isomerism occurring in solution. The compound **9** was effectively pure and the minor species **9B** is an isomer of **9**.

4.3.3 Proposed structure and NMR data of 9B

As the equilibration in solvent switching studies clearly evidenced the presence of a minor isomer **9B** in equilibrium with **9** in solution, we tried to identify this species computationally.

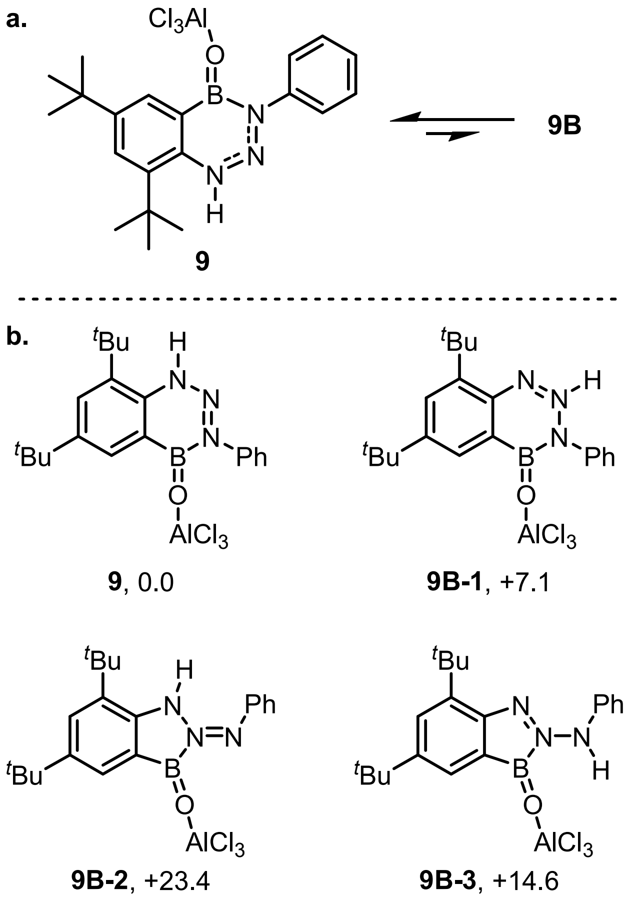

Figure S40: a. Schematic representation of the equilibrium between **9** and **9B** in solution. **b.** Calculated structures that are isomers of **9** and free energy (kcal.mol^{-1}) relative to the observed compound **9**.

The calculated energies of the postulated isomers **9B-1**, **9B-2** and **9B-3** energies were found to be too high compared with **9** to fit with the observation of our equilibrium in solution (Figure S40-b). Therefore, the identity of the minor isomer is currently not known.

We tentatively did the NMR assignment **9B** in C_6D_6. However, the presence of isomer **9B** in significant amount required the use of apolar solvents (e.g. C_6D_6), which also involved a decrease in the solubility of **9** and **9B**. Therefore, only partial NMR data could be collected for **9B**.

NMR Data of **9B**:

^{1}H NMR (500 MHz, C_6D_6, 300 K) δ 8.60 (d, $^4J_{HH}$ = 2.1 Hz, 1H), 7.73-7.70 (m, 2H), 7.65 (d, $^4J_{HH}$ = 2.1 Hz, 1H), 7.10-7.05 (m, 2H), 6.85-6.80 (m, 1H), 1.29 (s, 9H), 1.29 (s, 9H).

^{11}B NMR (160 MHz, C_6D_6, 300 K) δ 24.0 (v. br s).

The low solubility of **9B** in C_6D_6 resulted in no signal in the ^{27}Al NMR.

Assignment of the ^{13}C NMR was not possible even using 2D-NMR experiments (^{1}H-^{13}C HSQC/HMBC), due to the low intensity of the carbon signals (caused by the low solubility of **9B** in C_6D_6), coupled to the overlap with the signals of the major compound **9**.

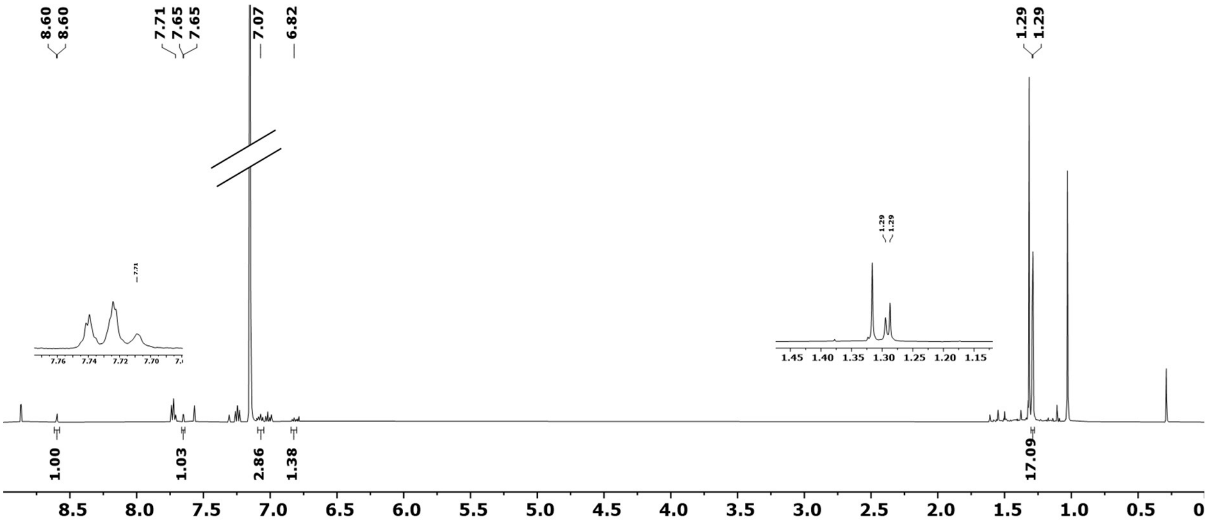

Figure S41: ^{1}H NMR spectrum (500 MHz, C_6D_6, 300 K) of **9B**. *Only the signal from **9B** are labelled on the spectrum. The multiplet at 7.73-7.70 is overlapped with signal from **9**. The multiplet at 7.10-7.05 is overlapped with residual signal from PhCl.

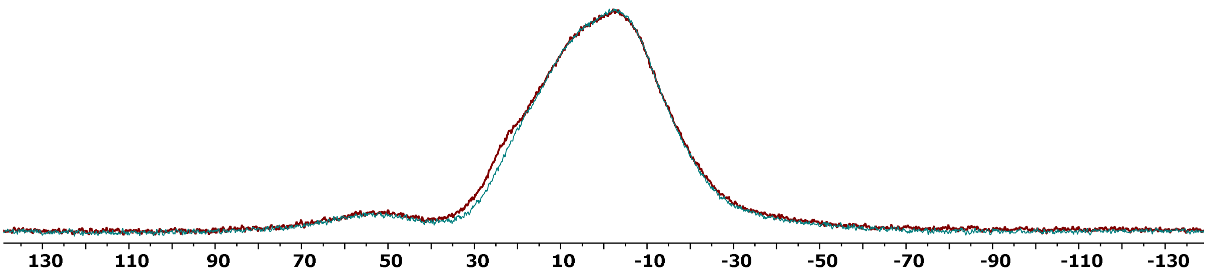

Figure S42: ^{11}B NMR spectrum (160 MHz, C_6D_6, 300 K) of **9B**. (red). Due to the very broad and hardly visible signal of **9** in C_6D_6, a ^{11}B NMR spectrum of pure C_6D_6 was recorded (teal) and overlapped for clarity.

5 Crystallographic data

5.1 Crystal structure of Mes*BCl(OTMS) 6

CCDC Deposition Number: 2383097

Experimental: Single clear colourless prism-shaped crystals of **7** recrystallised spontaneously from oil. A suitable crystal with dimensions 0.23 × 0.15 × 0.07 mm³ was selected and mounted on a MITIGEN holder NVH oil on a SuperNova, Dual, Cu at home/near, Atlas diffractometer. The crystal was kept at a steady T = 120.00(10) K during data collection. The structure was solved with the ShelXS (Sheldrick, 2008)[S16] solution program using direct methods and by using Olex2 1.5-beta (Dolomanov et al., 2009)[S14] as the graphical interface. The model was refined with ShelXL 2018/3 (Sheldrick, 2015)[S15] using full matrix least squares minimisation on F^2.

**Compound**	**6**
Formula	$C_{21}H_{38}BClOSi$
$D_{calc.}$/ g cm^{-3}	1.093
μ/mm^{-1}	1.982
Formula Weight	380.86
Colour	clear colourless
Shape	prism-shaped
Size/mm³	0.23×0.15×0.07
T/K	120.00(10)
Crystal System	triclinic
Space Group	P-1
a/Å	6.19982(14)
b/Å	12.8169(2)
c/Å	29.2199(6)
α/°	91.3018(14)
β/°	90.7335(17)
γ/°	94.3215(16)
V/Å³	2314.44(8)
Z	4
Z'	2
Wavelength/Å	1.54184
Radiation type	Cu K_α
Θ_{min}/°	3.459
Θ_{max}/°	76.559
Measured Refl's.	11336
Indep't Refl's	11336
Refl's I≥2 σ(I)	10942
R_{int}	0.1142
Parameters	476
Restraints	0
Largest Peak	0.461
Deepest Hole	-0.499
GooF	1.247
wR_2 (all data)	0.1574
wR_2	0.1568
R_1 (all data)	0.0638
R_1	0.0620

5.2 Crystal structure of Mes*BO-$AlCl_3$ 7

CCDC Deposition Number: 2383099

Experimental: Single colourless plate-shaped crystals of **7** recrystallised from toluene-d8 by slow evaporation. A suitable crystal with dimensions 0.29 × 0.08 × 0.02 mm^3 was selected and mounted on a mitegen tip in paratone oil on a Bruker D8 VENTURE diffractometer. The crystal was kept at a steady T = 100.00 K during data collection. The structure was solved with the ShelXS (Sheldrick, 2008)[S16] solution program using direct methods and by using Olex2 1.5-beta (Dolomanov et al., 2009)[S14] as the graphical interface. The model was refined with ShelXL 2018/3 (Sheldrick, 2015)[S15] using full matrix least squares minimisation on F^2.

**Compound**	**7**
Formula	$C_{18}H_{29}AlBCl_3O$
$D_{calc.}$/ g cm^{-3}	1.227
μ/mm^{-1}	0.461
Formula Weight	405.55
Colour	colourless
Shape	plate-shaped
Size/mm^3	0.29×0.08×0.02
T/K	100.00
Crystal System	monoclinic
Space Group	$P2_1/m$
a/Å	9.7361(4)
b/Å	9.8431(3)
c/Å	11.6371(4)
α/°	90
β/°	100.2024(19)
γ/°	90
V/$Å^3$	1097.59(7)
Z	2
Z'	0.5
Wavelength/Å	0.71073
Radiation type	MoK_α
Θ_{min}/°	2.125
Θ_{max}/°	25.365
Measured Refl's.	38913
Indep't Refl's	2139
Refl's I≥2 σ(I)	1800
R_{int}	0.0642
Parameters	196
Restraints	0
Largest Peak	0.298
Deepest Hole	-0.374
GooF	1.081
wR_2 (all data)	0.1139
wR_2	0.1073
R_1 (all data)	0.0526
R_1	0.0437

5.3 Crystal structure of Mes*(DMAP)BO-AlCl$_3$ 8

CCDC Deposition Number: 2383098

Experimental: Single colourless block-shaped crystals of **8** recrystallised from a mixture of C_6D_6 and pentane by solvent layering. A suitable crystal with dimensions 0.47 × 0.15 × 0.09 mm^3 was selected and mounted on a MITIGEN holder in paratone oil on a Rigaku Oxford Diffraction SuperNova diffractometer. The crystal was kept at a steady T = 120.00(10) K during data collection. The structure was solved with the ShelXT 2018/2 (Sheldrick, 2018)[S17] solution program using dual methods and by using Olex2 1.5-beta (Dolomanov et al., 2009)[S14] as the graphical interface. The model was refined with ShelXL 2018/3 (Sheldrick, 2015)[S15] using full matrix least squares minimisation on F^2.

**Compound**	**8**
Formula	$C_{56}H_{78}Al_2B_2Cl_6D_6N_4O_2$
$D_{calc.}$/ g cm^{-3}	1.222
μ/mm^{-1}	3.121
Formula Weight	1139.58
Colour	colourless
Shape	block-shaped
Size/mm^3	0.47×0.15×0.09
T/K	120.00(10)
Crystal System	orthorhombic
Space Group	*Pbca*
a/Å	17.83430(10)
b/Å	18.2314(2)
c/Å	19.0578(2)
α/°	90
β/°	90
γ/°	90
V/Å^3	6196.53(10)
Z	4
Z'	0.5
Wavelength/Å	1.54184
Radiation type	Cu K_α
Θ_{min}/°	4.172
Θ_{max}/°	75.937
Measured Refl's.	65364
Indep't Refl's	6431
Refl's I≥2 σ(I)	5933
R_{int}	0.0355
Parameters	348
Restraints	0
Largest Peak	0.502
Deepest Hole	-0.399
GooF	1.044
wR_2 (all data)	0.0927
wR_2	0.0900
R_1 (all data)	0.0373
R_1	0.0344

5.4 **Crystal structure of 9**

CCDC Deposition Number: 2386780

Fomblin oil was used to coat a selection of crystals of **9** which were then mounted on MiTeGen kapton loops and frozen in liquid nitrogen. The loops were stored in a MiTeGen Unipuck and transported to Diamond Light Source. Data were collected remotely at beam line I-19 of Diamond Light Source (award CY35994), yielding structure of **9**.[S18]

**Compound**	**9**
Formula	$C_{27}H_{34}AlBCl_3N_3O$
$D_{calc.}$/ g cm^{-3}	1.283
μ/mm^{-1}	0.343
Formula Weight	560.71
Colour	colourless
Shape	block-shaped
Size/mm^3	0.05×0.05×0.05
T/K	100.00
Crystal System	monoclinic
Space Group	$P2_1/n$
a/Å	9.80810(15)
b/Å	23.4007(6)
c/Å	13.2628(3)
α/°	90
β/°	107.4970(15)
γ/°	90
V/Å^3	2903.19(10)
Z	4
Z'	1
Wavelength/Å	0.6889
Radiation type	synchrotron
Θ_{min}/°	1.687
Θ_{max}/°	20.151
Measured Refl's.	25707
Indep't Refl's	3041
Refl's I≥2 σ(I)	2172
R_{int}	0.1025
Parameters	335
Restraints	0
Largest Peak	0.701
Deepest Hole	-0.345
GooF	0.986
wR_2 (all data)	0.1473
wR_2	0.1397
R_1 (all data)	0.0665
R_1	0.0504

6 Computational details

General

All calculations were performed using Gaussian 16.[S19] Conformers for each compound were generated and ranked using CREST v2.12[S20] at the GFN2-xTB(ALPB: benzene) level of theory,[S21] and the lowest energy conformer taken forward for further calculations. Geometry optimisation was carried out at the PBE0-D3(BJ)/Def2-SVP(SMD: benzene) level of theory[S22-S25] and frequency calculations carried out at the same level to confirm a minima (no imaginary frequencies) or saddle-point (a single imaginary frequency) and obtain thermochemical corrections. A single-point correction was calculated at the MN15/Def2-TZVPP(SMD: benzene) level of theory[S22,S25,S26] and combined with the previous thermochemical corrections. ^{11}B NMR shielding tensors were calculated at the MN15/Def2-TZVPP(SMD: benzene) level of theory using the Gauge-Independent Atomic Orbital (GIAO) method[S27] and ppm values calculated using Multiwfn v3.8[S28] with $Et_2O{\cdot}BF_3$ as the reference. 3D images were generated using ChemCraft[S29] and orbitals visualised using Avogadro.[S30] The default scaling factor provided in Gaussian16 (1.0) was used when calculating stretching frequencies.

Hydride Ion Affinities

According to our previous method:[S31] the lowest energy conformer was optimised at the M06-2X/6-311G(d,p)(IEFPCM: dichloromethane) level of theory,[S32–S35] followed by a frequency calculation at the same level of theory to confirm a minima (no imaginary frequencies) and obtain thermochemical corrections.

6.1 Formation of 9 Free Energy Profile

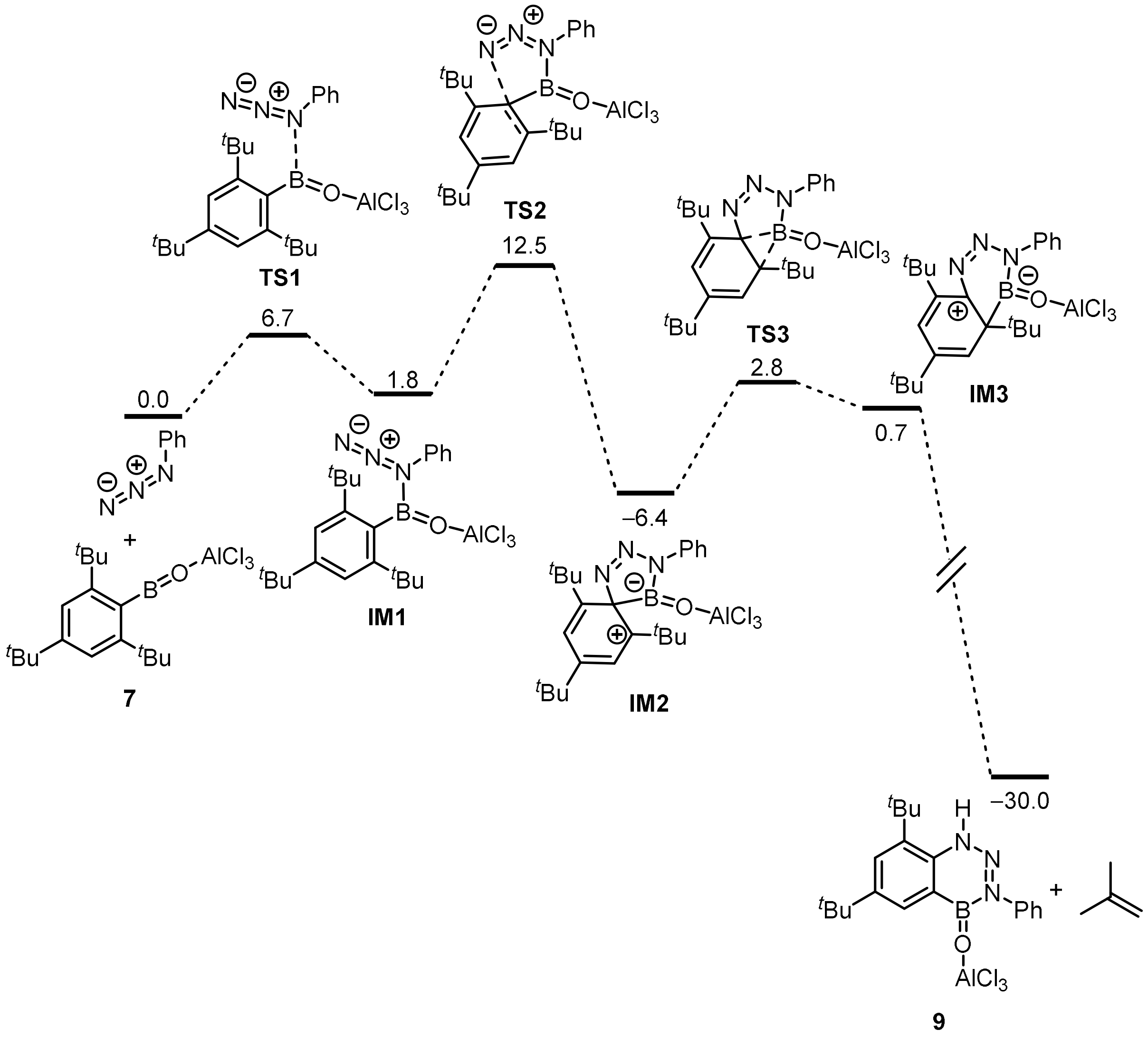

Figure S43: Free energy profile (kcal mol[-1]) for the formation of compound **9** from compound **7** and PhN_3.

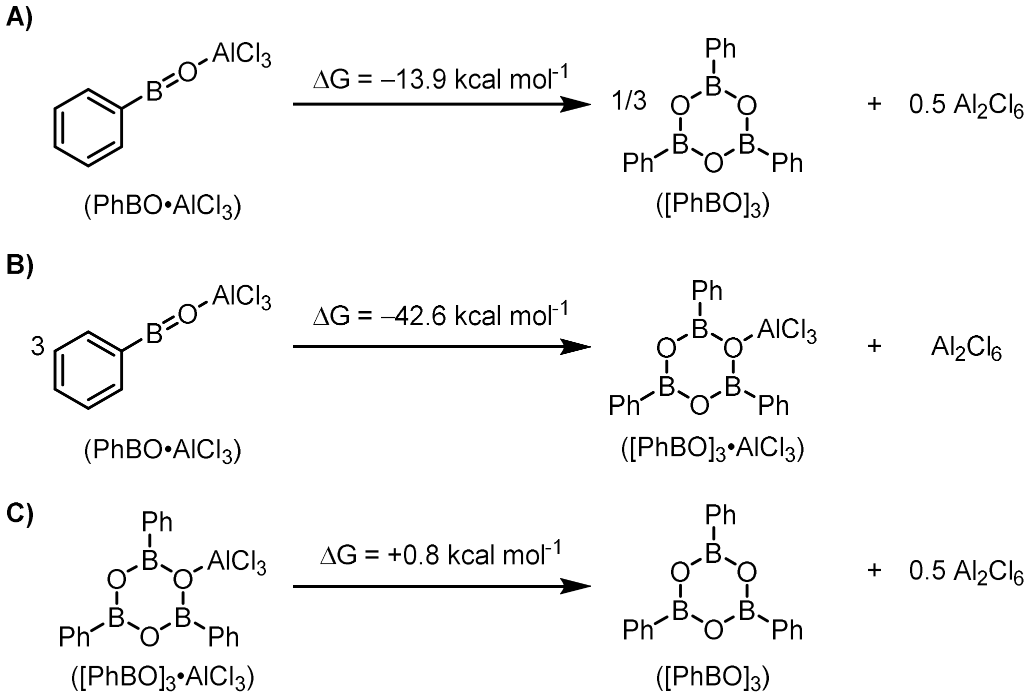

Figure S44. A) Free energy change of triphenylboroxine formation from $PhBO{\cdot}AlCl_3$. B) Free energy change of triphenylboroxine$\cdot AlCl_3$ formation from $3PhBO{\cdot}AlCl_3$. C) Free energy change of loss of $AlCl_3$ from triphenylboroxine$\cdot AlCl_3$.

6.2 Cartesian Coordinates

6.2.1 Compound 1 at PBE0-D3(BJ)/Def2-SVP(SMD: benzene)

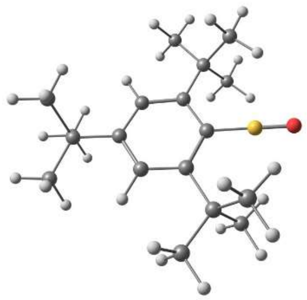

Compound 1 at PBE0-D3(BJ)/Def2-SVP(SMD: benzene)

B	2.638930000	0.100878000	-0.000106000
O	3.850952000	0.154225000	-0.000090000
C	1.116604000	0.034373000	0.000001000
C	0.469669000	-1.225733000	-0.000213000
C	0.359630000	1.236385000	0.000246000
C	-0.927618000	-1.253728000	-0.000236000
C	-1.029519000	1.138104000	0.000209000
C	-1.697270000	-0.091072000	-0.000057000
H	-1.431993000	-2.215667000	-0.000413000
H	-1.623346000	2.050375000	0.000404000
C	1.267170000	-2.540461000	-0.000494000
C	2.141544000	-2.616099000	1.260610000
H	2.892105000	-1.813341000	1.308321000
H	1.523563000	-2.557536000	2.170012000
H	2.692429000	-3.569616000	1.282865000
C	0.354030000	-3.769173000	-0.000123000
H	-0.288868000	-3.810311000	-0.892611000
H	0.972500000	-4.679436000	0.000051000
H	-0.288748000	-3.809827000	0.892478000
C	1.904081000	2.765319000	1.262036000
H	1.293158000	2.652086000	2.171036000
H	2.721990000	2.031295000	1.309540000
C	0.024005000	3.759465000	0.000788000
H	-0.619699000	3.744493000	0.893572000
H	0.561209000	4.719952000	0.000931000
H	-0.619819000	3.744811000	-0.891914000
C	1.040178000	2.614768000	0.000504000
C	1.903985000	2.765891000	-1.261021000
H	1.292985000	2.653125000	-2.170027000
H	2.369498000	3.763842000	-1.283952000
H	2.721886000	2.031889000	-1.308963000
H	2.369572000	3.763269000	1.285422000
C	2.140526000	-2.616059000	-1.262312000
H	2.691488000	-3.569522000	-1.285003000
H	1.521782000	-2.557582000	-2.171202000

H	2.890908000	-1.813177000	-1.310624000
C	-3.227014000	-0.117274000	0.000000000
C	-3.741278000	0.604612000	-1.253876000
C	-3.740979000	0.602846000	1.255021000
H	-3.380989000	0.107916000	2.170516000
H	-3.413853000	1.652768000	1.290834000
H	-4.842599000	0.596624000	1.275429000
H	-3.414306000	1.654624000	-1.288282000
H	-4.842900000	0.598311000	-1.274053000
H	-3.381449000	0.111025000	-2.170160000
C	-3.782277000	-1.541496000	-0.000967000
H	-3.469567000	-2.106736000	0.890540000
H	-4.882629000	-1.510293000	-0.001032000
H	-3.469389000	-2.105569000	-0.893153000

6.2.2 Compound 1_2

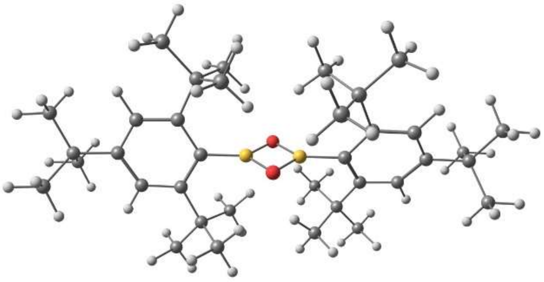

Compound 1_2 at PBE0-D3(BJ)/Def2-SVP(SMD: benzene)

B	0.935247000	0.014909000	0.001182000
B	-0.935226000	0.014958000	-0.001146000
O	-0.001268000	0.016656000	1.060085000
O	0.001210000	0.012907000	-1.060044000
C	-2.509819000	0.014787000	-0.003333000
C	-3.242269000	-1.183727000	0.255869000
C	-3.240312000	1.209031000	-0.263868000
C	-4.636536000	-1.144540000	0.247976000
C	-4.639696000	1.172801000	-0.261152000
C	-5.365339000	0.015441000	-0.007835000
H	-5.186867000	-2.061302000	0.446827000
H	-5.182641000	2.090744000	-0.462405000
C	2.509809000	0.014776000	0.003316000
C	3.242273000	-1.183718000	-0.255939000
C	3.240319000	1.209004000	0.263897000
C	4.636536000	-1.144523000	-0.248132000
C	4.639696000	1.172744000	0.261357000
C	5.365341000	0.015412000	0.007904000
H	5.186864000	-2.061252000	-0.447154000
H	5.182636000	2.090652000	0.462803000
C	-2.565189000	2.566767000	-0.540879000
C	-2.566085000	-2.540071000	0.534537000
C	2.565265000	2.566797000	0.540698000
C	2.566099000	-2.540069000	-0.534510000
C	-1.630426000	2.917108000	0.621570000
H	-1.148887000	3.891727000	0.451299000
H	-2.196162000	2.974607000	1.564421000
H	-0.829559000	2.186394000	0.779807000
C	-3.570401000	3.720792000	-0.640004000
H	-3.018455000	4.659494000	-0.801336000
H	-4.263803000	3.605483000	-1.486470000
H	-4.161712000	3.841146000	0.280558000
C	-1.822417000	2.517372000	-1.884845000
H	-2.537899000	2.340274000	-2.703092000
H	-1.318145000	3.477214000	-2.080474000
H	-1.067829000	1.723692000	-1.935862000
C	-1.825564000	-2.489150000	1.879635000
H	-1.072261000	-1.694314000	1.931794000

H	-2.542703000	-2.312932000	2.696608000
H	-1.319995000	-3.448087000	2.076272000
C	-1.629206000	-2.889595000	-0.626420000
H	-0.829021000	-2.157856000	-0.783700000
H	-1.146738000	-3.863548000	-0.455085000
H	-2.193558000	-2.948086000	-1.570041000
C	-3.570841000	-3.694309000	0.632522000
H	-4.160097000	-3.815269000	-0.289324000
H	-3.019280000	-4.632902000	0.795821000
H	-4.265872000	-3.577988000	1.477560000
C	1.824192000	2.518384000	1.885654000
H	1.320326000	3.478458000	2.081240000
H	1.069529000	1.724864000	1.937923000
H	2.540678000	2.341770000	2.703125000
C	3.570404000	3.721092000	0.637376000
H	3.018485000	4.659945000	0.797913000
H	4.264544000	3.607077000	1.483413000
H	4.160916000	3.840204000	-0.283862000
C	1.628867000	2.915957000	-0.620717000
H	2.193121000	2.972677000	-1.564498000
H	0.827830000	2.185037000	-0.777121000
H	1.147270000	3.890562000	-0.450605000
C	3.570677000	-3.694664000	-0.630120000
H	4.266451000	-3.579656000	-1.474725000
H	4.159119000	-3.814489000	0.292396000
H	3.019053000	-4.633348000	-0.792680000
C	1.627527000	-2.888415000	0.625351000
H	0.827262000	-2.156388000	0.780847000
H	1.144871000	-3.862269000	0.454056000
H	2.190353000	-2.946296000	1.569916000
C	1.827281000	-2.489941000	-1.880586000
H	1.322050000	-3.449055000	-2.077287000
H	1.073954000	-1.695208000	-1.933881000
H	2.545421000	-2.314159000	-2.696771000
C	6.894487000	-0.024894000	-0.001233000
C	7.509165000	1.339012000	0.313210000
C	7.376967000	-0.466946000	-1.390116000
H	7.002027000	-1.466853000	-1.655837000
H	7.038175000	0.234947000	-2.168137000
H	8.477949000	-0.504824000	-1.421294000
H	7.217416000	1.701422000	1.311095000
H	7.220346000	2.101548000	-0.426553000
H	8.607539000	1.265002000	0.297883000
C	7.379417000	-1.030119000	1.052976000
H	7.040722000	-0.741158000	2.060242000
H	7.007266000	-2.046304000	0.853552000
H	8.480485000	-1.075251000	1.063706000
C	-6.894487000	-0.024849000	0.001377000
C	-7.376911000	-0.467255000	1.390167000
C	-7.379479000	-1.029798000	-1.053066000

H	-7.007313000	-2.046034000	-0.853925000
H	-8.480548000	-1.074931000	-1.063748000
H	-7.040838000	-0.740576000	-2.060276000
H	-7.001974000	-1.467236000	1.655614000
H	-8.477892000	-0.505123000	1.421387000
H	-7.038073000	0.234432000	2.168356000
C	-7.509163000	1.339146000	-0.312681000
H	-7.220266000	2.101492000	0.427248000
H	-7.217486000	1.701797000	-1.310499000
H	-8.607537000	1.265156000	-0.297284000

6.2.3 Compound 1_3

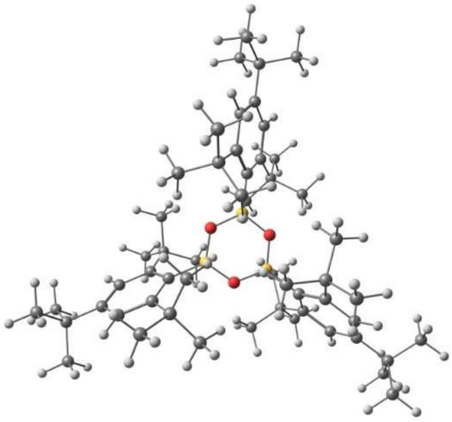

Compound 1_3 at PBE0-D3(BJ)/Def2-SVP(SMD: benzene)

O	0.327556000	-1.322591000	-0.000767000
O	-1.302147000	0.374998000	-0.002029000
O	0.983018000	0.937435000	0.019266000
B	1.356848000	-0.396100000	0.013469000
B	-1.014129000	-0.979629000	-0.009489000
B	-0.334260000	1.365276000	0.012806000
C	-2.166299000	-2.080200000	-0.024929000
C	-3.038828000	-2.218989000	-1.143903000
C	-2.366727000	-2.959212000	1.085516000
C	-4.053643000	-3.184275000	-1.117770000
C	-3.388904000	-3.908208000	1.035749000
C	-4.253573000	-4.044713000	-0.047125000
H	-4.711947000	-3.267691000	-1.976652000
H	-3.526648000	-4.573986000	1.884712000
C	-0.717782000	2.911770000	0.019922000
C	-1.391078000	3.500249000	1.130413000
C	-0.401996000	3.758213000	-1.088922000
C	-1.726992000	4.859761000	1.097871000
C	-0.753694000	5.108225000	-1.045650000
C	-1.421104000	5.690483000	0.028696000
H	-2.245735000	5.286150000	1.950561000
H	-0.502133000	5.741958000	-1.893017000
C	2.888051000	-0.837361000	0.020704000
C	3.469491000	-1.513283000	-1.097729000
C	3.728570000	-0.568431000	1.140505000
C	4.814326000	-1.884147000	-1.054120000
C	5.074136000	-0.956627000	1.108155000
C	5.646388000	-1.617649000	0.030078000
H	5.241900000	-2.402935000	-1.909073000
H	5.698114000	-0.735144000	1.968145000
C	-1.513956000	-2.932109000	2.371511000
C	-2.947292000	-1.359424000	-2.422908000
C	-1.789639000	2.728873000	2.406825000
C	0.317400000	3.274591000	-2.365797000

C	2.697931000	-1.870455000	-2.385733000
C	3.253475000	0.140369000	2.426855000
C	3.131327000	1.651635000	2.176596000
H	4.103770000	2.060661000	1.860558000
H	2.398620000	1.892343000	1.398630000
H	2.836076000	2.170238000	3.103339000
C	4.233135000	-0.036854000	3.596366000
H	3.785045000	0.397446000	4.503472000
H	4.445302000	-1.096873000	3.804793000
H	5.188607000	0.483575000	3.435337000
C	1.934810000	-0.463981000	2.903937000
H	1.577155000	0.045553000	3.810968000
H	1.129943000	-0.383319000	2.167473000
H	2.055273000	-1.533642000	3.130326000
C	1.785201000	-3.080344000	-2.131590000
H	1.263053000	-3.366949000	-3.059060000
H	2.386783000	-3.943698000	-1.806759000
H	1.033138000	-2.887180000	-1.359067000
C	1.910612000	-0.657811000	-2.877435000
H	1.184847000	-0.287648000	-2.147262000
H	2.587065000	0.178485000	-3.107691000
H	1.340216000	-0.903931000	-3.785338000
C	3.628282000	-2.255215000	-3.545118000
H	4.372642000	-1.472716000	-3.759021000
H	4.159778000	-3.201874000	-3.368471000
H	3.023259000	-2.396197000	-4.454104000
C	0.206013000	4.277697000	-3.523129000
H	0.647432000	3.828447000	-4.426167000
H	-0.839961000	4.531315000	-3.755002000
H	0.756192000	5.210827000	-3.332422000
C	-0.327891000	1.987764000	-2.875379000
H	0.186093000	1.623617000	-3.777301000
H	-0.293689000	1.169890000	-2.149535000
H	-1.386992000	2.153645000	-3.121687000
C	1.816649000	3.089129000	-2.083897000
H	2.343872000	2.784501000	-3.002736000
H	2.256580000	4.040651000	-1.746429000
H	2.010954000	2.337953000	-1.310729000
C	-0.614909000	1.888065000	2.901641000
H	-0.267362000	1.154574000	2.168181000
H	0.245871000	2.527395000	3.146999000
H	-0.893149000	1.319246000	3.801225000
C	-2.147374000	3.661041000	3.573901000
H	-1.339432000	4.373824000	3.800278000
H	-3.072671000	4.229032000	3.398073000
H	-2.316170000	3.052101000	4.475513000
C	-3.032299000	1.867841000	2.131130000
H	-3.349402000	1.348459000	3.050211000
H	-3.867606000	2.506516000	1.803717000
H	-2.860751000	1.116662000	1.352538000

C	-0.145167000	-3.578459000	2.106612000
H	-0.279039000	-4.618484000	1.770096000
H	0.429960000	-3.049920000	1.338743000
H	0.452590000	-3.601111000	3.032450000
C	-3.539070000	0.034233000	-2.159997000
H	-3.518125000	0.638826000	-3.081441000
H	-4.590199000	-0.056541000	-1.844220000
H	-3.000767000	0.579943000	-1.377431000
C	-2.156873000	-3.716700000	3.524272000
H	-2.189542000	-4.799870000	3.335842000
H	-1.552901000	-3.571057000	4.433216000
H	-3.178484000	-3.370615000	3.744836000
C	-3.734367000	-1.960372000	-3.596983000
H	-4.822158000	-1.943105000	-3.435189000
H	-3.538933000	-1.360362000	-4.499204000
H	-3.431577000	-2.996324000	-3.814279000
C	-1.499175000	-1.279685000	-2.900762000
H	-0.822384000	-0.840752000	-2.161818000
H	-1.110322000	-2.281444000	-3.135695000
H	-1.419648000	-0.655257000	-3.802995000
C	-1.376763000	-1.498011000	2.878175000
H	-0.755597000	-1.461635000	3.785404000
H	-0.906068000	-0.826460000	2.154054000
H	-2.363132000	-1.072489000	3.114712000
C	-1.780543000	7.176834000	-0.005987000
C	-2.511361000	7.622613000	1.260500000
C	-2.690833000	7.447346000	-1.212304000
H	-2.963232000	8.514311000	-1.258581000
H	-2.200245000	7.187761000	-2.162554000
H	-3.620673000	6.861064000	-1.145013000
H	-1.897350000	7.477087000	2.162730000
H	-3.459511000	7.080683000	1.400453000
H	-2.751489000	8.695155000	1.194661000
C	-0.493826000	8.003154000	-0.140813000
H	-0.727681000	9.079790000	-0.171318000
H	0.181148000	7.825150000	0.710988000
H	0.057247000	7.756153000	-1.060802000
C	-5.356437000	-5.104188000	-0.019171000
C	-6.283573000	-4.835048000	1.174509000
C	-4.717648000	-6.491899000	0.132132000
H	-5.494028000	-7.273765000	0.157735000
H	-4.042145000	-6.709981000	-0.709869000
H	-4.131913000	-6.574146000	1.060147000
H	-6.752523000	-3.841810000	1.094414000
H	-5.740939000	-4.872151000	2.131180000
H	-7.086666000	-5.588669000	1.217353000
C	-6.196992000	-5.094867000	-1.295945000
H	-6.702881000	-4.128853000	-1.448103000
H	-5.590233000	-5.308171000	-2.189559000
H	-6.977213000	-5.869353000	-1.234576000

C	7.113545000	-2.048876000	-0.004710000
C	7.811002000	-1.367131000	-1.190344000
C	7.187160000	-3.572715000	-0.175583000
H	6.688277000	-4.088657000	0.659728000
H	6.706143000	-3.904505000	-1.108130000
H	8.236806000	-3.907551000	-0.204991000
H	7.345799000	-1.637755000	-2.150239000
H	8.871064000	-1.665043000	-1.237912000
H	7.769232000	-0.270684000	-1.096003000
C	7.856266000	-1.669367000	1.276373000
H	7.416413000	-2.148983000	2.164491000
H	8.905389000	-1.996961000	1.210333000
H	7.860910000	-0.580868000	1.441880000

6.2.4 Compound 2

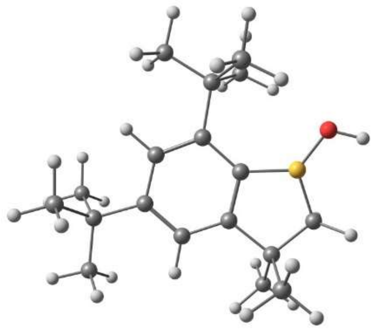

Compound 2 at PBE0-D3(BJ)/Def2-SVP(SMD: benzene)

B	-2.613029000	0.492595000	-0.077215000
O	-3.708354000	-0.300240000	0.075480000
C	-1.107904000	0.062295000	-0.039799000
C	-0.379008000	1.269404000	-0.046807000
C	-0.393583000	-1.167974000	-0.014869000
C	1.017195000	1.289667000	-0.029569000
C	0.999171000	-1.108308000	-0.002169000
C	1.729438000	0.094199000	-0.008371000
H	1.540549000	2.246262000	-0.032486000
H	1.563188000	-2.039673000	0.010230000
C	-1.226715000	2.539104000	-0.017032000
C	-2.653247000	2.048790000	-0.336499000
H	-2.863541000	2.181020000	-1.415806000
H	-4.528353000	0.205036000	0.037877000
H	-3.435518000	2.613018000	0.198233000
C	-1.184762000	3.122851000	1.401515000
H	-0.155379000	3.379100000	1.698071000
H	-1.794625000	4.038952000	1.465638000
H	-1.573152000	2.400408000	2.137022000
C	-1.947586000	-2.658182000	1.265820000
H	-1.314017000	-2.574001000	2.163128000
H	-2.728327000	-1.889453000	1.316518000
C	-0.115037000	-3.701383000	-0.027524000
H	0.538242000	-3.711786000	0.858672000
H	-0.676637000	-4.648382000	-0.029286000
H	0.521801000	-3.696665000	-0.925836000
C	-1.101145000	-2.529823000	-0.008920000
C	-1.987688000	-2.655045000	-1.256061000
H	-1.385393000	-2.561696000	-2.173743000
H	-2.479404000	-3.641454000	-1.274614000
H	-2.775709000	-1.892413000	-1.276939000
H	-2.443421000	-3.642468000	1.294736000
C	-0.737243000	3.586196000	-1.017257000
H	-1.418867000	4.452091000	-1.028733000
H	0.266813000	3.962847000	-0.765206000
H	-0.695745000	3.173017000	-2.037455000

C	3.258881000	0.040869000	0.006820000
C	3.726317000	-0.687423000	1.275126000
C	3.883457000	1.436073000	-0.005704000
H	3.604274000	2.003301000	-0.906999000
H	3.589057000	2.026187000	0.875847000
H	4.981308000	1.353119000	0.004920000
H	3.346102000	-1.718894000	1.321024000
H	3.382595000	-0.163627000	2.180825000
H	4.826874000	-0.736148000	1.306816000
C	3.751061000	-0.720528000	-1.232423000
H	3.423125000	-0.222376000	-2.158219000
H	4.852118000	-0.767066000	-1.242453000
H	3.374391000	-1.754011000	-1.257204000

6.2.5 *Compound 7 at PBE0-D3(BJ)/Def2-SVP(SMD: benzene)*

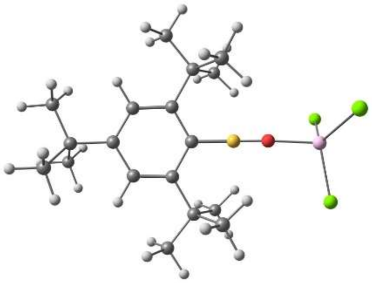

Compound 7 at PBE0-D3(BJ)/Def2-SVP(SMD: benzene)

B	-0.782343000	0.049834000	-0.015599000
O	-2.013794000	0.062801000	-0.016371000
C	0.711631000	0.035419000	-0.012376000
C	1.397785000	-1.211452000	-0.009267000
C	1.412756000	1.269040000	-0.010009000
C	2.786825000	-1.180315000	-0.003464000
C	2.806231000	1.223473000	-0.005237000
C	3.512679000	0.018564000	-0.001380000
H	3.334429000	-2.120859000	-0.000363000
H	3.360320000	2.157606000	-0.003830000
C	0.630073000	-2.540031000	-0.011508000
C	-0.244498000	-2.632301000	1.248227000
H	-0.779427000	-3.593883000	1.268660000
H	0.365976000	-2.555714000	2.160730000
H	-1.021141000	-1.853993000	1.298330000
C	-0.242549000	-2.629791000	-1.272762000
H	-0.772373000	-3.594049000	-1.298937000
H	-1.024044000	-1.856143000	-1.319058000
H	0.368377000	-2.545138000	-2.184250000
C	-0.207599000	2.712829000	-1.273402000
H	-0.714772000	3.689329000	-1.301963000
H	0.403481000	2.616656000	-2.183669000
C	1.623034000	3.799610000	-0.011871000
H	2.266336000	3.812703000	-0.904691000
H	1.041446000	4.733317000	-0.012750000
H	2.265890000	3.814381000	0.881216000
C	0.663093000	2.608077000	-0.011075000
C	-0.206740000	2.713532000	1.251849000
H	-0.718080000	3.687940000	1.277827000
H	-1.000166000	1.952102000	1.308618000
H	0.405994000	2.623058000	2.161597000
H	-1.005149000	1.955480000	-1.326122000
C	1.573154000	-3.744457000	-0.012058000
H	2.214703000	-3.768404000	0.881852000
H	0.978575000	-4.669954000	-0.014121000
H	2.217032000	-3.765608000	-0.904392000
C	5.040367000	-0.031148000	0.009508000

C	5.666374000	1.363259000	-0.009618000
C	5.526181000	-0.799577000	-1.228501000
H	5.157414000	-1.835948000	-1.244948000
H	5.194040000	-0.308343000	-2.156314000
H	6.626836000	-0.839245000	-1.240534000
H	5.380870000	1.956895000	0.872460000
H	5.387035000	1.928916000	-0.911898000
H	6.763247000	1.275190000	-0.003919000
C	5.504009000	-0.756203000	1.281808000
H	5.162133000	-0.228810000	2.185928000
H	5.125805000	-1.788208000	1.330779000
H	6.604102000	-0.803126000	1.311263000
Al	-3.855745000	-0.013674000	0.006192000
Cl	-4.222047000	-2.108168000	-0.050971000
Cl	-4.366358000	0.941039000	1.834723000
Cl	-4.420947000	1.051769000	-1.743294000

6.2.6 Al_2Cl_6

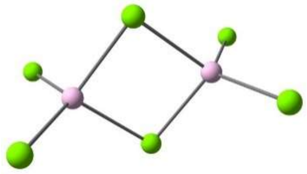

Al_2Cl_6 at PBE0-D3(BJ)/Def2-SVP(SMD: benzene)

Al	-1.597003000	-0.000002000	0.000066000
Cl	-2.598417000	1.829358000	-0.000012000
Cl	0.000002000	0.000087000	-1.626097000
Cl	-2.598426000	-1.829368000	-0.000030000
Al	1.597007000	-0.000001000	-0.000059000
Cl	2.598412000	1.829362000	0.000070000
Cl	-0.000002000	-0.000068000	1.626114000
Cl	2.598427000	-1.829368000	-0.000050000

6.2.7 [Mes*CO]+ at PBE0-D3(BJ)/Def2-SVP(SMD: benzene)

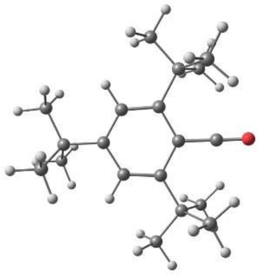

[Mes*CO]+ at PBE0-D3(BJ)/Def2-SVP(SMD: benzene)

C	2.465477000	0.120053000	0.009387000
O	3.599756000	0.174788000	0.015968000
C	1.096626000	0.046572000	0.003096000
C	0.482223000	-1.258710000	0.003442000
C	0.342173000	1.279818000	-0.001324000
C	-0.901935000	-1.273659000	0.005994000
C	-1.030597000	1.141767000	-0.002930000
C	-1.679066000	-0.106688000	0.003575000
H	-1.405114000	-2.235809000	0.007893000
H	-1.640859000	2.042422000	-0.008703000
C	1.292250000	-2.556754000	-0.003181000
C	0.375461000	-3.783568000	-0.001785000
H	0.997183000	-4.690088000	-0.005673000
H	-0.259508000	-3.826310000	0.895496000
H	-0.266830000	-3.823531000	-0.893920000
C	2.147329000	-2.627110000	-1.280664000
H	1.512696000	-2.587054000	-2.178512000
H	2.901139000	-1.831139000	-1.368026000
H	2.698211000	-3.579175000	-1.298048000
C	1.871629000	2.815127000	-1.268169000
H	1.258554000	2.705834000	-2.175270000
H	2.319171000	3.819959000	-1.280419000
C	1.853447000	2.822822000	1.274081000
H	2.683902000	2.108344000	1.366224000
H	2.304629000	3.826062000	1.284557000
H	1.225937000	2.723705000	2.172392000
C	1.011614000	2.655486000	-0.002743000
C	-0.023805000	3.783044000	-0.014420000
H	0.505728000	4.746558000	-0.014910000
H	-0.656995000	3.759817000	-0.913914000
H	-0.669300000	3.767577000	0.876408000
H	2.706394000	2.103807000	-1.341575000
C	2.162367000	-2.637038000	1.263304000
H	2.916909000	-1.841916000	1.348816000
H	1.538200000	-2.604463000	2.168776000
H	2.713680000	-3.588925000	1.267155000
C	-3.199306000	-0.152388000	0.002622000
C	-3.741536000	-1.580235000	0.027262000

C	-3.704776000	0.545898000	-1.271704000
H	-3.392229000	1.599165000	-1.325585000
H	-3.343467000	0.034115000	-2.176768000
H	-4.805204000	0.525866000	-1.286779000
H	-3.433153000	-2.157178000	-0.858169000
H	-4.840820000	-1.552682000	0.029601000
H	-3.427406000	-2.127981000	0.929071000
C	-3.713659000	0.591398000	1.247129000
H	-3.403121000	1.646177000	1.265884000
H	-4.814062000	0.570826000	1.256109000
H	-3.357804000	0.112710000	2.172309000

6.2.8 *$Et_2O·BF_3$*

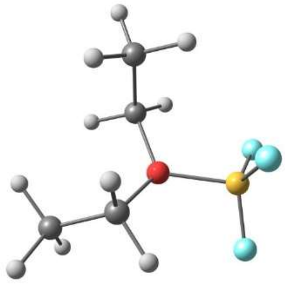

$Et_2O·BF_3$ at PBE0-D3(BJ)/Def2-SVP(SMD: benzene)

H	-1.782924000	1.796888000	1.144892000
O	-0.306163000	-0.105828000	-0.313383000
C	-1.157888000	-0.939624000	0.507906000
C	-2.421134000	-1.339321000	-0.205532000
H	-1.355132000	-0.415740000	1.455330000
H	-0.537691000	-1.816921000	0.723843000
H	-2.194056000	-1.795925000	-1.180447000
H	-2.943106000	-2.089512000	0.407229000
H	-3.117214000	-0.501108000	-0.355733000
C	-0.815097000	1.189607000	-0.711660000
C	-1.007827000	2.138274000	0.442745000
H	-1.747791000	1.006930000	-1.262784000
H	-0.069360000	1.561712000	-1.422489000
H	-0.070689000	2.287305000	0.996968000
H	-1.330499000	3.112053000	0.044391000
B	1.281205000	-0.241641000	-0.001513000
F	1.538523000	-1.567215000	-0.188115000
F	1.874732000	0.580302000	-0.911230000
F	1.431568000	0.166417000	1.295198000

6.2.9 PhN$_3$

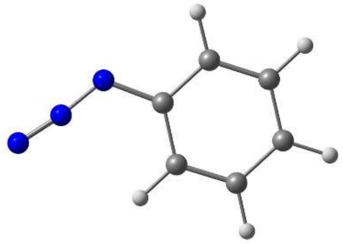

PhN$_3$ at PBE0-D3(BJ)/Def2-SVP(SMD: benzene)

N	1.466599000	-0.864439000	-0.000017000
N	2.411122000	-0.091128000	-0.000024000
N	3.361843000	0.523776000	0.000020000
C	0.151694000	-0.364629000	-0.000004000
C	-0.879454000	-1.309778000	-0.000013000
C	-0.149877000	1.003433000	0.000013000
C	-2.204127000	-0.885890000	-0.000006000
C	-1.479980000	1.413669000	0.000019000
C	-2.511759000	0.475109000	0.000010000
H	-0.621081000	-2.370453000	-0.000026000
H	0.654155000	1.744498000	0.000021000
H	-3.005396000	-1.628873000	-0.000013000
H	-1.710351000	2.481897000	0.000032000
H	-3.553254000	0.803986000	0.000015000

6.2.10 TS1

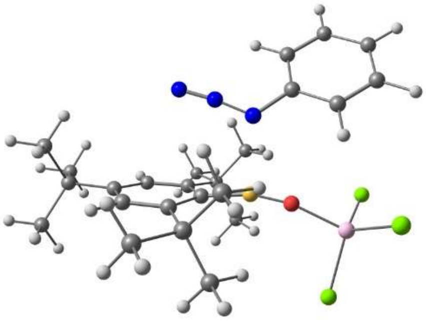

TS1 at PBE0-D3(BJ)/Def2-SVP(SMD: benzene)

B	-0.362806000	-0.493521000	-0.021873000
O	-1.552179000	-0.838634000	0.063080000
C	1.139954000	-0.425230000	0.108208000
C	1.959957000	-0.965802000	-0.921346000
C	1.710687000	0.242037000	1.223744000
C	3.327533000	-0.720320000	-0.854870000
C	3.092002000	0.446464000	1.225048000
C	3.913528000	0.007243000	0.187126000
H	3.971109000	-1.108864000	-1.641596000
H	3.541294000	0.968851000	2.064583000
C	1.378438000	-1.865005000	-2.023631000
C	0.804986000	-3.129060000	-1.361216000
H	0.393966000	-3.806338000	-2.125996000
H	1.587254000	-3.671314000	-0.808330000
H	-0.007402000	-2.912636000	-0.651160000
C	0.277257000	-1.148176000	-2.814278000
H	0.639033000	-0.203729000	-3.248145000
H	-0.067215000	-1.792304000	-3.637553000
H	-0.619276000	-0.927154000	-2.217604000
C	-0.259909000	1.629088000	2.020267000
H	-1.017065000	1.176970000	1.364328000
H	-0.813458000	1.961149000	2.911307000
C	1.696475000	1.376153000	3.499986000
H	2.149358000	2.305538000	3.121544000
H	1.047212000	1.646147000	4.346054000
H	2.497966000	0.734943000	3.895940000
C	0.860307000	0.665911000	2.432946000
C	0.259186000	-0.596302000	3.073082000
H	-0.368142000	-0.324460000	3.935948000
H	-0.374821000	-1.171592000	2.383393000
H	1.053670000	-1.272379000	3.424310000
H	0.145987000	2.523494000	1.523053000
C	2.448510000	-2.303642000	-3.025772000
H	3.250633000	-2.886905000	-2.549427000
H	1.987231000	-2.947729000	-3.789158000
H	2.903285000	-1.447175000	-3.547101000
C	5.416032000	0.285370000	0.154840000

C	5.885132000	1.074649000	1.376705000
C	5.731788000	1.104334000	-1.105698000
H	5.466158000	0.561156000	-2.025038000
H	5.178836000	2.056636000	-1.107477000
H	6.808379000	1.333334000	-1.152398000
H	5.705622000	0.527917000	2.315304000
H	5.390244000	2.055391000	1.448602000
H	6.968094000	1.257449000	1.305422000
C	6.180034000	-1.045674000	0.111362000
H	5.959773000	-1.658502000	0.999339000
H	5.926539000	-1.640342000	-0.778990000
H	7.265594000	-0.860065000	0.087403000
Al	-3.118988000	-1.624958000	0.576199000
Cl	-4.193572000	-1.885906000	-1.248678000
Cl	-2.489161000	-3.451004000	1.475038000
Cl	-3.989308000	-0.237456000	1.932692000
N	-0.606286000	1.533041000	-1.211474000
C	-1.796958000	2.300424000	-1.150940000
C	-1.791035000	3.664666000	-0.841752000
C	-2.998863000	1.626664000	-1.371570000
C	-3.000070000	4.348168000	-0.752082000
C	-4.200426000	2.320120000	-1.265467000
C	-4.207151000	3.679375000	-0.956579000
H	-0.848097000	4.189659000	-0.667851000
H	-2.994192000	0.566898000	-1.625797000
H	-2.996266000	5.413850000	-0.511194000
H	-5.137490000	1.782036000	-1.424807000
H	-5.153050000	4.218986000	-0.873270000
N	0.475827000	2.105315000	-1.299613000
N	1.530156000	2.502742000	-1.366352000

6.2.11 IM1

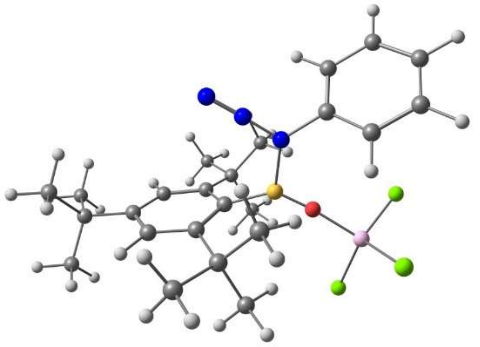

IM1 at PBE0-D3(BJ)/Def2-SVP(SMD: benzene)

B	-0.421474000	0.093656000	-0.094210000
O	-1.454467000	-0.549096000	0.319242000
C	1.128565000	-0.060516000	0.039506000
C	1.869420000	-0.750494000	-0.958933000
C	1.811939000	0.591706000	1.097378000
C	3.260781000	-0.652317000	-0.941388000
C	3.210181000	0.649776000	1.056706000
C	3.954650000	0.068723000	0.033263000
H	3.835451000	-1.162796000	-1.711878000
H	3.736078000	1.163618000	1.856085000
C	1.196214000	-1.705140000	-1.963974000
C	0.721147000	-2.937140000	-1.174865000
H	0.201480000	-3.645916000	-1.839135000
H	1.575393000	-3.458034000	-0.716058000
H	0.028189000	-2.667860000	-0.365501000
C	-0.005626000	-1.075184000	-2.676861000
H	0.249786000	-0.117278000	-3.157236000
H	-0.365111000	-1.753625000	-3.464755000
H	-0.869086000	-0.938567000	-2.012427000
C	-0.104080000	2.037732000	2.009054000
H	-0.959480000	1.504003000	1.571661000
H	-0.496400000	2.487579000	2.933340000
C	2.008855000	1.920826000	3.265719000
H	2.439367000	2.799414000	2.759509000
H	1.436404000	2.283183000	4.132707000
H	2.834146000	1.310648000	3.660153000
C	1.079293000	1.121435000	2.347020000
C	0.566872000	-0.096787000	3.133239000
H	0.033337000	0.227369000	4.041194000
H	-0.122424000	-0.714803000	2.541669000
H	1.404554000	-0.741004000	3.441392000
H	0.197358000	2.864232000	1.345338000
C	2.164661000	-2.178265000	-3.052206000
H	3.006174000	-2.755835000	-2.643001000
H	1.630923000	-2.842133000	-3.748720000
H	2.572683000	-1.338792000	-3.637316000

C	5.480104000	0.162749000	-0.036226000
C	6.060850000	0.986933000	1.112742000
C	5.879217000	0.827157000	-1.361507000
H	5.523984000	0.253857000	-2.231114000
H	5.461821000	1.843722000	-1.436370000
H	6.975726000	0.903684000	-1.438558000
H	5.839784000	0.539166000	2.093847000
H	5.679759000	2.019939000	1.112696000
H	7.156120000	1.039552000	1.015780000
C	6.077412000	-1.250111000	0.029634000
H	5.795780000	-1.755086000	0.966764000
H	5.736907000	-1.880513000	-0.805505000
H	7.177422000	-1.204553000	-0.015349000
Al	-2.793524000	-1.601010000	0.823773000
Cl	-3.528468000	-2.441848000	-1.017583000
Cl	-1.994384000	-3.101538000	2.118725000
Cl	-4.212500000	-0.325390000	1.788986000
N	-0.809434000	1.407212000	-0.945456000
C	-2.142049000	1.949769000	-1.100041000
C	-2.392725000	3.269129000	-0.726920000
C	-3.147420000	1.110607000	-1.567760000
C	-3.690247000	3.759921000	-0.839193000
C	-4.443433000	1.612214000	-1.651947000
C	-4.715224000	2.931331000	-1.295122000
H	-1.591170000	3.902957000	-0.340114000
H	-2.939662000	0.075483000	-1.839301000
H	-3.901744000	4.791696000	-0.550723000
H	-5.243972000	0.953288000	-1.994036000
H	-5.735248000	3.315513000	-1.364505000
N	0.133533000	2.103710000	-1.390491000
N	1.001534000	2.694846000	-1.770988000

6.2.12 TS2

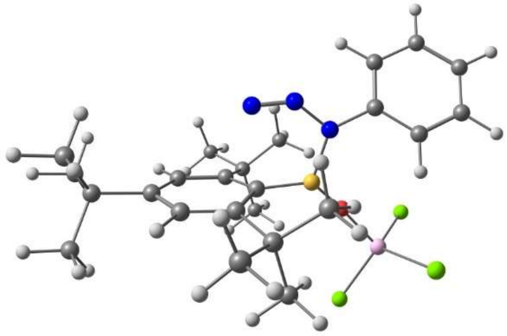

TS2 at PBE0-D3(BJ)/Def2-SVP(SMD: benzene)

B	-0.504531000	0.191379000	-0.108016000
O	-1.456711000	-0.596223000	0.262378000
C	1.073189000	0.082165000	-0.033558000
C	1.819578000	-0.510931000	-1.111280000
C	1.776868000	0.546976000	1.128388000
C	3.206813000	-0.458544000	-1.062939000
C	3.171568000	0.564051000	1.101728000
C	3.907132000	0.105697000	0.009621000
H	3.779064000	-0.880152000	-1.886420000
H	3.705455000	0.943493000	1.967501000
C	1.137691000	-1.310959000	-2.231111000
C	0.700563000	-2.648868000	-1.606839000
H	0.154719000	-3.255157000	-2.346816000
H	1.574610000	-3.224299000	-1.265836000
H	0.040451000	-2.507490000	-0.739923000
C	-0.089473000	-0.607489000	-2.822446000
H	0.136922000	0.417644000	-3.153364000
H	-0.441273000	-1.167755000	-3.700926000
H	-0.946744000	-0.595704000	-2.137069000
C	-0.183639000	1.779075000	2.252842000
H	-1.026625000	1.257095000	1.780933000
H	-0.558807000	2.086101000	3.240012000
C	1.975408000	1.617946000	3.429461000
H	2.366236000	2.560957000	3.015594000
H	1.405799000	1.862317000	4.338193000
H	2.826708000	0.998621000	3.746260000
C	1.055611000	0.894295000	2.440378000
C	0.626954000	-0.445444000	3.066511000
H	0.089569000	-0.266938000	4.011471000
H	-0.037356000	-1.020152000	2.406418000
H	1.503756000	-1.074031000	3.284530000
H	0.046487000	2.697964000	1.692269000
C	2.090992000	-1.606849000	-3.393110000
H	2.940907000	-2.236809000	-3.093579000
H	1.547326000	-2.159696000	-4.173381000
H	2.484223000	-0.684336000	-3.848817000

C	5.431917000	0.168992000	-0.039368000
C	6.025784000	0.819355000	1.209686000
C	5.849784000	0.993768000	-1.266028000
H	5.488997000	0.547435000	-2.204712000
H	5.454075000	2.019718000	-1.207182000
H	6.947957000	1.054974000	-1.326161000
H	5.792883000	0.250061000	2.122656000
H	5.668307000	1.851652000	1.345953000
H	7.121875000	0.859250000	1.119877000
C	5.990363000	-1.256644000	-0.161576000
H	5.689856000	-1.875328000	0.698217000
H	5.643392000	-1.760174000	-1.076156000
H	7.091095000	-1.230647000	-0.193857000
Al	-2.680560000	-1.804021000	0.686397000
Cl	-3.497863000	-2.411173000	-1.210586000
Cl	-1.686162000	-3.421836000	1.674562000
Cl	-4.100826000	-0.807881000	1.935657000
N	-0.926377000	1.560828000	-0.747270000
C	-2.230209000	2.121334000	-0.875165000
C	-2.425715000	3.478552000	-0.610162000
C	-3.294603000	1.297685000	-1.235891000
C	-3.704789000	4.014847000	-0.714439000
C	-4.573214000	1.844361000	-1.312541000
C	-4.782566000	3.197437000	-1.056328000
H	-1.579493000	4.107391000	-0.321937000
H	-3.140295000	0.240677000	-1.452282000
H	-3.860973000	5.076941000	-0.512608000
H	-5.409443000	1.194460000	-1.578748000
H	-5.788482000	3.618043000	-1.121422000
N	0.092444000	2.287110000	-1.039226000
N	1.236631000	2.204823000	-0.976297000

6.2.13 IM2

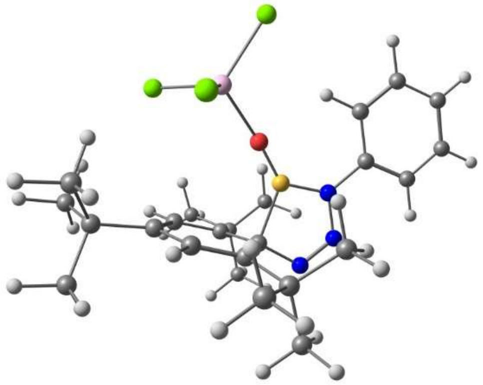

IM2 at PBE0-D3(BJ)/Def2-SVP(SMD: benzene)

Cl	1.634138000	2.725322000	1.645747000
Cl	-1.401463000	3.994887000	0.391620000
Cl	1.017552000	2.790758000	-1.905693000
Al	0.188291000	2.599834000	0.062014000
O	-0.495991000	0.963174000	0.133489000
N	-2.148222000	-0.915808000	0.009484000
N	-2.065027000	-2.255904000	-0.085110000
N	-0.910265000	-2.711623000	-0.121968000
C	0.129069000	-1.697906000	-0.056790000
C	0.833774000	-1.382338000	-1.306603000
C	2.038369000	-0.714445000	-1.197849000
H	2.555814000	-0.422043000	-2.106675000
C	2.672020000	-0.462994000	0.028361000
C	2.032690000	-0.861530000	1.207177000
H	2.535664000	-0.676516000	2.149327000
C	0.828068000	-1.544693000	1.222746000
C	4.088537000	0.096637000	0.036555000
C	4.989298000	-0.989169000	-0.583414000
H	4.720561000	-1.202088000	-1.629005000
H	4.931627000	-1.931721000	-0.016935000
H	6.035953000	-0.647369000	-0.569649000
C	4.178682000	1.375862000	-0.804568000
H	3.854898000	1.223655000	-1.843574000
H	5.225048000	1.716833000	-0.830045000
H	3.565137000	2.177938000	-0.373379000
C	4.587832000	0.401832000	1.448359000
H	3.943343000	1.137259000	1.951234000
H	5.599948000	0.828791000	1.390475000
H	4.653038000	-0.501905000	2.073685000
C	0.302858000	-2.114860000	2.548372000
C	-1.128646000	-1.658628000	2.877881000
H	-1.233327000	-0.564174000	2.818819000
H	-1.357727000	-1.949999000	3.913372000
H	-1.890942000	-2.127029000	2.247119000
C	1.182892000	-1.673600000	3.723967000

H	2.220058000	-2.028897000	3.634899000
H	0.774111000	-2.105189000	4.648889000
H	1.193344000	-0.579323000	3.843881000
C	0.362716000	-3.650394000	2.459969000
H	0.008226000	-4.082269000	3.408823000
H	1.396272000	-3.993196000	2.297193000
H	-0.262408000	-4.043434000	1.647126000
C	-3.443552000	-0.339232000	0.035065000
C	-4.575071000	-1.137963000	-0.170432000
H	-4.459033000	-2.206622000	-0.347653000
C	-5.839019000	-0.556183000	-0.144637000
H	-6.717246000	-1.186245000	-0.305816000
C	-5.990318000	0.811931000	0.079050000
H	-6.985601000	1.261724000	0.094293000
C	-4.858207000	1.597985000	0.280775000
H	-4.951076000	2.672536000	0.455108000
C	-3.586455000	1.031617000	0.263670000
H	-2.715969000	1.665912000	0.421813000
B	-0.848355000	-0.264281000	0.049745000
C	0.299416000	-1.762858000	-2.694608000
C	-1.113632000	-1.213679000	-2.953136000
C	0.307908000	-3.296807000	-2.814592000
H	-0.057508000	-3.584345000	-3.812690000
H	1.329432000	-3.691981000	-2.701760000
H	-0.330629000	-3.777849000	-2.061997000
H	-1.891698000	-1.731836000	-2.383426000
H	-1.356459000	-1.357616000	-4.016235000
H	-1.172530000	-0.133252000	-2.749902000
C	1.196356000	-1.200264000	-3.803357000
H	0.781229000	-1.501753000	-4.775822000
H	1.234465000	-0.100666000	-3.784885000
H	2.223139000	-1.592194000	-3.756498000

6.2.14 TS3

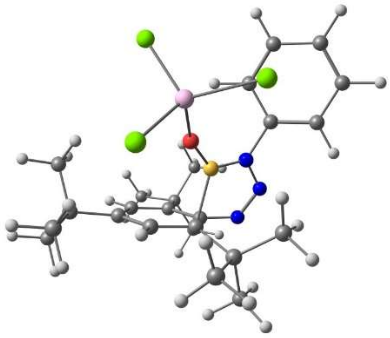

TS3 at PBE0-D3(BJ)/Def2-SVP(SMD: benzene)

Cl	2.067725000	2.533699000	-2.448869000
Cl	3.645954000	1.580354000	0.646541000
Cl	0.853919000	3.735488000	0.680891000
Al	1.843833000	2.133369000	-0.362101000
O	0.748227000	0.729841000	-0.258194000
N	0.961034000	-1.682345000	0.168134000
N	0.400961000	-2.762924000	0.750550000
N	-0.808571000	-2.669915000	1.032204000
C	-1.453274000	-1.483738000	0.666353000
C	-1.099298000	-0.274140000	1.373810000
C	-1.766867000	0.919107000	0.945516000
H	-1.534026000	1.842529000	1.466056000
C	-2.620555000	0.957337000	-0.134700000
C	-2.891153000	-0.257228000	-0.794335000
H	-3.564930000	-0.227311000	-1.649221000
C	-2.340086000	-1.484368000	-0.444007000
C	-3.245036000	2.247982000	-0.657949000
C	-4.764485000	2.074350000	-0.781542000
H	-5.221122000	1.850080000	0.195296000
H	-5.043275000	1.270012000	-1.479226000
H	-5.217018000	3.003550000	-1.160509000
C	-2.959862000	3.431388000	0.264752000
H	-3.356521000	3.264677000	1.278910000
H	-3.446828000	4.333489000	-0.134764000
H	-1.883767000	3.646620000	0.339577000
C	-2.636287000	2.535272000	-2.039377000
H	-1.543633000	2.652542000	-1.976969000
H	-3.054814000	3.466743000	-2.451467000
H	-2.849393000	1.727659000	-2.757269000
C	-2.671000000	-2.756504000	-1.238813000
C	-1.416995000	-3.259613000	-1.972854000
H	-0.969802000	-2.465330000	-2.590774000
H	-1.693693000	-4.088418000	-2.642691000
H	-0.647534000	-3.642146000	-1.291054000
C	-3.742782000	-2.477027000	-2.296015000
H	-4.675615000	-2.093482000	-1.854263000

H	-3.987470000	-3.417055000	-2.811696000
H	-3.400178000	-1.766125000	-3.063570000
C	-3.217201000	-3.843048000	-0.300475000
H	-3.464034000	-4.742459000	-0.885483000
H	-4.137720000	-3.502718000	0.199231000
H	-2.491025000	-4.128735000	0.471453000
C	2.297161000	-1.845676000	-0.300773000
C	3.223493000	-2.571070000	0.449604000
H	2.917890000	-3.032947000	1.389406000
C	4.530568000	-2.690734000	-0.013773000
H	5.258428000	-3.255265000	0.573751000
C	4.916230000	-2.079711000	-1.206571000
H	5.948005000	-2.162371000	-1.555620000
C	3.982814000	-1.359016000	-1.948520000
H	4.274983000	-0.869999000	-2.880381000
C	2.667957000	-1.248563000	-1.505932000
H	1.937833000	-0.682942000	-2.086315000
B	0.330681000	-0.378559000	0.234469000
C	-0.557135000	-0.295921000	2.859978000
C	0.873582000	-0.820633000	3.051667000
C	-1.534044000	-1.165739000	3.663029000
H	-1.269370000	-1.126182000	4.731015000
H	-2.567358000	-0.800673000	3.555587000
H	-1.503078000	-2.217731000	3.344874000
H	0.985608000	-1.883655000	2.809551000
H	1.135829000	-0.710667000	4.113779000
H	1.615622000	-0.238278000	2.483181000
C	-0.572850000	1.121236000	3.441884000
H	-0.171917000	1.080053000	4.464745000
H	0.058356000	1.818440000	2.869872000
H	-1.587964000	1.538817000	3.507990000

6.2.15 IM3

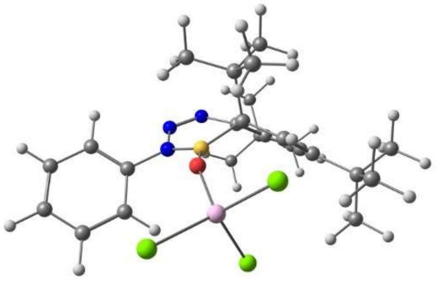

IM3 at PBE0-D3(BJ)/Def2-SVP(SMD: benzene)

Cl	1.245905000	2.182264000	-2.121722000
Cl	4.321598000	2.025238000	-0.322073000
Cl	1.501625000	3.623419000	1.123144000
Al	2.194521000	2.062158000	-0.185367000
O	1.604491000	0.523996000	0.470181000
N	1.079433000	-1.817565000	0.059196000
N	0.269342000	-2.852599000	0.287906000
N	-0.967961000	-2.660471000	0.426707000
C	-1.512663000	-1.401361000	0.336621000
C	-0.813169000	-0.275984000	0.950210000
C	-1.313866000	1.043419000	0.605813000
H	-0.765211000	1.899160000	0.998132000
C	-2.415240000	1.237118000	-0.185936000
C	-3.104341000	0.079006000	-0.621809000
H	-4.007631000	0.234843000	-1.211949000
C	-2.704346000	-1.238339000	-0.398315000
C	-2.930638000	2.610056000	-0.607783000
C	-4.375619000	2.776311000	-0.119128000
H	-4.432459000	2.718792000	0.979356000
H	-5.051835000	2.012369000	-0.533453000
H	-4.765920000	3.758900000	-0.426311000
C	-2.077958000	3.734343000	-0.025574000
H	-2.087844000	3.733479000	1.075488000
H	-2.474738000	4.705956000	-0.355852000
H	-1.031706000	3.674618000	-0.357434000
C	-2.878719000	2.695170000	-2.139988000
H	-1.846387000	2.574701000	-2.501619000
H	-3.246318000	3.676853000	-2.476839000
H	-3.503246000	1.925776000	-2.620807000
C	-3.499098000	-2.417840000	-0.976637000
C	-2.655541000	-3.150277000	-2.031124000
H	-2.340567000	-2.463909000	-2.832919000
H	-3.253035000	-3.952954000	-2.491004000
H	-1.758488000	-3.607458000	-1.593955000
C	-4.784978000	-1.939076000	-1.655845000
H	-5.445059000	-1.391482000	-0.964669000
H	-5.345146000	-2.814456000	-2.015745000
H	-4.586988000	-1.300277000	-2.530716000

C	-3.897732000	-3.378872000	0.153908000
H	-4.489645000	-4.209610000	-0.260717000
H	-4.520386000	-2.866721000	0.904630000
H	-3.019076000	-3.803537000	0.655270000
C	2.407540000	-2.173725000	-0.319939000
C	3.052459000	-3.244013000	0.303551000
H	2.528862000	-3.815061000	1.071748000
C	4.355170000	-3.564703000	-0.066488000
H	4.862419000	-4.400132000	0.422001000
C	5.015866000	-2.817103000	-1.041676000
H	6.042994000	-3.064977000	-1.319229000
C	4.363038000	-1.751779000	-1.658585000
H	4.872038000	-1.156883000	-2.419963000
C	3.054142000	-1.431546000	-1.308423000
H	2.535532000	-0.609404000	-1.804658000
B	0.735475000	-0.440931000	0.430961000
C	-0.637852000	-0.412038000	2.596525000
C	0.356874000	-1.484735000	3.034188000
C	-2.031136000	-0.759206000	3.123444000
H	-2.024441000	-0.702518000	4.223395000
H	-2.791137000	-0.053190000	2.755265000
H	-2.332222000	-1.779102000	2.841680000
H	0.103347000	-2.489151000	2.672507000
H	0.331707000	-1.530746000	4.133348000
H	1.391852000	-1.241664000	2.750028000
C	-0.180541000	0.923781000	3.173906000
H	0.062198000	0.773145000	4.236410000
H	0.719359000	1.313251000	2.678514000
H	-0.963667000	1.693173000	3.122488000

6.2.16 Compound 9

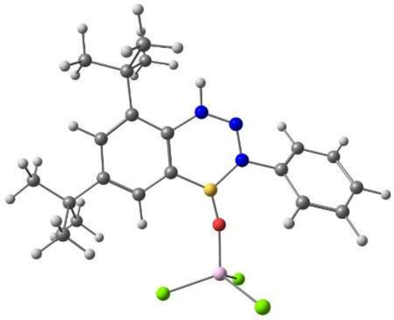

Compound 9 at PBE0-D3(BJ)/Def2-SVP(SMD: benzene)

Cl	-3.835539000	2.001369000	-1.737664000
Cl	-1.213632000	3.661073000	-0.014866000
Cl	-3.652209000	1.867101000	1.824845000
Al	-2.556694000	1.967463000	-0.025200000
O	-1.597720000	0.488586000	-0.091737000
N	-1.017850000	-1.877329000	0.075411000
N	-0.148390000	-2.825696000	0.120130000
N	1.097364000	-2.544251000	0.103413000
C	1.721231000	-1.300038000	0.045173000
C	0.870234000	-0.183734000	-0.008549000
C	1.426481000	1.093800000	-0.064210000
H	0.746784000	1.948200000	-0.103960000
C	2.807712000	1.272816000	-0.059292000
C	3.617487000	0.123174000	-0.010975000
H	4.695091000	0.262526000	-0.012854000
C	3.132818000	-1.183949000	0.039481000
C	3.386915000	2.685501000	-0.099136000
C	4.914994000	2.687132000	-0.107572000
H	5.335188000	2.216563000	0.795215000
H	5.282101000	3.724081000	-0.135707000
H	5.325113000	2.171068000	-0.989990000
C	2.883371000	3.393554000	-1.365803000
H	3.293859000	4.414421000	-1.415785000
H	1.786745000	3.477829000	-1.379722000
H	3.196977000	2.856226000	-2.274630000
C	2.896970000	3.450685000	1.139929000
H	1.800006000	3.529340000	1.164722000
H	3.303145000	4.474576000	1.136727000
H	3.225402000	2.957598000	2.068416000
C	4.089546000	-2.386220000	0.080754000
C	3.903729000	-3.181576000	1.385642000
H	4.625056000	-4.012290000	1.422957000
H	2.904941000	-3.624433000	1.520302000
H	4.084120000	-2.537475000	2.259434000
C	5.553263000	-1.935026000	0.050249000
H	6.204001000	-2.821535000	0.079097000
H	5.812214000	-1.308604000	0.916761000

H	5.796242000	-1.378565000	-0.867251000
C	3.880826000	-3.281074000	-1.154608000
H	4.597694000	-4.116347000	-1.137040000
H	4.051434000	-2.708678000	-2.078880000
H	2.877220000	-3.725809000	-1.238727000
C	-2.373988000	-2.336491000	0.027818000
C	-2.694857000	-3.442620000	-0.761602000
H	-1.914116000	-3.940719000	-1.339166000
C	-4.012443000	-3.885795000	-0.811106000
H	-4.268335000	-4.746926000	-1.432605000
C	-5.002978000	-3.222895000	-0.086235000
H	-6.039175000	-3.564980000	-0.137690000
C	-4.670535000	-2.117853000	0.695379000
H	-5.439133000	-1.586324000	1.260654000
C	-3.354749000	-1.668670000	0.761580000
H	-3.106001000	-0.799918000	1.370784000
B	-0.656039000	-0.404035000	-0.009720000
H	1.671850000	-3.380979000	0.155510000

6.2.17 Isobutylene

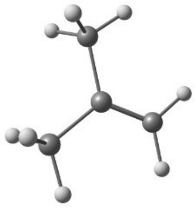

Isobutylene at PBE0-D3(BJ)/Def2-SVP(SMD: benzene)

C	-0.000001000	1.459719000	-0.000071000
C	0.000000000	0.121111000	-0.000012000
H	0.933518000	2.030955000	-0.000134000
H	-0.933522000	2.030952000	-0.000062000
C	-1.269068000	-0.677094000	0.000060000
H	-2.162145000	-0.036008000	0.000103000
H	-1.318315000	-1.337442000	-0.882992000
H	-1.318224000	-1.337436000	0.883121000
C	1.269069000	-0.677092000	0.000006000
H	1.318182000	-1.337617000	-0.882919000
H	2.162146000	-0.036005000	-0.000208000
H	1.318361000	-1.337257000	0.883194000

6.2.18 9B-1

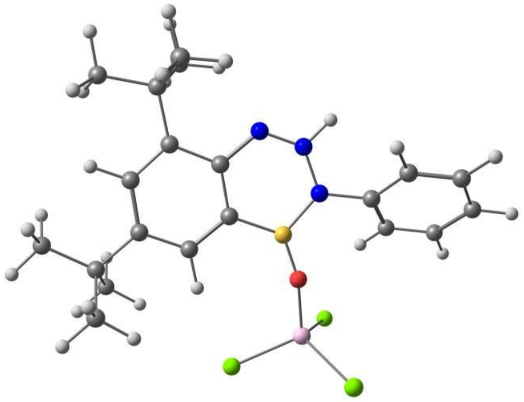

9B-1 at PBE0-D3(BJ)/Def2-SVP(SMD: benzene)

Cl	3.524850000	1.800613000	1.941186000
Cl	1.179162000	3.690956000	0.070538000
Cl	3.924743000	2.170486000	-1.583326000
Al	2.537067000	2.008531000	0.036006000
O	1.604735000	0.532747000	-0.176184000
N	1.045266000	-1.811679000	0.040941000
N	0.081898000	-2.728521000	0.154716000
N	-1.162657000	-2.569248000	0.164807000
C	-1.704066000	-1.320707000	0.052664000
C	-0.862143000	-0.182315000	-0.046346000
C	-1.426816000	1.083558000	-0.137731000
H	-0.758113000	1.944856000	-0.203124000
C	-2.813834000	1.250591000	-0.130503000
C	-3.621885000	0.100134000	-0.038246000
H	-4.699829000	0.236839000	-0.036015000
C	-3.126788000	-1.196760000	0.054853000
C	-3.399098000	2.656804000	-0.205323000
C	-4.926478000	2.652903000	-0.248308000
H	-5.313901000	2.115547000	-1.128180000
H	-5.295177000	3.687908000	-0.307510000
H	-5.366261000	2.201150000	0.654431000
C	-2.936660000	3.437364000	1.035856000
H	-3.345256000	4.459914000	1.009346000
H	-1.840640000	3.517592000	1.083707000
H	-3.285826000	2.955396000	1.962485000
C	-2.869377000	3.351568000	-1.469306000
H	-1.774226000	3.450761000	-1.456734000
H	-3.291508000	4.366091000	-1.542553000
H	-3.154106000	2.798664000	-2.378197000
C	-4.069431000	-2.401120000	0.154935000
C	-5.537996000	-1.969607000	0.109912000
H	-5.805317000	-1.309149000	0.948805000
H	-6.176737000	-2.862566000	0.180826000
H	-5.793653000	-1.457867000	-0.830625000
C	-3.843137000	-3.133997000	1.487804000

H	-2.827111000	-3.541581000	1.567186000
H	-4.554220000	-3.970391000	1.577417000
H	-4.011128000	-2.457807000	2.340627000
C	-3.833602000	-3.354944000	-1.027935000
H	-2.819738000	-3.775577000	-1.022554000
H	-3.987615000	-2.836893000	-1.987529000
H	-4.549040000	-4.190946000	-0.979397000
C	2.368002000	-2.326489000	-0.048398000
C	3.386728000	-1.721341000	0.691418000
H	3.174811000	-0.858154000	1.324005000
C	4.680751000	-2.221703000	0.593883000
H	5.478817000	-1.739099000	1.162213000
C	4.957498000	-3.322259000	-0.217376000
H	5.976912000	-3.708420000	-0.286251000
C	3.935417000	-3.918601000	-0.953475000
H	4.149962000	-4.765854000	-1.608579000
C	2.638630000	-3.415354000	-0.884461000
H	1.852060000	-3.844281000	-1.511811000
B	0.673450000	-0.374258000	-0.061333000
H	0.415156000	-3.692850000	0.266557000

6.2.19 9B-2

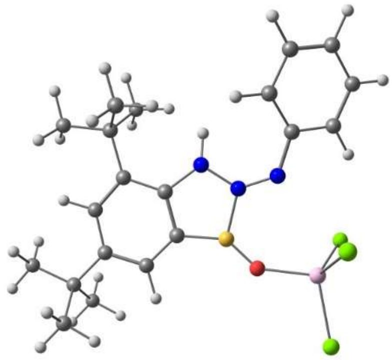

9B-2 at PBE0-D3(BJ)/Def2-SVP(SMD: benzene)

Cl	2.906759000	-4.500177000	0.214862000
Cl	3.592175000	-1.437717000	1.845683000
Cl	3.701330000	-1.675479000	-1.740012000
Al	2.787545000	-2.367027000	0.084809000
O	1.029053000	-1.981246000	0.015208000
N	1.994346000	0.731599000	-0.065838000
N	0.776100000	0.505322000	0.032955000
N	-0.243721000	1.365952000	0.110870000
C	-1.484011000	0.691041000	0.031282000
C	-1.304381000	-0.703336000	0.003296000
C	-2.414353000	-1.538417000	-0.040328000
H	-2.260784000	-2.619710000	-0.060162000
C	-3.705574000	-0.995465000	-0.064376000
C	-3.824982000	0.399537000	-0.037252000
H	-4.825347000	0.824830000	-0.053618000
C	-2.743546000	1.297083000	0.016012000
C	-4.921460000	-1.923121000	-0.118615000
C	-6.240022000	-1.150201000	-0.141102000
H	-7.082322000	-1.857453000	-0.181214000
H	-6.320430000	-0.495017000	-1.022589000
H	-6.373611000	-0.533348000	0.761256000
C	-4.839453000	-2.781639000	-1.388900000
H	-5.702341000	-3.464198000	-1.446133000
H	-3.928093000	-3.397777000	-1.411319000
H	-4.842328000	-2.153210000	-2.293285000
C	-4.911610000	-2.834116000	1.117271000
H	-5.777606000	-3.514880000	1.098328000
H	-4.962983000	-2.243984000	2.045655000
H	-4.003863000	-3.454342000	1.163619000
C	-2.949549000	2.815869000	0.056059000
C	-2.343760000	3.465085000	-1.199214000
H	-2.529590000	4.550469000	-1.191053000
H	-2.795936000	3.050035000	-2.112574000
H	-1.253836000	3.334826000	-1.292389000
C	-2.309450000	3.396712000	1.326677000
H	-2.458564000	4.487226000	1.361844000

H	-1.227458000	3.209408000	1.391474000
H	-2.767441000	2.962165000	2.228116000
C	-4.434957000	3.183623000	0.082835000
H	-4.962897000	2.843538000	-0.820828000
H	-4.538493000	4.278007000	0.131116000
H	-4.946962000	2.765138000	0.962376000
C	2.579607000	1.984511000	-0.068602000
C	3.847511000	2.004647000	-0.680820000
H	4.224090000	1.068211000	-1.098932000
C	4.566248000	3.188871000	-0.747132000
H	5.540679000	3.204886000	-1.239614000
C	4.053835000	4.348511000	-0.162563000
H	4.626866000	5.277905000	-0.200425000
C	2.820508000	4.321794000	0.492524000
H	2.441457000	5.222046000	0.981090000
C	2.069743000	3.154005000	0.536556000
H	1.138076000	3.146110000	1.104749000
B	0.212632000	-0.986757000	0.018962000
H	-0.097569000	2.321475000	-0.201119000

6.2.20 9B-3

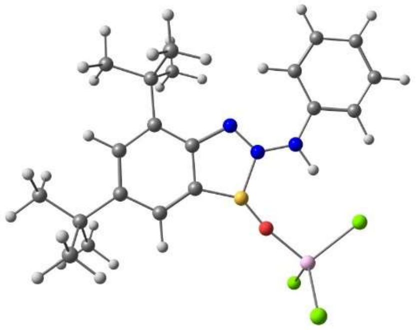

9B-3 at PBE0-D3(BJ)/Def2-SVP(SMD: benzene)

Cl	2.809828000	-3.712583000	-1.870005000
Cl	2.661954000	-3.972135000	1.706250000
Cl	4.308652000	-1.224549000	0.176799000
Al	2.722726000	-2.705289000	0.000411000
O	1.247589000	-1.702466000	0.006870000
N	2.000441000	1.143486000	0.028010000
N	0.751245000	0.765340000	0.027455000
N	-0.237945000	1.547655000	0.022277000
C	-1.421342000	0.775389000	0.013011000
C	-1.192100000	-0.615407000	0.008157000
C	-2.254332000	-1.500243000	-0.001788000
H	-2.060801000	-2.575407000	-0.005399000
C	-3.574270000	-1.006373000	-0.008580000
C	-3.756001000	0.379453000	-0.002038000
H	-4.774373000	0.760259000	-0.005660000
C	-2.708602000	1.323230000	0.008809000
C	-4.745429000	-1.988515000	-0.021135000
C	-4.638128000	-2.878166000	-1.268457000
H	-3.702735000	-3.457249000	-1.284397000
H	-4.677524000	-2.276471000	-2.189884000
H	-5.472396000	-3.596964000	-1.295676000
C	-4.675003000	-2.862197000	1.240115000
H	-4.743135000	-2.248919000	2.152167000
H	-3.738847000	-3.438248000	1.291160000
H	-5.508307000	-3.582610000	1.251390000
C	-6.098644000	-1.278821000	-0.046694000
H	-6.250798000	-0.646159000	0.841428000
H	-6.906918000	-2.025557000	-0.058731000
H	-6.218674000	-0.651032000	-0.943307000
C	-2.974914000	2.829868000	0.014938000
C	-4.471999000	3.144538000	0.009713000
H	-4.984393000	2.744202000	0.898052000
H	-4.976302000	2.753911000	-0.887517000
H	-4.612905000	4.235889000	0.014984000
C	-2.353010000	3.463330000	-1.239458000
H	-2.793842000	3.037681000	-2.154394000
H	-1.266770000	3.305882000	-1.283051000

H	-2.542409000	4.548516000	-1.247168000
C	-2.364794000	3.450751000	1.281319000
H	-1.279084000	3.292079000	1.333539000
H	-2.814358000	3.016442000	2.187907000
H	-2.553600000	4.535936000	1.297820000
C	2.534203000	2.429874000	0.006588000
C	3.939690000	2.493026000	-0.029244000
H	4.519145000	1.567240000	-0.038851000
C	4.576443000	3.723275000	-0.053531000
H	5.667659000	3.759928000	-0.081601000
C	3.830242000	4.904497000	-0.043336000
H	4.334601000	5.872958000	-0.063111000
C	2.440290000	4.837549000	-0.007574000
H	1.848265000	5.755568000	0.001194000
C	1.777278000	3.612254000	0.018029000
H	0.692649000	3.572770000	0.047125000
B	0.340012000	-0.796836000	0.014917000
H	2.658628000	0.350330000	0.048091000

6.2.21 PhBO·AlCl$_3$

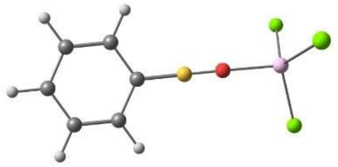

PhBO·AlCl$_3$ at PBE0-D3(BJ)/Def2-SVP(SMD: benzene)

B	0.938960000	-0.016966000	0.006129000
O	-0.285186000	-0.022468000	0.008291000
C	2.437366000	-0.011036000	0.004446000
C	3.134149000	1.212988000	0.002208000
C	3.145216000	-1.228673000	0.004594000
C	4.523584000	1.211703000	0.000321000
C	4.534550000	-1.214651000	0.002624000
C	5.219488000	0.001660000	0.000528000
H	5.068384000	2.158112000	-0.001336000
H	5.087942000	-2.156065000	0.002704000
Al	-2.156222000	0.000943000	-0.000992000
Cl	-2.639937000	-1.357516000	-1.556998000
Cl	-2.654204000	-0.655239000	1.953716000
Cl	-2.588871000	2.038185000	-0.407365000
H	6.312358000	0.006523000	-0.000970000
H	2.606787000	-2.179051000	0.006152000
H	2.587186000	2.158543000	0.002019000

6.2.22 [PhBO]$_3$

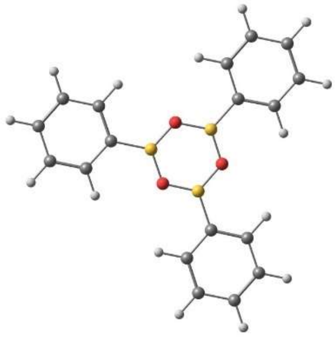

[PhBO]$_3$ at PBE0-D3(BJ)/Def2-SVP(SMD: benzene)

O	1.339278000	0.276438000	-0.000347000
B	0.434958000	1.313252000	-0.000935000
B	0.919601000	-1.033758000	-0.000503000
O	-0.909551000	1.021619000	-0.001527000
O	-0.430458000	-1.298415000	-0.000846000
B	-1.355347000	-0.279935000	-0.001176000
C	0.923999000	2.788220000	-0.000366000
C	2.295629000	3.090543000	0.000987000
C	0.005331000	3.850685000	-0.001119000
C	2.737162000	4.411218000	0.001608000
C	0.441162000	5.173271000	-0.000579000
C	1.808706000	5.453694000	0.000813000
H	3.021790000	2.273090000	0.001558000
H	-1.065545000	3.629857000	-0.002178000
H	3.807705000	4.632125000	0.002679000
H	-0.284805000	5.990492000	-0.001201000
H	2.153129000	6.491371000	0.001286000
C	-2.877304000	-0.594028000	-0.000644000
C	-3.338029000	-1.920903000	0.000497000
C	-3.825102000	0.442558000	-0.001169000
C	-4.701305000	-2.204892000	0.001106000
C	-5.189576000	0.164471000	-0.000598000
C	-5.628019000	-1.160873000	0.000552000
H	-2.611421000	-2.737943000	0.000917000
H	-3.480589000	1.480228000	-0.002041000
H	-5.045948000	-3.242216000	0.002006000
H	-5.916245000	0.981068000	-0.001023000
H	-6.698884000	-1.381548000	0.001030000
C	1.952815000	-2.194540000	0.000042000
C	3.332119000	-1.929279000	0.000795000
C	1.529753000	-3.533880000	-0.000157000
C	4.260284000	-2.967388000	0.001360000
C	2.453417000	-4.575986000	0.000372000
C	3.820252000	-4.292219000	0.001150000
H	3.675596000	-0.891254000	0.000927000
H	0.458973000	-3.755033000	-0.000756000

H	5.330848000	-2.746588000	0.001946000
H	2.110147000	-5.613764000	0.000198000
H	4.547276000	-5.108864000	0.001586000

6.2.23 [PhBO]$_3$·AlCl$_3$

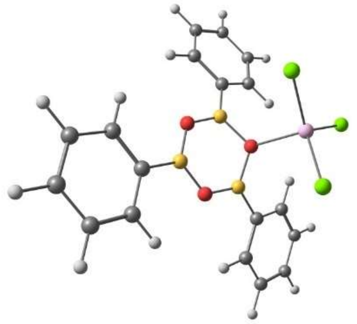

[PhBO]$_3$·AlCl$_3$ at PBE0-D3(BJ)/Def2-SVP(SMD: benzene)

O	-0.670584000	-0.000364000	-0.099482000
B	-0.014444000	-1.229766000	-0.497154000
B	-0.014273000	1.227956000	-0.499961000
O	1.336804000	-1.179159000	-0.570621000
O	1.336768000	1.176335000	-0.576090000
B	2.049237000	-0.001175000	-0.456526000
C	-0.836894000	-2.457750000	-0.922179000
C	-0.165381000	-3.657800000	-1.215277000
C	-2.234794000	-2.418027000	-1.056341000
C	-0.869867000	-4.785811000	-1.621275000
C	-2.941423000	-3.544937000	-1.464456000
C	-2.259138000	-4.728951000	-1.745263000
H	0.922159000	-3.700557000	-1.117266000
H	-2.781798000	-1.499358000	-0.833355000
H	-0.338136000	-5.714547000	-1.841659000
H	-4.028648000	-3.501604000	-1.561839000
H	-2.814436000	-5.615314000	-2.062915000
C	3.575107000	-0.000640000	-0.235416000
C	4.283518000	-1.208970000	-0.122867000
C	4.283661000	1.208156000	-0.128960000
C	5.658931000	-1.210419000	0.087835000
C	5.659079000	1.210496000	0.081717000
C	6.346104000	0.000262000	0.189689000
H	3.743474000	-2.156200000	-0.199581000
H	3.743744000	2.155059000	-0.210470000
H	6.200100000	-2.155655000	0.175357000
H	6.200380000	2.156089000	0.164437000
H	7.426499000	0.000617000	0.356509000
C	-0.836408000	2.456628000	-0.923658000
C	-0.164378000	3.656455000	-1.216503000
C	-2.234472000	2.418004000	-1.056352000
C	-0.868520000	4.785327000	-1.620688000
C	-2.940761000	3.545767000	-1.462712000
C	-2.257972000	4.729563000	-1.743171000
H	0.923292000	3.698383000	-1.119606000
H	-2.781913000	1.499551000	-0.833573000
H	-0.336387000	5.713903000	-1.840776000

H	-4.028123000	3.503263000	-1.558926000
H	-2.813008000	5.616615000	-2.059355000
Al	-1.494226000	0.000941000	1.649846000
Cl	-3.596473000	0.001249000	1.409681000
Cl	-0.647160000	-1.773089000	2.453964000
Cl	-0.645610000	1.774419000	2.453298000

6.2.24 BEt_3

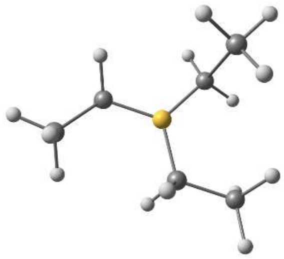

BEt_3 at M06-2X/6-311G(d,p)(IEFPCM: dichloromethane)

B	0.124565000	-0.093984000	-0.335536000
C	1.614361000	-0.606070000	-0.380087000
H	1.651711000	-1.653707000	-0.054966000
H	1.848796000	-0.656698000	-1.456403000
C	2.683562000	0.220353000	0.340272000
H	2.706548000	1.248444000	-0.028821000
H	3.682829000	-0.199912000	0.206576000
H	2.486528000	0.266240000	1.414541000
C	-1.026900000	-1.115685000	-0.669005000
H	-1.890560000	-0.642680000	-1.145625000
H	-0.684727000	-1.925946000	-1.320401000
C	-1.476479000	-1.723782000	0.681745000
H	-2.280422000	-2.448924000	0.541836000
H	-1.840896000	-0.952407000	1.365539000
H	-0.648994000	-2.238402000	1.177326000
C	-0.195840000	1.379403000	0.122227000
H	0.117827000	1.440349000	1.176542000
H	0.496797000	2.062517000	-0.387242000
C	-1.636639000	1.874596000	-0.028134000
H	-2.337467000	1.229255000	0.507688000
H	-1.941384000	1.879723000	-1.077653000
H	-1.761796000	2.889172000	0.356625000

6.2.25 [HBEt$_3$]$^-$

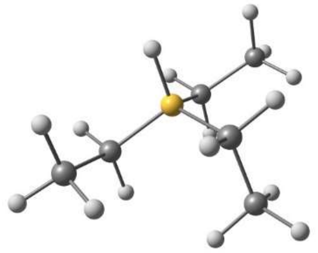

[HBEt$_3$]$^-$ at M06-2X/6-311G(d,p)(IEFPCM: dichloromethane)

B	0.008102000	-0.475067000	0.380559000
C	1.359877000	-0.909867000	-0.452761000
H	1.356529000	-1.997738000	-0.621748000
H	1.348027000	-0.464234000	-1.460911000
C	2.679800000	-0.525485000	0.226850000
H	2.771226000	0.562656000	0.310928000
H	3.571432000	-0.886020000	-0.300501000
H	2.718038000	-0.924231000	1.247378000
C	-0.009919000	1.142503000	0.704210000
H	-0.882268000	1.398061000	1.323627000
H	0.862810000	1.421550000	1.312938000
C	-0.029880000	2.028514000	-0.549459000
H	0.849513000	1.837510000	-1.174529000
H	-0.046217000	3.103083000	-0.330416000
H	-0.908835000	1.808643000	-1.165858000
C	-1.340256000	-0.942977000	-0.441486000
H	-1.331887000	-0.528768000	-1.462904000
H	-1.328639000	-2.035371000	-0.577465000
C	-2.663638000	-0.547968000	0.225493000
H	-2.696902000	-0.908121000	1.260557000
H	-3.551625000	-0.937827000	-0.286934000
H	-2.766678000	0.541538000	0.268632000
H	0.019058000	-1.083708000	1.477336000

6.2.26 Compound 1 at M06-2X/6-311G(d,p)(IEFPCM: dichloromethane)

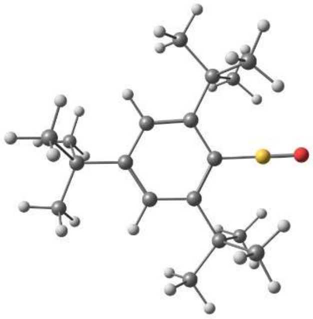

Compound 1 at M06-2X/6-311G(d,p)(IEFPCM: dichloromethane)

B	2.631368000	0.119274000	0.000144000
O	3.838213000	0.181866000	0.000198000
C	1.113635000	0.041071000	0.000005000
C	0.480896000	-1.222986000	-0.000036000
C	0.351982000	1.238195000	-0.000014000
C	-0.914835000	-1.261463000	-0.000031000
C	-1.033311000	1.128865000	-0.000018000
C	-1.688112000	-0.104972000	-0.000007000
H	-1.412211000	-2.217816000	-0.000045000
H	-1.635326000	2.025773000	-0.000026000
C	1.287305000	-2.535843000	-0.000040000
C	2.161396000	-2.622293000	1.265647000
H	2.924916000	-1.844841000	1.313737000
H	1.540495000	-2.550155000	2.161959000
H	2.679176000	-3.584777000	1.284166000
C	0.376324000	-3.771773000	-0.000280000
H	-0.258450000	-3.809387000	-0.888747000
H	1.001406000	-4.666923000	-0.000231000
H	-0.258753000	-3.809511000	0.887966000
C	1.882139000	2.799274000	1.265449000
H	1.271281000	2.667126000	2.161824000
H	2.718920000	2.101345000	1.314572000
C	-0.011154000	3.761652000	-0.000061000
H	-0.646518000	3.733752000	0.888395000
H	0.518456000	4.716426000	-0.000161000
H	-0.646623000	3.733619000	-0.888438000
C	1.021041000	2.625300000	-0.000040000
C	1.882128000	2.799220000	-1.265546000
H	1.271266000	2.667009000	-2.161909000
H	2.302249000	3.808170000	-1.283046000
H	2.718922000	2.101304000	-1.314639000
H	2.302280000	3.808217000	1.282890000
C	2.161767000	-2.622098000	-1.265483000
H	2.679557000	-3.584578000	-1.283991000
H	1.541131000	-2.549832000	-2.161969000
H	2.925308000	-1.844648000	-1.313244000

C	-3.218223000	-0.138101000	0.000027000
C	-3.738761000	0.582013000	-1.256204000
C	-3.738688000	0.581945000	1.256329000
H	-3.374341000	0.089805000	2.161538000
H	-3.422095000	1.626491000	1.284699000
H	-4.831623000	0.560424000	1.268940000
H	-3.422166000	1.626560000	-1.284541000
H	-4.831697000	0.560499000	-1.268748000
H	-3.374474000	0.089918000	-2.161462000
C	-3.770703000	-1.566900000	0.000004000
H	-3.455103000	-2.121128000	0.887556000
H	-4.862306000	-1.529708000	0.000045000
H	-3.455169000	-2.121075000	-0.887604000

6.2.27 Compound [1-H]⁻

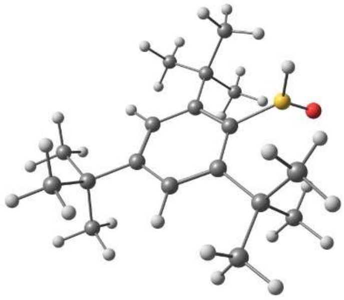

Compound [1-H]⁻ at M06-2X/6-311G(d,p)(IEFPCM: dichloromethane)

B	-2.761344000	0.133652000	0.439634000
O	-3.695785000	0.172442000	-0.445403000
C	-1.149150000	0.045800000	0.173589000
C	-0.484391000	-1.203051000	0.096351000
C	-0.351689000	1.221691000	0.097247000
C	0.916287000	-1.253780000	0.038185000
C	1.039420000	1.118036000	0.040110000
C	1.701695000	-0.108608000	0.030824000
H	1.409016000	-2.212843000	-0.001053000
H	1.641511000	2.015612000	-0.000020000
C	-1.266446000	-2.534761000	0.005138000
C	-2.209941000	-2.732140000	1.204976000
H	-2.642645000	-3.736697000	1.165215000
H	-1.663195000	-2.632906000	2.147238000
H	-3.033374000	-2.020684000	1.212878000
C	-2.081630000	-2.542036000	-1.302170000
H	-1.407208000	-2.487989000	-2.162448000
H	-2.655426000	-3.472301000	-1.378576000
H	-2.766844000	-1.694420000	-1.339037000
C	0.060646000	3.751377000	-0.041192000
H	0.674774000	3.774964000	0.863638000
H	0.720945000	3.660757000	-0.907216000
C	-1.895554000	2.929449000	1.209900000
H	-1.355258000	2.776165000	2.148570000
H	-2.219180000	3.974164000	1.168194000
H	-2.789253000	2.309051000	1.226717000
C	-0.987507000	2.628246000	0.004437000
C	-1.807692000	2.718101000	-1.296624000
H	-2.582422000	1.950985000	-1.324163000
H	-2.276529000	3.705262000	-1.373251000
H	-1.150873000	2.586217000	-2.162009000
H	-0.455877000	4.711264000	-0.119835000
C	-0.345492000	-3.765132000	-0.028550000
H	0.323852000	-3.753424000	-0.892201000
H	0.259182000	-3.849258000	0.879024000
H	-0.962838000	-4.663815000	-0.103620000
C	3.233191000	-0.144923000	-0.010359000

C	3.782294000	-1.575437000	-0.013516000
C	3.796110000	0.580137000	1.223774000
H	3.459349000	0.091254000	2.141627000
H	4.890093000	0.564826000	1.206040000
H	3.472623000	1.622673000	1.257075000
H	3.475676000	-2.124071000	0.880828000
H	4.874807000	-1.545680000	-0.032502000
H	3.446253000	-2.132655000	-0.891810000
C	3.732781000	0.561744000	-1.282106000
H	3.412510000	1.605377000	-1.312230000
H	4.826149000	0.541747000	-1.321392000
H	3.346581000	0.062614000	-2.174586000
H	-2.996988000	0.157742000	1.663873000

6.2.28 Compound 7 at M06-2X/6-311G(d,p)(IEFPCM: dichloromethane)

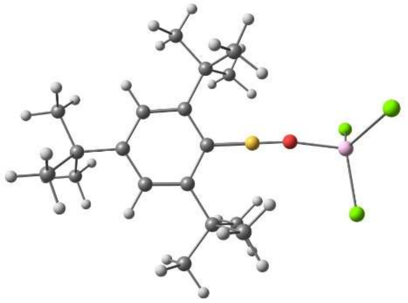

Compound 7 at M06-2X/6-311G(d,p)(IEFPCM: dichloromethane)

B	0.773031000	-0.160941000	-0.012348000
O	2.004320000	-0.210729000	-0.015843000
C	-0.714430000	-0.095828000	-0.007848000
C	-1.349195000	1.177587000	-0.005446000
C	-1.460035000	-1.301156000	-0.005296000
C	-2.734838000	1.198944000	-0.000783000
C	-2.848593000	-1.201088000	-0.000542000
C	-3.502950000	0.029273000	0.001753000
H	-3.247307000	2.149777000	0.000963000
H	-3.437849000	-2.103618000	0.001481000
C	-0.535998000	2.482116000	-0.008129000
C	0.341361000	2.561095000	1.255705000
H	0.880218000	3.511394000	1.265272000
H	-0.276921000	2.503203000	2.154248000
H	1.098573000	1.776191000	1.317460000
C	0.333213000	2.560491000	-1.277602000
H	0.870298000	3.511732000	-1.292158000
H	1.091630000	1.777027000	-1.342537000
H	-0.290688000	2.500116000	-2.172099000
C	0.090330000	-2.831051000	-1.276626000
H	0.529792000	-3.830932000	-1.295778000
H	-0.523142000	-2.706537000	-2.171635000
C	-1.784313000	-3.825498000	-0.004914000
H	-2.421647000	-3.805506000	-0.891779000
H	-1.240816000	-4.772019000	-0.006724000
H	-2.416508000	-3.805997000	0.885625000
C	-0.769245000	-2.674624000	-0.007527000
C	0.097375000	-2.831422000	1.256735000
H	0.537494000	-3.831071000	1.272844000
H	0.927707000	-2.123730000	1.313215000
H	-0.511236000	-2.707800000	2.155176000
H	0.920828000	-2.123898000	-1.337161000
C	-1.443160000	3.719656000	-0.005414000
H	-2.074264000	3.757289000	0.885426000
H	-0.816380000	4.613151000	-0.007460000
H	-2.079779000	3.757048000	-0.892336000

C	-5.027389000	0.143277000	0.006864000
C	-5.716074000	-1.224933000	0.009687000
C	-5.476033000	0.911162000	-1.249356000
H	-5.061362000	1.920564000	-1.278415000
H	-5.164098000	0.386036000	-2.155433000
H	-6.565728000	0.994121000	-1.257626000
H	-5.454588000	-1.806889000	0.897023000
H	-5.460768000	-1.807485000	-0.879054000
H	-6.798258000	-1.079489000	0.013386000
C	-5.467692000	0.912091000	1.265458000
H	-5.149886000	0.387553000	2.169834000
H	-5.052778000	1.921489000	1.291152000
H	-6.557299000	0.995152000	1.280794000
Al	3.813464000	0.006765000	0.003813000
Cl	4.077401000	2.114283000	-0.045688000
Cl	4.466652000	-0.887767000	1.812335000
Cl	4.519242000	-0.980331000	-1.735182000

6.2.29 Compound [7-H]⁻

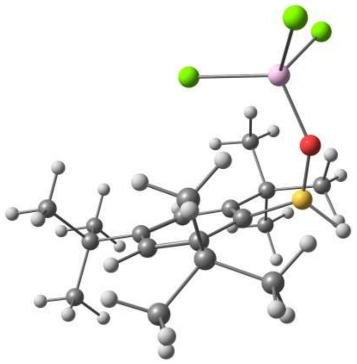

Compound [7-H]⁻ at M06-2X/6-311G(d,p)(IEFPCM: dichloromethane)

Cl	-4.193790000	-1.636590000	-1.069985000
Cl	-1.237213000	-0.078935000	-2.104622000
Al	-2.793517000	-0.058213000	-0.612875000
O	-2.245984000	-0.271229000	1.019781000
C	0.289908000	-0.035920000	1.188950000
C	0.864944000	1.203190000	0.838047000
C	2.054065000	1.229211000	0.092702000
H	2.462518000	2.188936000	-0.192228000
C	2.709572000	0.077804000	-0.306376000
C	2.159329000	-1.140046000	0.097235000
H	2.673231000	-2.047219000	-0.190011000
C	0.982248000	-1.229422000	0.834041000
C	0.289875000	2.596340000	1.182448000
C	-0.105558000	3.315300000	-0.119716000
H	0.749027000	3.441178000	-0.787796000
H	-0.510364000	4.306332000	0.107046000
H	-0.871714000	2.747933000	-0.654139000
C	1.364664000	3.424112000	1.915813000
H	2.256568000	3.584263000	1.308952000
H	0.959098000	4.405964000	2.175660000
C	0.469682000	-2.648303000	1.176028000
C	-0.443124000	-3.146161000	0.042260000
H	-1.330765000	-2.518207000	-0.047752000
H	-0.771626000	-4.170253000	0.246296000
C	-0.314270000	-2.703076000	2.497593000
H	-1.315261000	-2.278112000	2.412592000
H	0.217254000	-2.189943000	3.302838000
H	-0.453716000	-3.746913000	2.789418000
C	3.992144000	0.089657000	-1.142160000
C	5.125997000	-0.581735000	-0.348711000
H	6.048156000	-0.581496000	-0.937133000
H	5.312546000	-0.043415000	0.584210000
C	3.755398000	-0.688269000	-2.447976000
H	4.663095000	-0.681736000	-3.058430000
H	3.487476000	-1.728661000	-2.252530000
H	2.946816000	-0.233158000	-3.025618000

B	-1.170946000	-0.128871000	1.816932000
C	-0.937556000	2.581970000	2.098672000
H	-0.727671000	2.083570000	3.046917000
H	-1.219261000	3.614139000	2.322932000
H	-1.808250000	2.118159000	1.630432000
H	1.667680000	2.923822000	2.839628000
H	0.083490000	-3.133114000	-0.915310000
C	1.636143000	-3.645329000	1.321517000
H	2.135501000	-3.848307000	0.373225000
H	2.381141000	-3.282877000	2.034999000
H	1.247778000	-4.599123000	1.685634000
H	4.883517000	-1.617308000	-0.100555000
C	4.435464000	1.510705000	-1.504049000
H	5.346306000	1.464085000	-2.106054000
H	4.653412000	2.103165000	-0.611549000
H	3.672403000	2.031872000	-2.087944000
Cl	-3.823361000	1.838782000	-0.747076000
H	-1.349414000	-0.098180000	3.001441000

*6.2.30 [Mes*CO]+ at M06-2X/6-311G(d,p)(IEFPCM: dichloromethane)*

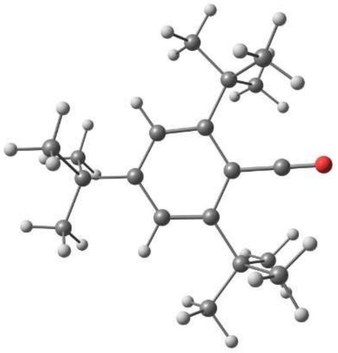

[Mes*CO]+ at M06-2X/6-311G(d,p)(IEFPCM: dichloromethane)

C	2.452412000	0.141267000	0.045944000
O	3.574560000	0.213718000	0.091097000
C	1.080934000	0.053781000	0.002179000
C	0.490478000	-1.253568000	-0.008400000
C	0.325386000	1.278300000	-0.007756000
C	-0.891850000	-1.285781000	-0.011143000
C	-1.043316000	1.128140000	-0.009550000
C	-1.674188000	-0.126543000	-0.003650000
H	-1.383170000	-2.244402000	-0.017957000
H	-1.662349000	2.011910000	-0.014597000
C	1.323405000	-2.539211000	-0.016463000
C	0.425565000	-3.783214000	-0.062869000
H	1.065141000	-4.666628000	-0.075138000
H	-0.219236000	-3.853182000	0.815536000
H	-0.191279000	-3.806077000	-0.963737000
C	2.218427000	-2.578704000	-1.271281000
H	1.610684000	-2.504296000	-2.175204000
H	2.975051000	-1.794158000	-1.306238000
H	2.751800000	-3.530856000	-1.293736000
C	1.868787000	2.811620000	-1.273730000
H	1.268803000	2.676312000	-2.175823000
H	2.289700000	3.818585000	-1.289538000
C	1.810875000	2.855292000	1.272798000
H	2.649042000	2.165778000	1.378417000
H	2.229250000	3.863570000	1.274571000
H	1.171218000	2.747304000	2.150890000
C	0.989321000	2.658730000	-0.016654000
C	-0.060058000	3.777712000	-0.060150000
H	0.459792000	4.736603000	-0.075941000
H	-0.678245000	3.719386000	-0.958441000
H	-0.704932000	3.765038000	0.821015000
H	2.710140000	2.118875000	-1.320858000
C	2.160962000	-2.630589000	1.274639000
H	2.904618000	-1.840178000	1.382076000
H	1.511496000	-2.606324000	2.151885000

H	2.703635000	-3.577790000	1.275584000
C	-3.195972000	-0.181023000	0.011155000
C	-3.732096000	-1.615405000	0.016478000
C	-3.729329000	0.540274000	-1.241827000
H	-3.434431000	1.590656000	-1.269249000
H	-3.369141000	0.055333000	-2.151927000
H	-4.820688000	0.498347000	-1.238724000
H	-3.426818000	-2.165397000	-0.876986000
H	-4.822854000	-1.584760000	0.030028000
H	-3.404726000	-2.166722000	0.901291000
C	-3.698404000	0.537309000	1.278865000
H	-3.400527000	1.586972000	1.301940000
H	-4.789539000	0.496892000	1.302047000
H	-3.316934000	0.049136000	2.178539000

*6.2.31 Mes*C(O)H*

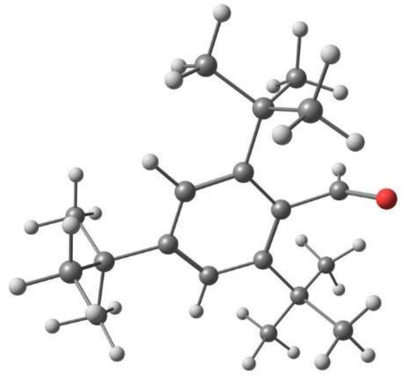

Mes*C(O)H at M06-2X/6-311G(d,p)(IEFPCM: dichloromethane)

C	2.605134000	0.146923000	-0.242321000
O	3.360810000	0.195364000	0.696553000
C	1.109095000	0.052684000	-0.128034000
C	0.497812000	-1.216644000	-0.093861000
C	0.342409000	1.241528000	-0.093288000
C	-0.899614000	-1.265973000	-0.036188000
C	-1.043153000	1.112825000	-0.036118000
C	-1.687891000	-0.123076000	-0.012648000
H	-1.385365000	-2.227930000	-0.015116000
H	-1.653436000	2.003794000	-0.014003000
C	1.298453000	-2.536313000	-0.055150000
C	1.917168000	-2.700956000	1.346246000
H	2.585221000	-1.875397000	1.591451000
H	1.129981000	-2.741402000	2.103458000
H	2.485537000	-3.634592000	1.392136000
C	0.396752000	-3.756235000	-0.307149000
H	-0.112750000	-3.689844000	-1.272164000
H	1.016795000	-4.654741000	-0.311971000
H	-0.351869000	-3.885186000	0.476626000
C	1.574750000	2.887892000	1.342974000
H	2.338936000	2.149748000	1.585854000
H	2.026071000	3.883477000	1.386270000
C	-0.072178000	3.747993000	-0.302162000
H	-0.827092000	3.782086000	0.485463000
H	0.431396000	4.716609000	-0.308652000
H	-0.574037000	3.619488000	-1.264961000
C	0.974545000	2.649024000	-0.055702000
C	2.066113000	2.833751000	-1.129649000
H	3.008334000	2.351712000	-0.870607000
H	1.733460000	2.462239000	-2.102431000
H	2.288229000	3.898285000	-1.233041000
H	0.791488000	2.833114000	2.103307000
C	2.409573000	-2.585099000	-1.123913000
H	2.760084000	-3.614541000	-1.227130000
H	2.038731000	-2.255554000	-2.097976000

H	3.285088000	-1.992551000	-0.860140000
C	-3.217781000	-0.172252000	0.036909000
C	-3.708922000	0.543068000	1.307164000
C	-3.752690000	-1.607645000	0.056666000
H	-3.402437000	-2.155760000	0.935072000
H	-4.844359000	-1.584934000	0.089978000
H	-3.456601000	-2.160035000	-0.838849000
H	-3.310296000	0.057002000	2.201209000
H	-4.800826000	0.510064000	1.354855000
H	-3.402796000	1.591123000	1.324206000
C	-3.788586000	0.538389000	-1.202364000
H	-3.481166000	1.585450000	-1.244316000
H	-3.449994000	0.046863000	-2.117992000
H	-4.881385000	0.508381000	-1.179306000
H	3.002650000	0.170820000	-1.272147000

6.3 Aromaticity study of 9

6.3.1 Computational methods

A truncated structure (in which t-butyl and phenyl substituents were substituted for a proton, see Figure S45-a) was used to investigate the magnetic response properties of the **9** system. The geometry was optimised and verified to be a minimum energy structure through vibrational analysis, with ωB97x-D/cc-pVDZ using Q-Chem 6.1 computational package.[S36]

Current-density maps were calculated at the CHF/CTOCD-DZ2/6-31G(d,p) level, with the Modena/Exeter/Sheffield version of the SYSMO package.[S37] The combination of DFT geometries and CHF currents have previously been demonstrated to give excellent results.[S38]

Figure S45-b and -c shows the total (σ+π) current density and the 'π-only' current density, respectively. The 'π-only' map refers to current arising from transitions between the occupied HOMO and HOMO-1 orbitals to the virtual manifold, while the system has low symmetry (C_1) these molecular orbitals are confirmed by visual inspection to correspond to π character. Full details of the ipsocentric method can be found elsewhere,[S39,S40] along with conventions for plotting conventions and interpretation of ring-current maps. Briefly, current is plotted at $1a_0$ above the plane of the cycle, with contours showing the full modulus and arrows showing the magnitude and direction of the in-plane component of the induced current density per external field. Aromaticity is diagnosed by a diatropic current with counter-clockwise circulation, while antiaromaticity has a paratropic current with clockwise circulation. Nuclear positions are projected onto the plotting plane and follow the same colour scheme as the structure in Figure S45, where carbon is grey, boron is pink, nitrogen is blue, chlorine is green, and aluminium is beige.

6.3.2 Results & Discussion

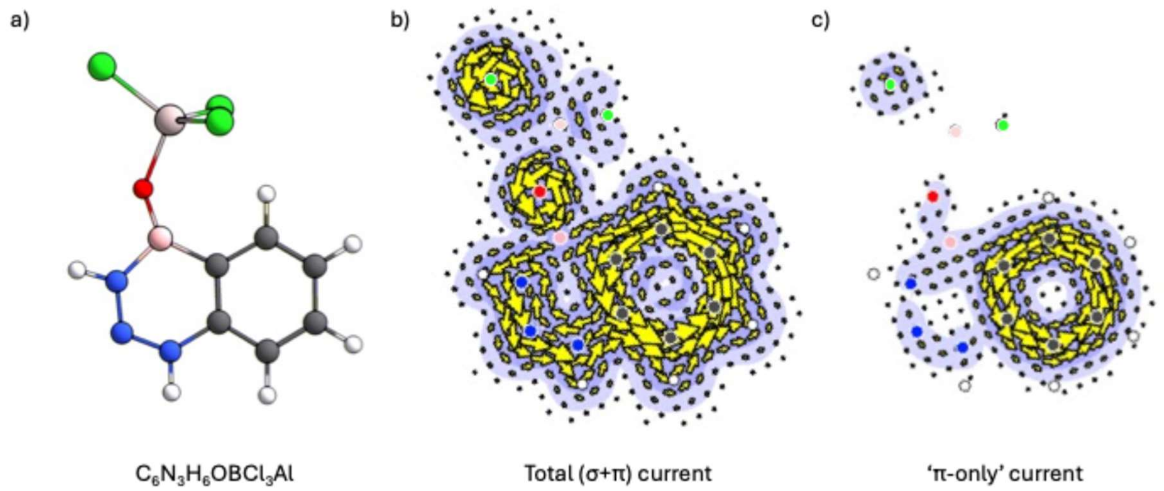

Figure S45: a. A schematic showing the truncated structure used to consider the magnetic response properties. **b.** the total ring-current map **c.** the 'π-only' frontier orbital dominated ring-current map.

Exploration of the magnetic response properties of the compound **9** hetero-bicyclic system revealed delocalisation localised to the all-carbon Clar's sextet. Figure S45-b shows (i) diatropic delocalisation around the all-carbon six-membered cycle, (ii) localised diatropic circulations around electronegative centres (Cl and O) and (iii) delocalisation around the N-N bonds in the six-membered heterocycle. A specific advantage of the ipsocentric approach to modelling ring-current is the ability to partition global ring-currents into specific occupied-to-virtual transitions. The current arising from the HOMO and HOMO-1 transitions to unoccupied orbitals is plotted in Figure S45-c showing the classic diatropic signature of an aromatic system. The current is localised to the carbon six-membered cycle and despite the bond alternation, the ring-current map is essentially indistinguishable from that of benzene. There is no compelling evidence of a π current in the heterocycle, and as such this cycle is nonaromatic. The apparent delocalisation about the N-N bonds (Figure S45-b) is attributed to partial double-bond character.

6.3.3 Cartesian coordinates of the optimised structure

17	-2.029378121	-1.406139127	1.675525898
17	-1.757437324	-1.081647367	-1.873778737
17	-3.934039951	1.166519390	-0.031432310
13	-2.157709885	-0.041333829	-0.009354116
8	-0.741691928	1.104302198	0.181224676
7	1.238179864	2.433484543	0.129673446
7	2.489862810	2.702622748	0.065181245
7	3.290143936	1.702469816	0.005995317
6	2.969724324	0.331366855	0.008056461
6	1.622315211	-0.054023034	0.083301312
6	1.348945174	-1.432674350	0.091324609
1	0.314591165	-1.770589443	0.159202232
6	2.379303573	-2.358390184	0.019193499
6	3.714223467	-1.937091269	-0.062959071
1	4.516458562	-2.673300340	-0.126208564
6	4.023962295	-0.587340318	-0.066901474
1	2.148468851	-3.424167875	0.024414122
1	5.060939895	-0.249722393	-0.128133644
1	0.672099804	3.280973937	0.167051177
5	0.556526294	1.075060845	0.137440223
1	4.264965382	1.984838905	-0.045114872

7 References

S1. P. Gupta, J.-E. Siewert, T. Wellnitz, M. Fischer, W. Baumann, T. Beweries, C. Hering Junghans, *Dalton Trans.* **2021**, 50, 1838-1844.

S2. V. M. Hipwell, M. A. Garcia-Garibay, *J. Org. Chem.* **2019**, 84, 17, 11103-11113.

S3. J. E. Borger, A. W. Ehlers, M. Lutz, J. C. Slootweg, K. Lammertsma, *Angew. Chem. Int. Ed.* **2014**, 53, 12836-12839.

S4. P. Lenz, R. Oshimizu, S. Klabunde, C. G. Daniliuc, C. Mück-Lichtenfeld, J. C. Tendyck, T. Mori, W. Uhl, M. R. Hansen, H. Eckert, S. Yamaguchi, A. Studer, *Angew. Chem. Int. Ed.* **2022**, 61, e202209391.

S5. M. Groteklaes, P. Paetzold, *Chem. Ber.* **1988**, 121, 809-810.

S6. B. Pachaly, R. West, *J. Am. Chem. Soc.* **1985**, 107, 2987-2988.

S7. K. Usui, K. Tanoue, K. Yamamoto, T. Shimizo, H. Suemune, *Org. Lett.* **2014**, 16, 4662-4665.

S8. D. B. Bolstad, E. S. D. Bolstad, K. M. Frey, D. L. Wright, A. C. Anderson, *J. Med. Chem.* **2008**, 51, 6839-6852.

S9. D. M. Lustosa, S. Barkai, I. Domb, A. Milo, *J. Org. Chem.* **2022**, 87, 1850-1857.

S10. R. Neufeld, D. Stalke, *Chem. Sci.,* **2015**, 6, 3354-3364.

S11. S. Bachmann, R. Neufeld, M. Dzemski, D. Stalke, *Chem. Eur. J.,* **2016**, 22, 8462-8465.

S12. P.-O. Augé S., Schmit, C. A. Crutchfield, M. T. Islam, D. J. Harris, E. Durand, M. Clemancey, A.-A. Quoineaud, J.-M. Lancelin, Y. Prigent, F. Taulelle, M.-A. Delsuc, *J. Phys. Chem. B* **2009**, 113, 1914–1918.

S13. A. Y. Jordan, T. Y. Meyer, *J. Organomet. Chem.*, **1999**, 591, 104-113.

S14. O. V. Dolomanov, L. J. Bourhis, R. J. Gildea, J. A. K. Howard, H. Puschmann, *J. Appl. Cryst.* **2009**, 42, 339-341.

S15. G. M. Sheldrick, *Acta Cryst.* **2015**, C71, 3-8.

S16. G. M. Sheldrick, *Acta Cryst.* **2008**, A64, 112-122.

S17. G. M. Sheldrick, *Acta Cryst.* **2015**, A71, 3-8.

S18. N. T. Johnson, P. G. Waddel, W. Clegg, M. R. Probert, *Crystals*, **2017**, 7(12), 360.

S19. M. J. Frisch, G. W. Trucks, H. B. Schlegel, G. E. Scuseria, M. A. Robb, J. R. Cheeseman, G. Scalmani, V. Barone, G. A. Petersson, H. Nakatsuji, X. Li, M. Caricato, A. V. Marenich, J. Bloino, B. G. Janesko, R. Gomperts, B. Mennucci, H. P. Hratchian, J. V. Ortiz, A. F. Izmaylov, J. L. Sonnenberg, Williams, F. Ding, F. Lipparini, F. Egidi, J. Goings, B. Peng, A. Petrone, T. Henderson, D. Ranasinghe, V. G. Zakrzewski, J. Gao, N. Rega, G. Zheng, W. Liang, M. Hada, M. Ehara, K. Toyota, R. Fukuda, J. Hasegawa, M. Ishida, T. Nakajima, Y. Honda, O. Kitao, H. Nakai, T. Vreven, K. Throssell, J. A. Montgomery Jr., J. E. Peralta, F. Ogliaro, M. J. Bearpark, J. J. Heyd, E. N. Brothers, K. N. Kudin, V. N. Staroverov, T. A. Keith, R. Kobayashi, J. Normand, K. Raghavachari, A. P. Rendell, J. C. Burant, S. S. Iyengar, J. Tomasi, M. Cossi, J. M. Millam,

M. Klene, C. Adamo, R. Cammi, J. W. Ochterski, R. L. Martin, K. Morokuma, O. Farkas, J. B. Foresman, D. J. Fox, Gaussian 16 Rev. C.01, **2016**.

S20. P. Pracht, F. Bohle, S. Grimme, *Phys. Chem. Chem. Phys.* **2020**, *22*, 7169–7192.

S21. C. Bannwarth, S. Ehlert, S. Grimme, *J. Chem. Theory Comput.* **2019**, *15*, 1652–1671.

S22. F. Weigend, R. Ahlrichs, *Phys. Chem. Chem. Phys.* **2005**, *7*, 3297–3305.

S23. C. Adamo, V. Barone, *The Journal of Chemical Physics* **1999**, *110*, 6158–6170.

S24. S. Grimme, S. Ehrlich, L. Goerigk, *Journal of Computational Chemistry* **2011**, *32*, 1456–1465.

S25. A. V. Marenich, C. J. Cramer, D. G. Truhlar, *J. Phys. Chem. B* **2009**, *113*, 6378–6396.

S26. H. S. Yu, X. He, S. L. Li, D. G. Truhlar, *Chem. Sci.* **2016**, *7*, 5032–5051.

S27. K. Wolinski, J. F. Hinton, P. Pulay, *J. Am. Chem. Soc.* **1990**, *112*, 8251–8260.

S28. T. Lu, F. Chen, *Journal of Computational Chemistry* **2012**, *33*, 580–592.

S29. Chemcraft - graphical software for visualization of quantum chemistry computations. Version 1.8, build 682. https://www.chemcraftprog.com.

S30. M. D. Hanwell, D. E. Curtis, D. C. Lonie, T. Vandermeersch, E. Zurek, G. R. Hutchison, *Journal of Cheminformatics* **2012**, *4*, 17.

S31. E. R. Clark, A. Del Grosso, M. J. Ingleson, *Chemistry – A European Journal* **2013**, *19*, 2462–2466.

S32. Y. Zhao, D. G. Truhlar, *Theor Chem Account* **2008**, *120*, 215–241.

S33. M. Cossi, V. Barone, R. Cammi, J. Tomasi, *Chemical Physics Letters* **1996**, *255*, 327–335.

S34. R. Krishnan, J. S. Binkley, R. Seeger, J. A. Pople, *The Journal of Chemical Physics* **1980**, *72*, 650–654.

S35. A. D. McLean, G. S. Chandler, *The Journal of Chemical Physics* **1980**, *72*, 5639–5648.

S36. E. Epifanovsky, A. T. B. Gilbert, X. Feng, J. Lee, Y. Mao, N. Mardirossian, P. Pokhilko, A. F. White, M. P. Coons, A. L. Dempwolff, Z. Gan, D. Hait, P. R. Horn, L. D. Jacobson, I. Kaliman, J. Kussmann, A. W. Lange, K. U. Lao, D. S. Levine, J. Liu, S. C. McKenzie, A. F. Morrison, K. D. Nanda, F. Plasser, D. R. Rehn, M. L. Vidal, Z.-Q. You, Y. Zhu, B. Alam, B. J. Albrecht, A. Aldossary, E. Alguire, J. H. Andersen, V. Athavale, D. Barton, K. Begam, A. Behn, N. Bellonzi, Y. A. Bernard, E. J. Berquist, H. G. A. Burton, A. Carreras, K. Carter-Fenk, R. Chakraborty, A. D. Chien, K. D. Closser, V. Cofer-Shabica, S. Dasgupta, M. de Wergifosse, J. Deng, M. Diedenhofen, H. Do, S. Ehlert, P.-T. Fang, S. Fatehi, Q. Feng, T. Friedhoff, J. Gayvert, Q. Ge, G. Gidofalvi, M. Goldey, J. Gomes, C. E. González-Espinoza, S. Gulania, A. O. Gunina, M. W. D. H.-H., P. H. P. Harbach, A. Hauser, M. F. Herbst, M. Hernández Vera, M. Hodecker, Z. C. Holden, S. Houck, X. Huang, K. Hui, B. C. Huynh, M. Ivanov, Á. Jász, H. Ji, H. Jiang, B. Kaduk, S. Kähler, K. Khistyaev, J. Kim, G. Kis, P. Klunzinger, Z. Koczor-Benda, J. Hoon Koh, D. Kosenkov, L. Koulias, T. Kowalczyk, C. M. Krauter, K. Kue, A. Kunitsa, T. Kus, I. Ladjánszki, A. Landau, K. V. Lawler, D. Lefrancois, S. Lehtola, R. R. Li, Y.-P. Li, J. Liang, M. Liebenthal, H.-H. Lin, Y.-S. Lin, F. Liu, K.-Y. Liu, M. Loipersberger, A. Luenser, A. Manjanath, P. Manohar, E. Mansoor, S. F. Manzer, S.-P. Mao, A. V. Marenich, T. Markovich, S. Mason, S. A. Maurer, P. F. McLaughlin, M. F. S. J. Menger, J.-M. Mewes, S. A. Mewes, P. Morgante, J. W. Mullinax, K.

J. Oosterbaan, G. Paran, A. C. Paul, S. K. Paul, F. Pavošević, Z. Pei, S. Prager, E. I. Proynov, Á. Rák, E. Ramos-Cordoba, B. Rana, A. E. Rask, A. Rettig, R. M. Richard, F. Rob, E. Rossomme, T. Scheele, M. Scheurer, M. Schneider, N. Sergueev, S. M. Sharada, W. Skomorowski, D. W. Small, C. J. Stein, Y.-C. Su, E. J. Sundstrom, Z. Tao, J. Thirman, G. J. Tornai, T. Tsuchimochi, N. M. Tubman, S. Prasad Veccham, O. Vydrov, J. Wenzel, J. Witte, A. Yamada, K. Yao, S. Yeganeh, S. R. Yost, A. Zech, I. Ying Zhang, X. Zhang, Y. Zhang, D. Zuev, A. Aspuru-Guzik, A. T. Bell, N. A. Besley, K. B. Bravaya, B. R. Brooks, D. Casanova, J.-D. Chai, S. Coriani, C. J. Cramer, G. Cserey, A. E. DePrince, III, R. A. DiStasio, Jr., A. Dreuw, B. D. Dunietz, T. R. Furlani, W. A. Goddard, III, S. Hammes-Schiffer, T. Head-Gordon, W. J. Hehre, C.-P. Hsu, T.-C. Jagau, Y. Jung, A. Klamt, J. Kong, D. S. Lambrecht, W. Liang, N. J. Mayhall, C. W. McCurdy, J. B. Neaton, C. Ochsenfeld, J. A. Parkhill, R. Peverati, V. A. Rassolov, Y. Shao, L. V. Slipchenko, T. Stauch, R. P. Steele, J. E. Subotnik, A. J. W. Thom, A. Tkatchenko, D. G. Truhlar, T. Van Voorhis, T. A. Wesolowski, K. B. Whaley, H. L. Woodcock, III, P. M. Zimmerman, S. Faraji, P. M. W. Gill, M. Head-Gordon, J. M. Herbert, A. I. Krylov, *J. Chem. Phys.* **2021**, 155, 084801.

S37. P. Lazzeretti, R. Zanasi, *SYSMO Package* (University of Modena: Modena, Italy, 1980). Additional routines by P. W. Fowler, E. Steiner, R. W. A. Havenith, A. Soncini.

S38. R. W. A. Havenith, A. J. H. M. Meijer, B. J. Irving, P. W. Fowler, *Molec. Phys.* **2009**, 107, 2591-2600.

S39. E. Steiner, P. W. Fowler, *J. Phys. Chem. A* **2001**, 105, 41, 9553-9562.

S40. E. Steiner, P. W. Fowler, *Chem. Commun.* **2001**, 2220-2221.
